# NMDA receptor-dependent plasticity in the nucleus accumbens connects reward-predictive cues to approach responses

**DOI:** 10.1038/s41467-019-12387-z

**Published:** 2019-09-27

**Authors:** Mercedes Vega-Villar, Jon C. Horvitz, Saleem M. Nicola

**Affiliations:** 1 Department of Psychology, The Graduate Center, City University of New York, 365 Fifth Avenue, 6th Floor, New York, NY 10016 USA; 2 Department of Psychology, City College of New York, City University of New York, 160 Convent Avenue, NAC 7/120, New York, NY 10031 USA; 3 Department of Neuroscience, Albert Einstein College of Medicine, Jack and Pearl Resnick Campus, 1300 Morris Park Avenue, Forchheimer Building, Room-111, Bronx, NY 10461 USA; 4 Department of Psychiatry, Albert Einstein College of Medicine, Bronx, NY 10461 USA

**Keywords:** Operant learning, Motivation, Reward

## Abstract

Learning associations between environmental cues and rewards is a fundamental adaptive function. Via such learning, reward-predictive cues come to activate approach to locations where reward is available. The nucleus accumbens (NAc) is essential for cued approach behavior in trained subjects, and cue-evoked excitations in NAc neurons are critical for the expression of this behavior. Excitatory synapses within the NAc undergo synaptic plasticity that presumably contributes to cued approach acquisition, but a direct link between synaptic plasticity within the NAc and the development of cue-evoked neural activity during learning has not been established. Here we show that, with repeated cue-reward pairings, cue-evoked excitations in the NAc emerge and grow in the trials prior to the detectable expression of cued approach behavior. We demonstrate that the growth of these signals requires NMDA receptor-dependent plasticity within the NAc, revealing a neural mechanism by which the NAc participates in learning of conditioned reward-seeking behaviors.

## Abstract (web summary)

Conditioned stimuli elicit phasic changes in nucleus accumbens (NAc) firing that invigorate approach responses to predicted rewards. Here the authors show that NAc neurons acquire cue-evoked responses during learning as a result of excitatory plasticity within the NAc.

## Introduction

Unexpected reward-predictive stimuli signal animals to interrupt ongoing behavior and move towards the predicted location of reward. In this form of adaptive behavior, cue-reward associations invigorate approach responses. This function requires the integration of motivational and motor neural systems, which has long been thought to depend on the nucleus accumbens (NAc)^1^. Accordingly, individual neurons in the NAc of trained animals exhibit cue-evoked excitations that encode the motivational value of the stimulus^2–6^ while simultaneously determining the vigor of the ensuing approach response^7–11^. NAc neurons become cue-responsive after training in appetitive conditioning paradigms^12,13^. If the role of the NAc is to allow reward-paired cues to access motor systems, then changes in cue-evoked NAc firing over the course of training should precede or accompany the emergence of cued approach behavior. However, despite evidence that the NAc participates in the acquisition of appetitive conditioned responses^14–19^, no study to date has examined the relationship between experience-dependent changes in NAc activity and cued approach learning.

Cued approach acquisition depends on unconstrained exploratory behavior to produce chance encounters with the reward in close temporal proximity to the cue. Consequently, the rate of learning is highly variable across individuals, which complicates the extraction of meaningful group data. In addition, although high rates of exploratory behavior facilitate learning, they obscure the extent to which responses are motivated by reward-predictive cues. We overcame these challenges by devising a novel individualized analysis, based on prior theoretical work^20^, that allowed us to detect the first trial after which each animal showed consistent cued approach behavior. This method provided the sensitivity required to establish the precise relationship between experience-induced increases in NAc encoding and the acquisition of cued approach in freely moving animals.

Plasticity mechanisms in the amygdala and dorsal striatum are widely accepted as the likely substrates of fear conditioning^21^ and procedural skill learning^22^, respectively. Surprisingly, it is still unclear whether plasticity in the NAc plays the same crucial role in cued approach learning. While excitatory synapses onto NAc neurons are known to undergo NMDA receptor (NMDAR)-dependent long-term potentiation (LTP)^23–29^, the relevance of this neuroplastic potential in the context of natural learning is poorly understood. We hypothesized that, if NMDAR-dependent NAc plasticity were necessary for learning, NMDAR activation would mediate the growth of cue-evoked excitations in the NAc during training. By blocking NMDARs within the NAc while monitoring the firing rate of NAc neurons, we reveal the dynamic contribution of NMDARs to both NAc activity and cued approach behavior at different stages of learning. Our results identify and characterize a likely physiological substrate for the natural acquisition of cued approach behavior.

## Results

### Cue-evoked behavior debuts at an identifiable trial

**Fig. 1:**
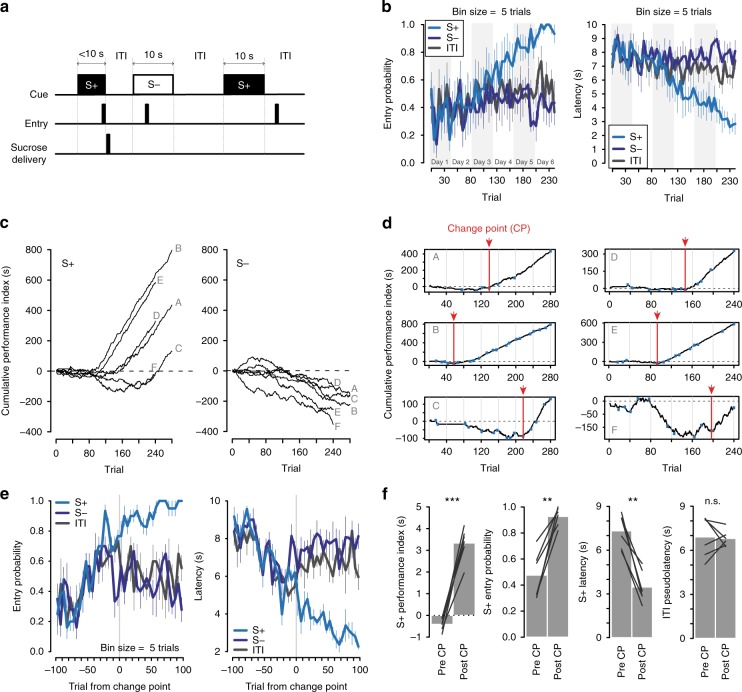
With training, a detectable transition between exploratory and cued behavior occurs. **a** Task diagram. Only receptacle entries during the S+ were rewarded. S+ and S− trials were randomly interleaved. ITI: intertrial interval. **b** Average group entry probability (left) and latency (right) during three task periods: S+ (light blue), S− (dark blue), and the ITI (gray) throughout training in 5-trial bins. Data are expressed as mean ± SEM. **c** Cumulative performance index records on S+ (left) and S− (right) trials (see Methods and Supplementary Fig. 1 for an explanation of this index). Each animal’s behavior is represented by a black line and labeled with a gray letter. **d** Identification of the trial in which the consistent change in behavior took place (see Methods and Supplementary Fig. 2). The black line on each panel is the cumulative record of the performance index of each individual animal. Small blue dots mark the abrupt changes in the slope of the line detected by the algorithm. The vertical red line marks the change point (CP). Vertical gray lines indicate the transition between sessions. **e** Same as in **b**, but with trials aligned to the CP trial. **f** Comparison of average S+ performance index (***p < 0.001, *t* test), entry probability (**p < 0.01, *t* test), latency (**p < 0.01, *t* test) and ITI pseudolatency (*p* = 0.86, *t* test) before vs. after change point; *n.s.* not significant. Details of all statistical tests are in Supplementary Table 1

We recorded the activity of individual NAc core neurons in six naive rats as they learned a cued approach task (Fig. 1a). To earn sucrose reward, animals had to enter a reward receptacle within 10 s of the onset of a discrete, non-localized auditory or visual cue (S+); responses at other times, including during presentation of the unrewarded cue (S−), had no consequences. Each session consisted of 40 S+ and 40 S− trials, randomly interleaved.

During the first 2–3 days of training, animals showed a high rate of receptacle entries during the S+, S−, and the 10 s window at the end of inter-trial intervals (ITIs; Fig. 1b, left). With additional training, the probability of responding to the S+ gradually increased while the latency to respond to the S+ with a receptacle entry decreased (Fig. 1b, right). Although these observations indicate that the animals learned to respond to the S+, the animals continued to respond with high probability during the S− and ITI. Neither S+ response probability nor S+ response latency are sufficient metrics for cued approach performance because neither reflects the difference in responding in the presence vs. the absence of the S+. To calculate this difference, we first determined, for each trial, the ITI pseudolatency, the interval between a point 10 s prior to cue onset and the next receptacle entry. Next, for each trial, we calculated the performance index, the difference between an animal’s ITI pseudolatency and its cue-evoked latency to enter the receptacle on the same trial (latencies were assigned values of 10 s if the animal made no response within 10 s; Supplementary Fig. 1 and Methods). Performance index values range from −10 to 10, with positive values indicating a faster response to the cue than predicted by the rate of responding during the ITI, and negative values indicating the opposite.

To assess the time course of learning for individual subjects, we examined cumulative records of the performance index (Fig. 1c, d). These oscillated around 0 at the beginning of training, indicating that the cue did not yet influence the subject’s approach behavior. Later, at the trial on which faster-than-predicted responses to the S+ were first reliably evoked, a discrete change in the slope of the line can be observed (change point, CP^20^; Supplementary Fig. 2 and Methods). An upward CP occurs in S+ (Fig. 1c, left), but not S− trials (right). Notably, subjects varied in the amount of training necessary to display a CP (Fig. 1c, d). Therefore, the apparent gradual increase in conditioned behavior seen in group data (Fig. 1b) is partly an artifact of averaging asynchronous sudden improvements across subjects.

To characterize the behavioral changes associated with the CP, we examined the mean probability and latency to respond during the S+, S−, and ITI in trials aligned to each animal’s CP (Fig. 1e, f). Before the CP, a steady increase in the probability of entering the reward receptacle was observed during the S+, the S−, and the ITI periods (Fig. 1e, left); animals also showed a decreased latency to respond during each of the periods (Fig. 1e, right). These observations indicate that the rate of responding gradually increased prior to the CP irrespective of the presence of cues. However, at the CP and afterward, there was a clear dissociation between the cue conditions: the probability of S+ entry continued to increase, and the S+ latency decreased. At the same time, the probability and latency of responding in the absence of the S+ stabilized at the CP (Fig. 1e). Mean pre- and post-CP measures confirmed this (Fig. 1f). Thus, the CP represents the trial on which responses to the S+ undergo a sudden and stable increase in probability and vigor, while responses to the S− and to contextual cues (present during the ITI) remain unchanged. Notably, S+ response metrics continued to improve after the CP (Fig. 1e), indicating that learning results in both sudden (CP) and gradual performance increases. Note that while reward delivery in this task was contingent upon the animal’s behavioral response, the learned behavior likely involved a combination of instrumental and Pavlovian components^30^. A separation of these components was beyond the scope of the current work.

### Growth of cue-evoked excitations precedes the CP

**Fig. 2:**
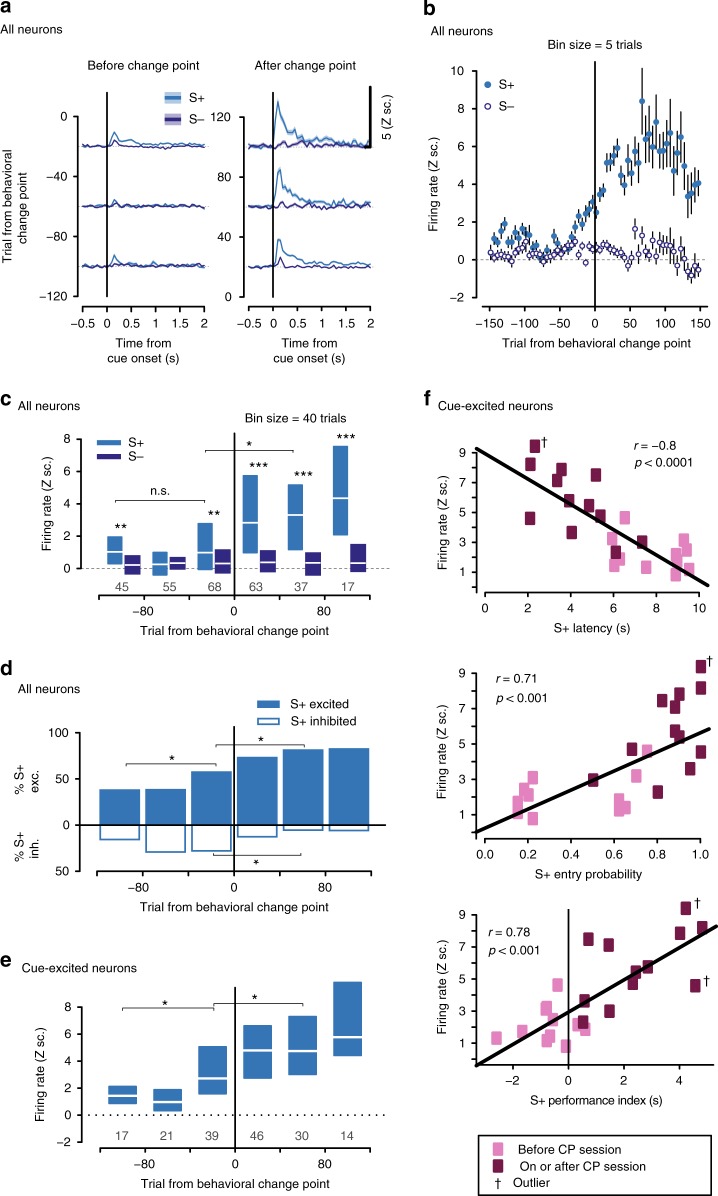
NAc cue-evoked signals increase before CP and escalate with conditioned responding. **a** Firing rate in Z scores (Z sc.) around S+ (light blue) or S− (dark blue) onset, trials aligned to change point (CP). Mean (line) ± SEM (shaded area). **b** Activity (mean ± SEM) 100–400 ms after S+ (light blue) or S− (dark blue) onset around CP (five-trial bins). **c** Same as in **b** but median and interquartile ranges are shown in 40-trial bins. Numbers indicate sample size. Firing after S+ was higher than after S− on most bins. S+-evoked excitations increased after CP (*p < 0.05; **p < 0.01; ***p < 0.001, Wilcoxon). **d** The proportion of cue-excited neurons (solid blue) began to increase before CP. It continued to increase while the proportion of cue-inhibited neurons (white) declined (*p < 0.05, Fisher). **e** Same as in **c** but for cue-excited neurons only. S+-evoked excitations began to increase before CP. **f** Activity in the 100–400 ms post-S+ window strongly correlates with S+ latency (top), S+ entry probability (middle), and S+ performance index (bottom) (***p < 0.001, Pearson). Sessions before/after CP are shown in light/dark purple, respectively

To examine how NAc activity evolved as animals (*n* = 6) learned the task, we recorded from 186 neurons throughout training. Before combining neuronal data from different subjects, we aligned the trials from each neuron to the CP trial in the corresponding subject (Supplementary Fig. 3 for representative neurons). We focused first on cue-evoked excitations because they are prominent in well-trained subjects^2–7^ and play a causal role in cued approach behavior^10,11^. In S+ but not S− trials, large cue-evoked excitations, which peaked at around 100–400 ms after S+ onset, emerged just prior to the CP (Fig. 2a, b). This pattern of emergence of cue-evoked excitations did not depend on the sensory modality of the cue (Supplementary Fig. 4c), or on whether the electrode arrays were advanced between sessions (Supplementary Fig. 6).

Cue-evoked excitations were significantly higher after S+ than after S− onset as early as the first 10–20 trials of training (Supplementary Fig. 4b), indicating that NAc neurons discriminated between the relative reward-predictive value of cues well before the difference in value of the cues was reflected behaviorally, consistent with previous findings^13^. During this stage, cue-evoked firing responses remained small. At a later stage, during the trials leading up to the CP, cue-evoked excitations progressively increased in magnitude (Fig. 2a–c, e) and prevalence (Fig. 2d). To address how the growth in cue-evoked excitations relates to the debut of vigorous cued approach behavior, NAc activations during the bin of 40 trials that preceded the CP were compared to an earlier and later trial bin (Methods: Statistical analysis). Both the proportion of significantly cue-excited neurons (Fig. 2d) and their excitation magnitude (Fig. 2e) began to increase just prior to the CP. After the CP, as reward-seeking behavior became more vigorous, the prevalence (Fig. 2d) and magnitude (Fig. 2e) of cue-evoked excitations continued to increase. Consistent with observations in well-trained animals^8,10,11,31^, the magnitude of cue-evoked excitation was strongly correlated with the latency and probability of the cued approach (Fig. 2f), suggesting that S+-evoked firing promotes approach. (The proportion of cue-inhibited neurons significantly decreased after the CP; however, because these represent a small proportion of units—18%—our analyses focus on excitations).

**Fig. 3:**
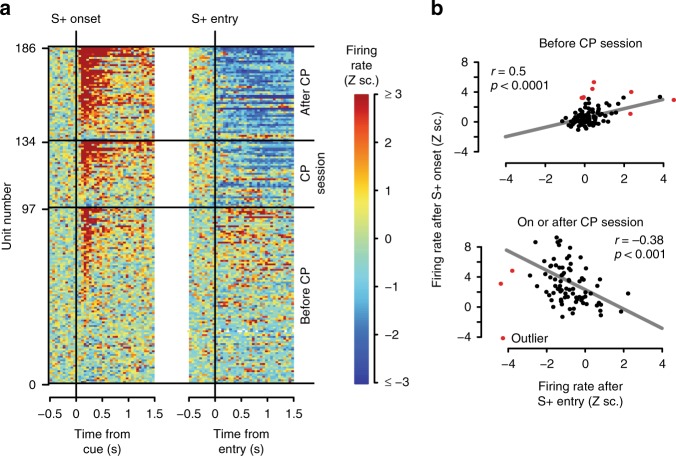
After CP, NAc responses during rewarded entries switch from excitations to inhibitions. **a** Colors indicate average activity around S+ (left) or S+ entry (right) in 50 ms bins, in blocks before, during, or after change point (CP) session. Z sc.: Z scores. Neurons are sorted within each block based on the magnitude of their post-S+ response. **b** Firing elicited by S+ onset and S+ entry is strongly correlated. The relationship changes from positive before CP (top) to negative after CP (bottom)

In reinforcement learning, reward is typically experienced some time after the reward-predictive cue and reward-eliciting actions occur, thus posing a theoretical conundrum known as the credit assignment problem^32^: how do neural representations that deserve credit for predicting the reward become selectively strengthened? One potential mechanism is that cue-excited neurons remain excited during subsequent reward delivery, providing an eligibility trace that facilitates further strengthening of cue-evoked excitation. Supporting this possibility, we established that the firing of cue-excited neurons just prior to and after the CP remained elevated during the 750–2000 ms post-S+ window, long after their initial peak (Supplementary Fig. 4d). Firing during the 2 s window prior to long-latency (≥5 s) entries was also higher during S+ presentation than S− presentation (Supplementary Fig. 4e). Finally, we examined whether an additional excitation occurred during reward delivery by aligning firing to reward receptacle entry. Before the CP, some NAc neurons exhibited excitations within 1 s after rewarded entries occurring during S+ presentation (Fig. 3a). Strikingly, there was a significant positive correlation between S+-evoked and entry-evoked activity at this point in training, suggesting that neurons that became cue-excited early in training also tended to exhibit elevated activity upon reward delivery (Fig. 3b). In contrast, activity triggered by the S+ was not correlated with activity after unrewarded receptacle entries (Supplementary Fig. 5b). Together, these results suggest that NAc neuronal firing bridges the gap between cue and reward, potentially serving as a substrate for plasticity.

In the CP session and afterward, receptacle entries during the S+ were followed by pronounced inhibitions (Fig. 3a). The emergence of entry-related inhibitions was absent in non-rewarded entries (Supplementary Fig. 5c). Also, cue- and reward-elicited activity became negatively correlated (units that were more excited upon S+ onset became more inhibited at the time of reward consumption; Fig. 3a, b), consistent with previous observations in trained animals^5,33^. No such correlation was observed between post-S+ activity and that seen following non-reward receptacle entries (Supplementary Fig. 5c). Thus, not only S+-evoked firing but also reward-associated firing changes during learning.

### NAc NMDARs are required for both performance and learning

If the co-temporal emergence of cue-evoked excitations and cued approach means that the changes in cue-evoked firing are causal to learning, then preventing the acquisition of cue-evoked excitation during training should also prevent acquisition of cued approach. Given that NMDARs are necessary for both LTP of excitatory synapses in the NAc^23,25–27,34^ and acquisition of reward-conditioned behaviors^17–19^, we predicted that bilateral injections of an NMDAR antagonist into the NAc core prior to daily training sessions would prevent both the emergence of cue-evoked excitations and cued approach learning. A preliminary study confirmed these predictions (Supplementary Fig. 7), but the interpretation is problematic. NMDAR blockade might decrease entries into the reward receptacle^35^, reducing cue-reward learning as a secondary consequence of a performance deficit. Therefore, we first performed an experiment to gain a clearer understanding of how, during different learning stages, intra-NAc NMDAR blockade affects both cued approach performance and NAc cue-evoked firing.

**Fig. 4:**
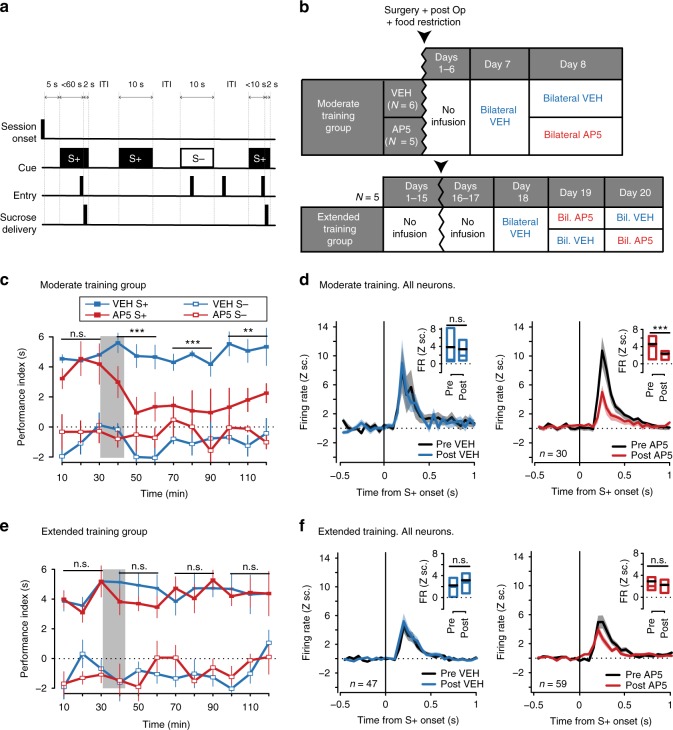
NMDARs transiently mediate the expression of cued NAc activity and approach behavior. **a** Task. ITI: intertrial interval. **b** Microinjection schedule. **c** For the moderate training group, performance index in S+ (solid) and S− trials (empty squares) before and after saline (VEH, blue) or AP5 (red) microinjection (mean ± SEM). The gray rectangle represents the microinjection period. In S+ trials, AP5-treated subjects performed worse than subjects in the control group in every post-injection 30 min bin (***p < 0.001; **p < 0.01; *t* test). **d** Firing rate (FR; mean ± SEM) in Z scores (Z sc.) around S+ onset in the moderate training group before (gray) or after injection of saline (blue, left) or AP5 (red, right). Insets show activity 100–400 ms after S+ before (pre) or after (post) injection. Boxes show the interquartile range (box height), mean (black line), and median (red/blue line). Activity was unaffected by saline (*p* = 0.64), but reduced by AP5 injections (***p < 0.001, Wilcoxon). **e**, **f** Same as in **c**, **d**, but for animals in the extended training group. In this group, performance was not disrupted by AP5 injections (*p* > 0.05, *t* test). S+-evoked activity was also unaffected by saline (*p* = 1) or AP5 injections (*p* = 0.2, Wilcoxon)

Animals were trained in a simple cued approach paradigm (Fig. 4a) for either seven (moderate training) or 18 (extended training) days. After training, they underwent a test session during which they received either d-(−)-2-amino-5-phosphonopentanoic acid (AP5) or vehicle microinjections targeted to the NAc core, while the activity of NAc neurons was monitored using electrophysiological recordings (Fig. 4b). Moderate and extended training groups showed similar S+ vs. S− discrimination prior to the test session (Fig. 4c, e, pre-injection period). In the moderate training group, AP5 infusions significantly reduced S+ performance index (Fig. 4c) and S+ entry probability, while increasing S+ latency and ITI pseudolatency (Supplementary Fig. 8a–c). These behavioral changes were accompanied by a decrease in cue-evoked excitations in NAc core neurons (Fig. 4d) with no change in baseline firing rates (Supplementary Fig. 8g, h). Strikingly, in the extended training group, cued approach (Fig. 4e; Supplementary Fig. 8d–f) and cue-evoked excitations (Fig. 4f) were unaffected by AP5, and the magnitude of cue-evoked excitations in the absence of the drug was much smaller than that seen in the moderate training group (Fig. 4d, f). These results indicate that after asymptotic performance is attained, further training leads to a new stage of learning. This stage is characterized by a decline in the contribution of NAc NMDARs to both NAc cue-evoked excitation and cued approach behavior.

**Fig. 5:**
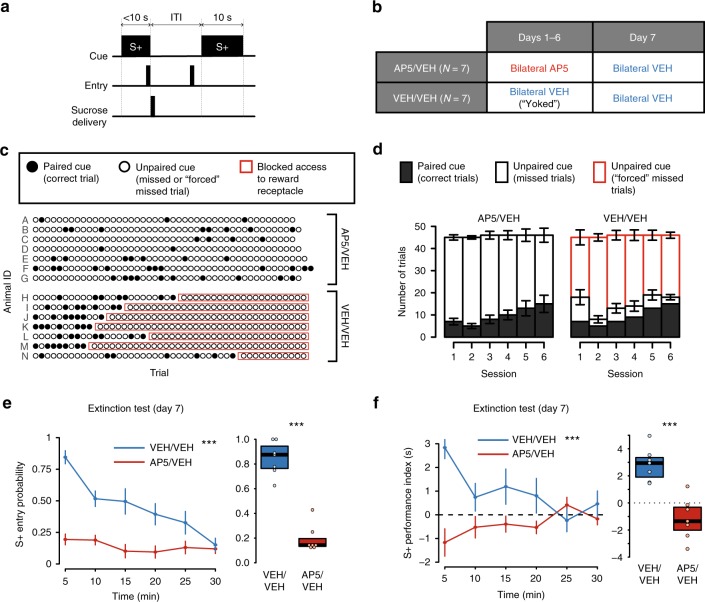
NMDARs in NAc are necessary for the acquisition of cued approach behavior. **a** Task. ITI: intertrial interval. **b** Microinjection schedule. VEH: vehicle. **c** “Yoking” procedure (sample session). Each AP5/VEH or VEH/VEH subject is identified by a letter. Bubbles indicate trials during which subjects made a cued entry (black) or not (white). Red boxes indicate trials when the receptacle was closed. **d** Mean ± SEM number of “Paired” (black) and “unpaired cue” trials (white) experienced on each session by the AP5/VEH or VEH/VEH group. Red bars indicate trials during which the receptacle was closed. Both groups experienced the same number of “paired” and “unpaired cue” trials daily. **e** Mean ± SEM S+ entry probability in the VEH/VEH (blue) and AP5/VEH (red) groups on the test (drug-free) session (whole session, left; first 5 min, right). No rewards were delivered. There were main effects of the drug, bin and drug × bin interaction (Supplementary Table 1). Box plots show median, interquartile range, and individual values. S+ entry probability was lower in previously AP5-treated subjects (***p < 0.001, *t* test). **f** Same as in **e** but for S+ performance index. There were main effects of drug and drug × bin interaction. During the first 5 min, animals that received AP5 injections during training exhibited lower S+ performance index (***p < 0.001; *t* test)

The strong effect of intra-NAc NMDAR blockade on the expression of cued approach behavior (and on cue-evoked excitations) in moderately trained animals suggests that NAc NMDARs contribute to normal behavioral performance during training trials. To assess whether NAc NMDARs contribute to learning beyond their role in performance, we asked whether intra-accumbens NMDAR blockade impairs learning to a greater extent than the introduction of an artificial performance deficit of equivalent magnitude to that caused by NMDAR blockade. New groups of animals received daily bilateral microinjections of either AP5 (AP5/VEH) or saline (VEH/VEH) prior to each training session. After 6 days of training, all animals underwent a drug-free extinction test (Fig. 5a, b). During training, both groups were matched (yoked) to ensure that the VEH/VEH and AP5/VEH groups received the same number of cue-reward pairings (Fig. 5c, d). The VEH/VEH group was prevented from experiencing more cue-reward pairings than the AP5/VEH group by blocking access to the reward receptacle.

In the drug-free extinction test, despite exposure to a comparable number of cue-reward pairings during training, animals that had been treated with AP5 during training showed a reduced likelihood of entering the receptacle during the S+ than control animals (Fig. 5e). Importantly, animals in the VEH/VEH group also exhibited a significantly higher S+ performance index than those in the AP5/VEH group (Fig. 5f), indicating that the control group learned to respond specifically to the S+ rather than generally increasing its responding irrespective of cue presentation.

Together, these results demonstrate that activation of NMDARs in the NAc core during training is transiently necessary for normal expression of cued approach behavior and plays a critical role in the acquisition of the conditioned approach response over and above its effects on behavioral performance.

### NAc plasticity underlies the emergence of cue-evoked signals

Because bilateral AP5 injection blocks not only acquisition of cued approach behavior (Fig. 5) but also its expression (Fig. 4), the impact of the antagonist on NAc unit activity during early cued approach training is likely to reflect some combination of learning and performance deficits. In previous studies^10,11^, we found that unilateral injections of performance-impairing agents into the NAc only minimally disrupt performance. We therefore examined NAc activity in rats microinjected with AP5 unilaterally prior to each daily training session, allowing us to compare learning-related activity in the NAc in AP5- vs. vehicle-infused hemispheres within individual animals.

**Fig. 6:**
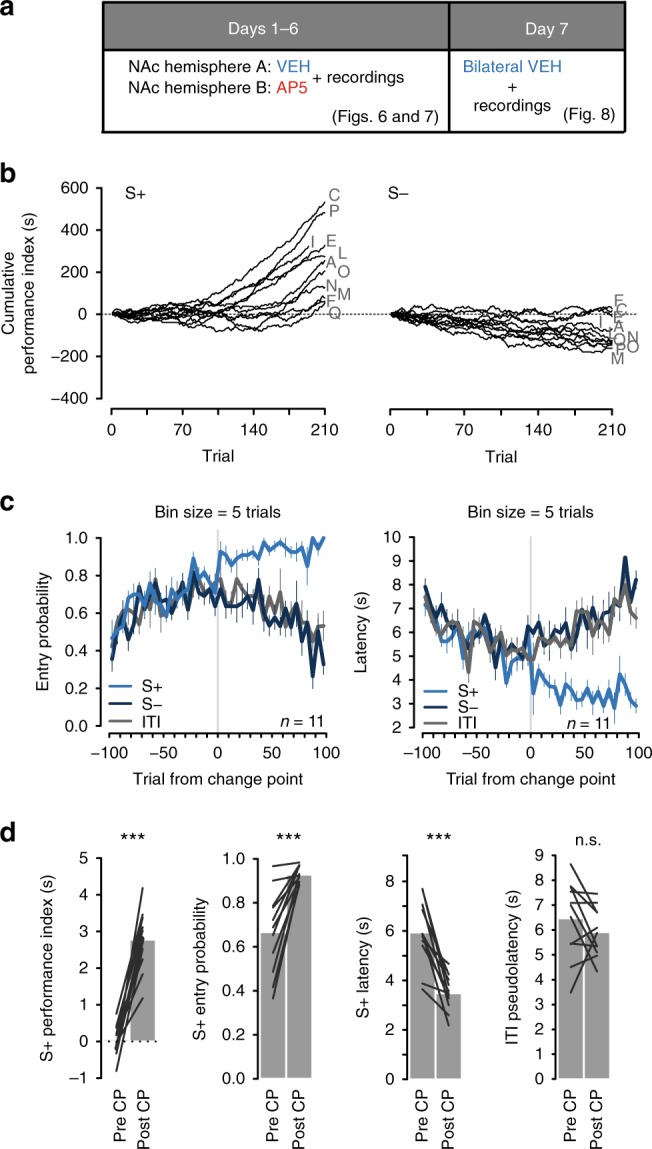
Unilateral blockade of NMDARs in NAc core did not abolish learning in most subjects. **a** Microinjection schedule. VEH: vehicle. **b** Cumulative performance index records on S+ (left) and S− (right) trials in 11 animals that, despite receiving unilateral intra-accumbens AP5 injections during training, were able to acquire cued approach behavior. Each subject’s behavior is represented by a black line. Letters identify different subjects. **c** Left: Mean ± SEM entry probability during the S+ (light blue), S− (dark blue), or pre-cue 10 s ITI window (gray) with respect to the trial in which the change point occurred. Before change point, the overall rate of receptacle entry shows a steady increase until, at the change point, the rate of cued entry continues to increase while the rate of uncued entry stabilizes. Right: same as left panel but for latency and ITI pseudolatency. **d** In subjects that received unilateral AP5 injections prior to each training session, S+ performance index and S+ entry probability increased after change point (***p < 0.001; *t* test). S+ latency declined (***p < 0.001; *t* test), while the ITI pseudolatency did not change after change point (*p* = 0.241; *t* test); *n.s.* not significant

Animals were given daily AP5 microinjections into the same hemisphere prior to each training session; vehicle was injected contralaterally (Fig. 6a). Of 17 animals, 11 acquired cued approach behavior within 6 days of training as evidenced by the detection of a behavioral CP for S+, but not S− trials (Fig. 6b). The S+ performance index and S+ entry probability were significantly higher after the CP than before, whereas S+ latency was significantly shorter (Fig. 6d). Furthermore, the average performance aligned to the CP trial (Fig. 6c, d) was comparable to that of uninjected animals (Fig. 1e). The remaining six rats were classified as non-learners based on the absence of a detectable CP (Supplementary Fig. 11).

**Fig. 7:**
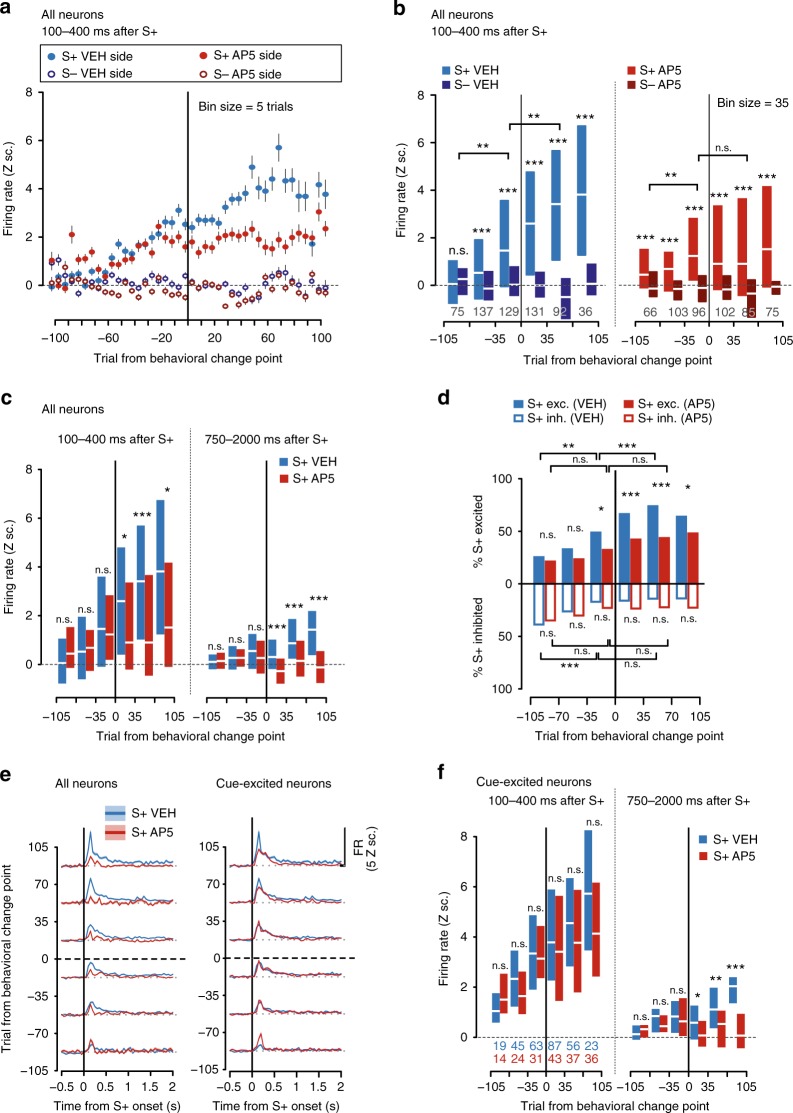
AP5 prevented large and prolonged NAc cued excitations from emerging with training. **a** Mean ± SEM excitation 100–400 ms after S+ (solid) or S− (empty) in the saline (VEH, blue) or AP5 (red) hemispheres in five-trial bins around CP. Z sc.: Z scores. **b** Same as in **a**, but median and interquartile range are shown in 35-trial bins. Activity was higher after S+ than S− onset at least 70 trials before CP in both hemispheres (***p < 0.001, Wilcoxon). After CP, S+-evoked activity increased in the vehicle (**p < 0.01), but not the AP5-treated side (*p* > 0.05, Wilcoxon). Numbers indicate sample size. **c** Comparison of post-S+ activity across hemispheres. After CP, population activity was higher in the vehicle than in the AP5-treated side in the 100–400 and 750–2000 ms post-S+ windows (*p < 0.05; ***p < 0.001, Wilcoxon). **d** Proportion of excited (top) or inhibited (bottom) units after S+ onset around the CP. The proportion of S+-excited units was higher in the vehicle (blue) than in the AP5 side (red) right before CP and remained higher thereafter (*p < 0.05; ***p < 0.001, Fisher). **e** Mean ± SEM activity around the S+ of all (left) or cue-excited (right) units in the vehicle (blue) or AP5 (red) sides. **f** Same as in **c**, but for cue-excited neurons (*p < 0.05; **p < 0.01; ***p < 0.001, Wilcoxon); *n.s.* not signifcant

**Fig. 8:**
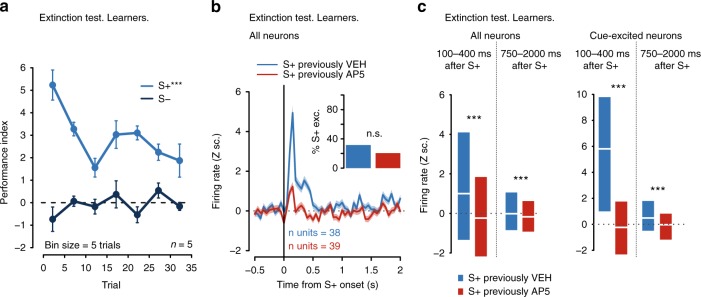
The effects of AP5 on NAc firing outlast the presence of the drug in the brain. Data from drug-free extinction sessions of rats that received unilateral AP5 infusions during training and learned the task (Learners). VEH: vehicle. **a** Mean ± SEM performance on S+ (light blue) and S− (dark blue) trials. Main effects of cue and cue × bin interaction (Supplementary Table 1). **b** Mean ± SEM firing activity in Z scores (Z sc.) around S+ onset in NAc neurons treated with saline (blue) or AP5 (red) during training. Inset: proportion of neurons that were classified as cue-excited in the side previously treated with saline (blue) or AP5 (red) (*p* = 0.31, Fisher). **c** Activity (median and interquartile range) 100–400 ms or 750–2000 ms after S+ onset in the NAc that had been treated with saline (blue) or AP5 (red) during training. All neurons (left) vs. cue-excited neurons (right)

In the 11 learners, interhemispheric differences in the emergence of cue-evoked excitations cannot be explained by a performance deficit. Therefore, we compared the emergence of cue-evoked excitations in the AP5-injected vs. vehicle-injected NAc during training (Fig. 7; *n* = 313 and 273 neurons from vehicle and AP5 hemispheres, respectively; firing data from non-learners is shown in Supplementary Fig. 11). Later, we conducted an extinction test to assess the persistence of any AP5-induced deficit (Fig. 8).

**Fig. 9:**
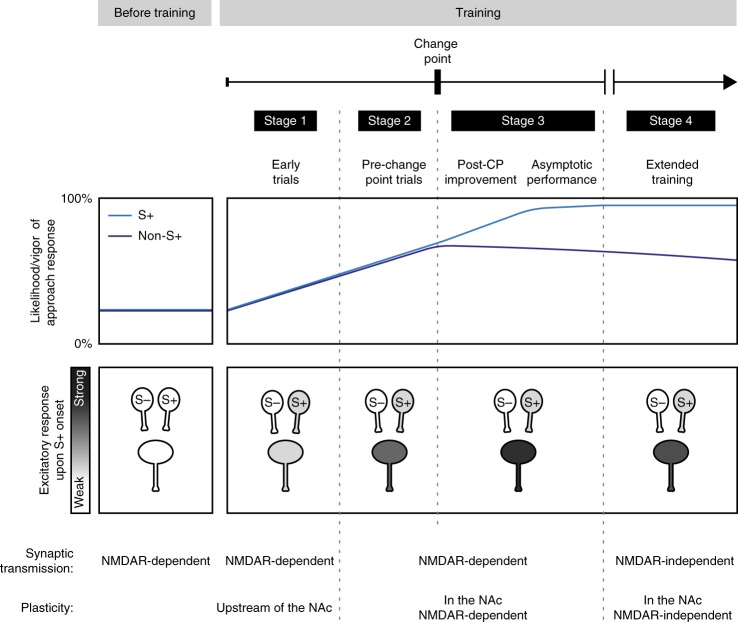
Model of the stages of learning cued approach behavior and underlying NAc mechanisms. Top box: likelihood/vigor of approach responses prior to training (left box) and at different points of training (right box) in the presence (light blue) or absence (dark blue) of the S+. Bottom box: conceptual diagrams depicting, at different stages of learning, changes in the strength of S+-evoked excitatory responses in NAc neurons as well as NAc afferents that encode either the S− (left) or the S+ (right). Early in training (stage 1), small S+-evoked excitations appear and are unaffected by AP5, likely implicating plasticity elsewhere in the circuit in which the NAc is embedded (likely upstream of the NAc). Just before CP and after CP (stages 2 and 3), NMDAR-mediated plasticity within the NAc is required for the growth of NAc cue-evoked excitations. Between the trials before CP and the point at which animals are showing asymptotic performance, expression of S+-evoked excitations in the NAc requires NMDAR-mediated excitatory transmission, but it becomes independent from it with extended training (stage 4)

During training, S+-evoked excitations were significantly larger than those elicited by the S− in both AP5- and saline-injected hemispheres (statistical comparisons across bins and conditions followed the same rationale as in Fig. 2). This dissociation emerged many trials before the CP (Fig. 7a, b), suggesting that AP5 did not prevent NAc core neurons from encoding the reward-predictive value of the cue in the earliest stage of training (stage 1, Fig. 9). However, just prior to the CP (stage 2, Fig. 9), the proportion of neurons classified as S+-excited became significantly larger in the vehicle side than the AP5 side, and this proportion remained larger in all trial bins until the end of training (Fig. 7d). The proportion of S+-inhibited neurons diminished before the CP in the vehicle but not in the AP5 side, and it did not significantly decrease after that in either hemisphere (Fig. 7d). Even though AP5-treated neurons were less likely to become cue-excited, when they did, their firing rate in the 100–400 ms window was lower than the firing rate of neurons in the vehicle side—but not significantly so (Fig. 7f, left). However, after the CP (during stage 3, Fig. 9), cue-excited neurons in the AP5-treated side were significantly less excited in the 750–2000 ms window after S+ onset than the corresponding neurons in the vehicle side (Fig. 7f right). Thus, in the absence of behavioral effects of AP5, the drug disrupted the increase in S+-evoked excitations occurring just prior to and after the CP.

Neurons in the vehicle-infused side showed growth of S+-evoked excitations after the CP (stage 3, Fig. 9), paralleling the increasing probability and decreasing latency of the S+-elicited approach response. While neurons in the AP5-treated hemisphere of the same animals showed S+-evoked excitations before the CP (stages 1 and 2, Fig. 9), the magnitude of these excitations did not grow after the CP (Fig. 7a, b; stage 3, Fig. 9). Direct comparison between hemispheres confirmed that significant differences in the magnitude of S+-evoked activity caused by AP5 appeared after the CP (Fig. 7c). This was true for the post-S+ 100–400 ms window as well as for the 750–2000 ms window, indicating that neurons in the vehicle- but not AP5-treated side remained excited for an extended period after presentation of the reward-predictive cue (Fig. 7c, e, left panel). Thus, NMDARs are required for both the growth in peak S+-evoked excitation and for the extended tail of these excitations.

Baseline firing rates did not differ across vehicle- and AP5-infused hemispheres (Supplementary Fig. 9), nor did the hemispheres differ in terms of firing rates during non-reward periods (S− or ITI; Supplementary Fig. 10a) or after receptacle entries following the S+ (Supplementary Fig. 10c). In summary, blockade of NMDARs in the NAc core during training disrupted the increase in S+-evoked excitations that normally accompanies increases in the vigor of the cued approach without affecting neuronal firing during other periods of the task.

Because intra-accumbens blockade of NMDARs disrupts the expression of cue-evoked excitations (Fig. 4), it is possible that AP5 simply reduced ongoing excitatory transmission without impairing learning-related plasticity. To test whether the drug had long-lasting effects on NAc cue-evoked excitations, we conducted a drug-free test session in a subset (*n* = 7) of animals and compared the magnitude of cue-evoked excitations in the previously AP5-treated hemisphere with those in the previously saline-treated hemisphere. Saline was infused into both hemispheres just prior to the test session and, to prevent cue-reward learning, no rewards were delivered.

Two of the seven rats were non-learners during training (Supplementary Fig. 11). The remaining five rats (learners) showed significantly higher S+ than S− performance in the drug-free extinction test (Fig. 8a). Critically, neurons in the hemisphere that had been treated with saline infusions during training showed significantly higher firing rates in response to the S+ compared to neurons in the formerly AP5-treated hemisphere. These intra-hemispheric differences in S+-evoked activity were observed both during the 100–400 and the 750–2000 ms windows after cue onset (Fig. 8b, c). This effect was not due to differences in baseline firing rate across hemispheres (Supplementary Fig. 9). Therefore, the reduction in cue-evoked excitations produced by AP5 during training outlasted the presence of the drug in the brain. Together, these observations indicate that NMDARs in the NAc are required for the long-lasting plasticity that causes the growth of large-magnitude cue-evoked excitation in NAc neurons. In turn, the NMDAR-mediated emergence of cue-evoked NAc responses drives the development of vigorous cued approach during learning.

## Discussion

Learning to expect and seek natural rewards in the presence of predictive environmental cues is essential for survival. NAc neurons receive massive convergent cue- and reward-related information, and project to motor output regions^36^. Excitatory synapses in the NAc can undergo LTP^23–28^, and in vivo studies have implicated the NAc in the acquisition of reward-reinforced behavior^14–19^. Because of these findings, it is often assumed that plasticity in the NAc is causally linked to learning of cued reward-seeking behavior. Indeed, addictive behavior is often regarded as a pathological form of cue-conditioned behavior that is acquired because drugs of abuse usurp plasticity mechanisms in the NAc^37^. Yet, the specific NAc-dependent mechanism underlying natural cue-reward learning has not been identified. Here, we provide insight into this mechanism by demonstrating that the NAc cue-evoked neuronal firing response that drives cued approach to reward develops prior to expression of the learned behavior, and that this process requires NMDAR-dependent plasticity in the NAc. Moreover, plasticity in the BLA-NAc synapse has been associated with appetitive learning^38,39^.

Our experiments reveal that cued approach task acquisition occurs in distinct stages (Fig. 9). In stage 1, small S+-evoked excitations differentiate the S+ from the S− from the first training trials, well before learning is manifested behaviorally (Supplementary Fig. 4b). Intriguingly, these excitations were unaffected by intra-accumbens NMDAR blockade (Fig. 7b), suggesting that the plasticity necessary for these responses takes place upstream of the NAc (Fig. 9). One candidate is the basolateral amygdala (BLA), the activity of which is essential for NAc cue-evoked excitations^40^ and which contains neurons that encode the motivational value of stimuli after just a few trials^41–43^ via NMDAR-mediated plasticity^44^.

During stage 2, S+-evoked excitations in NAc neurons begin to grow a few trials before the CP. This growth continues in stage 3 as S+ responding becomes more reliable and vigorous (Fig. 9), corresponding with increasing vigor of the cued approach (Fig. 2f). Disruption of these large cue-evoked excitations was associated with a reduction in cued approach behavior (Fig. 4c, d), consistent with previous observations^10,11,40,45,46^. These findings indicate that the growth of S+-evoked excitations propelled the increase in the probability and vigor of cued approach behavior.

Daily bilateral NMDAR antagonist injections into the NAc prevented the emergence of both cued approach learning and the learning-related NAc signals (Supplementary Fig. 7). It is likely that these suppressive effects were due, at least partly, to a contribution of NAc NMDARs to the expression of learned behavior (and not merely to its acquisition), as the antagonist reduced both cued approach performance and the cue-evoked excitations of NAc neurons even in animals that had been trained to distinguish the S+ from S− prior to drug infusions (Fig. 4c, d). However, the dependence of S+-evoked excitation and behavioral performance on NMDARs was absent in overtrained animals (Fig. 4), suggesting that overtraining induces a secondary plastic change within the NAc core^47^ (stage 4, Fig. 9). A simple explanation of this shift to NMDAR-independent behavioral and neuronal activity would be an increase in the ratio of AMPA receptors to NMDARs such that NMDARs contribute very little to cue-evoked NAc excitation during this stage. Therefore, although previous studies have used the resistance of overtrained cued approach performance to NAc NMDAR blockade as evidence that the antagonist acts specifically to inhibit learning during task acquisition^17–19^, our results indicate that performance is, in fact, dependent on NMDARs during learning stages prior to stage 4.

The presence of an antagonist-induced performance deficit does not, however, rule out an additional effect on learning. A performance deficit during early training would likely disrupt learning by limiting the number of paired encounters with the cue and reward. However, we found that antagonist-injected animals exhibited severe learning deficits compared with yoked controls that received no drug but an equivalent number of cue-reward pairings (Fig. 5). Although the antagonist may have disrupted other performance-related factors, such as the timing of rewards within the session or the interval between cue onset and reward, the most parsimonious interpretation is that daily NMDAR antagonist treatment induced both a performance deficit and an additional learning deficit.

To isolate the contribution of NAc NMDARs to learning and the emergence of cue-evoked NAc excitations, we gave daily NAc injections of the NMDAR antagonist unilaterally, which results in a normal time course of learning in most animals (Fig. 6). Simultaneously, we recorded NAc neuronal activity in both antagonist- and vehicle-injected hemispheres. NMDAR blockade prevented the growth of cue-evoked excitations that would have otherwise started near the CP (Fig. 7a–f). Because reduced cue-evoked firing in the drug-injected hemisphere persisted even in the absence of the drug (Fig. 8b, c), we conclude that NAc NMDAR transmission mediates the plasticity necessary for NAc neurons to undergo the experience-dependent physiological changes that give rise to cued approach behavior.

It is possible that the antagonist-induced disruption of excitatory transmission in the NAc interfered with plasticity in downstream structures. Because the NAc is embedded in a recurrent corticostriatothalamocortical loop, intra-accumbens AP5 injections that diminish cue-evoked excitations could have reduced excitatory drive onto neurons that are both downstream and upstream of the NAc (e.g., in the thalamus or prefrontal cortex). Consequently, NAc AP5 injections could have impaired plasticity in these structures, but not within the NAc itself, resulting in diminished cue-evoked excitations even when AP5 is no longer present. Indeed, plasticity within other corticostriatothalamic structures may well have contributed to learning, as shown by the ability of NAc neurons to differentiate the S+ from the S− very early in training in a NAc NMDAR-independent way (Fig. 7b, e). However, given the well-established role of NAc NMDARs in LTP in the NAc^23–29^, it is unlikely that our AP5 injections, which reduced the magnitude of NAc neurons’ cue-evoked excitations, impaired plasticity only elsewhere and not in the NAc. Therefore, the most likely explanation for our results is that AP5 disrupted learning by preventing synaptic potentiation at the level of the NAc.

In fact, NMDARs could contribute to the plasticity underlying the increase in NAc cue-evoked excitations in at least three ways. First, the observation that AP5 reduced the magnitude of peak cue-evoked excitation in previously trained animals (Fig. 4) is consistent with a role for NMDARs in basal synaptic transmission^48^. Because strong excitation is a prerequisite for associative plasticity^49,50^, NMDARs could contribute to plasticity simply by facilitating excitation upon S+ presentation. Second, NMDARs could contribute the postsynaptic Ca^2+^ elevation required for some forms of LTP, consistent with observations of NMDAR-dependent LTP in NAc neurons^23–28^. Finally, because cue-evoked excitations in NAc core neurons were of remarkably long duration (at least 2 s, Fig. 2a; Supplementary Fig. 4d, e) and were considerably shortened by NMDAR blockade (Fig. 7c, e, f), NMDARs could contribute to plasticity by facilitating this prolonged excitation. Specifically, the prolonged time course of cue-evoked excitation could result from the slow kinetics of NMDAR-mediated transmission^51,52^, which facilitate robust temporal summation and increase spike output upon repeated stimulation^53^. Early in training, a mesolimbic dopamine signal at the time of reward delivery encodes a value prediction error^54^ that is thought to contribute to striatal plasticity^55–57^, but only when dopamine release onto NAc neurons occurs within 2 s of glutamatergic input^29,58,59^. By keeping NAc neurons in a state of elevated activity, NMDARs could allow the same neurons that were excited by the cue to maintain their excitation until the time of reward delivery. By this mechanism, NAc neurons could provide a neural eligibility trace. Alternatively, the observation that, in stages 2 and 3, neurons exhibiting cue-evoked excitations also tend to be excited during reward delivery (Fig. 3) suggests that the afferents carrying the excitatory reward signal could facilitate the potentiation of cue-evoked excitations by providing additional excitation during their long tail of elevated firing.

It remains unclear which sources of synaptic input are strengthened as animals learn to approach the receptacle in response to reward-paired cues. High-frequency stimulation of the BLA, prefrontal cortex, and hippocampus each leads to NMDAR-dependent potentiation of their excitatory synapses^23,26,27,34^. In trained animals, neurons in all of these regions exhibit cue-evoked excitations^41,42,60,61^, and intact input from the BLA and prefrontal cortex is required for cue-evoked excitations in NAc core neurons as well as for the performance of cued approach behavior^40,46^. Further research to characterize the role of the NAc’s afferents in the development of cue-evoked excitations is necessary to better understand how these excitations emerge and drive cued approach learning.

In summary, NAc neuronal activity recorded across acquisition of a cued approach task revealed that learning occurs in identifiable stages (Fig. 9). In stage 1, before the CP, some NAc neurons exhibited small firing responses to cues and even differentiated between S+ and S−. These early NAc AP5-insensitive responses were likely the result of plasticity in upstream structures. Similarly, in overtrained animals (stage 4), the expression of S+-evoked excitation became independent of NAc NMDARs. In between (stages 2 and 3), beginning prior to the CP, many NAc neurons escalated their S+-evoked excitation, causing increasingly vigorous S+-directed approach responding. Our results demonstrate that NMDARs within the NAc are required for both the expression and plastic growth of these excitations and that these contributions underlie cued approach learning. Thus, we identify a fundamental plasticity mechanism that brings animals closer to reward.

## Methods

### Animals

Male Long–Evans rats (*N* = 58; 350–375 g; Charles River, NY) were used in this study. Upon arrival, they were placed on a 12 h light/12 h dark schedule and housed in groups of two or three. They were handled regularly for at least 1 week before surgery (or training if that came first). Following surgery, animals were singly housed, received postsurgical care as necessary and were allowed to recover for at least 1 week. After recovery, they were placed on a restricted diet of 13 g of standard chow. Behavioral training started after animals had been food deprived for a week and the restricted diet was maintained for the remainder of the experiment. Animal care was in accordance with the US National Institute of Health “Guide for the Care and Use of Laboratory Animals” and approved by the Institutional Animal Care and Use Committee at Albert Einstein College of Medicine.

### Operant chambers

Custom-made Plexiglass operant chambers (40 cm square base and 60 cm tall) were used in this study. Chambers were controlled using Med-PC software. On one of the walls of each chamber there was a reward receptacle equipped with an infrared beam for detection of head entries and exits. Chambers were also equipped with 28 V house lights, a white noise generator, and speakers. A syringe pump adjacent to the chamber delivered the reward into a well inside the receptacle via steel-reinforced PVC tubing. All chambers were kept inside soundproof cabinets and 65 dB white noise was played throughout each session to minimize acoustic interference. Timestamps associated with task-related events were recorded with a resolution of 1 ms.

### Training protocols

- *Pretraining phase*: After a week of food restriction and regular handling, animals were habituated to 10% sucrose in their home cage for at least 2 h. After that, they received a mock infusion in which two injectors were bilaterally inserted and remained in place for ~ 30 s. In electrophysiological recording experiments, rats were also habituated to the experience of being tethered to the cable before training. This was carried out in a plastic container of similar dimensions to the operant chamber. One end of a recording cable was connected to a commutator above the container, and the other end was attached to the rat’s headstage for at least 30 min. Before training, all rats also received a receptacle training session. During the receptacle training session, animals received 40 rewards upon receptacle entry on a fixed-ratio 1 (FR1) schedule. Delivery of the first 20 rewards was followed by a 10–15 s time out and delivery of the last 20 rewards was followed by a 30–45 s time out. If animals failed to collect all the rewards within ~45 min, they were given a second receptacle training session the next day. On this second session, the receptacle was baited with sucrose before putting the animal inside the chamber. Animals in the yoking experiment (Fig. 5) received receptacle training in an alternative context that consisted of a standard 30 × 25 cm^2^ operant chambers with a grid floor and dark metallic walls (Med Associates; St. Albans, VT). During this session, receptacle entries were rewarded on an FR1 schedule with a 10 s post-reinforcement time out until 20 rewards were delivered. After receptacle training, animals in this experiment were also given two 30 min sessions in the chambers that would be used for subsequent training. During these sessions, the white noise generator and the house lights were on but no cues or rewards were presented. The goal of these sessions was to extinguish contextual cues and thereby promote acquisition of the cue /reward association^62^.
- *Training phase*. Three different versions of the cued approach task were used.
  i. *An S+ signals reward availability (Fig.* *5a**)*: Each trial consisted of an ITI of 30 s on average (exponentially distributed; min = 15 s, max = 45 s) and an auditory cue (S+) that consisted of a siren alternating between 4 and 8 kHz. A head entry into the reward receptacle during presentation of the cue resulted in delivery of a droplet (~150 μl) of 10% sucrose into the same compartment (correct trial) and offset of the cue. If the animal failed to enter the compartment during presentation of the cue (missed trial), the tone would terminate after 10 s and sucrose would not be delivered. Entries into the compartment during the ITI had no programmed consequences. Each daily session ended after 30 min. This version of the task was used in the yoked control experiment (Fig. 5). No other cues were included in this version of the task because doing so would make it very hard to yoke the experience across groups.
  ii. *An S+ and S− signal whether or not reward is available (Fig.* *1a**)*: Evaluating whether cue-evoked neural responses encode the reward-predictive value of the S+ (as opposed, for example, to its saliency) requires the addition of a control non-reward-predictive cue (S−). For that reason, all recording experiments included a 10 s S− in addition to the S+. Entries into the reward compartment during the S− had no programmed consequences. Both cues consisted of non-localized stimuli of different sensory modalities to facilitate discrimination. One cue was an auditory stimulus (a siren alternating between 4–8 kHz) and the other one was a visual stimulus (four house lights turning on in an otherwise dark chamber). Assignment of sensory modality to cue type (S+ or S−) was counterbalanced across subjects. Each daily session consisted of 80 trials: 40 S+ and 40 S− trials. Both kinds of trials were randomly interleaved. The ITI was 15–45 s on day 1 and 20–100 s subsequently. This version of the task was used to train the animals that were subjected to intra-NAc recordings without injections (Figs. 1–3; Supplementary Figs. 1–5).
  iii. *The S+/S− task with parameters modified (Fig.* *4a**)*: The third version of the task was used in the experiments that combined electrophysiological recordings with intracranial microinjections (Figs. 4, 6–8; Supplementary Fig. 6–11). The task described in (b).ii facilitates quick learning, but because of the long ITIs, some sessions were ~1 h 20 min long. To ensure that animals were under the effects of AP5 throughout the whole session, sessions were shortened to ~ 35 min by reducing the number of trials (70 trials in total, 35 of each kind) and by keeping the ITI between 15–45 s every day of training. To promote learning under these circumstances, two small changes were introduced in this version: (a) the first trial was always an S+ presented 5 s after session onset and lasting for a maximum of 60 s (increasing the likelihood of obtaining a reward early during the session boosts engagement); (b) when animals entered the reward compartment before S+ offset, the cue remained on for two additional seconds rather than turning off immediately (so that the cue and the reward were experienced simultaneously).
- *Extinction test*: Some animals received an extinction test at the end of training. This test was similar to a normal session but only cues, never rewards, were presented. This prevented within-session learning from taking place, allowing us to test the degree to which animals had learned to respond to the reward-predictive cue during the training phase.

### Surgeries

Rats were anesthetized with isoflurane, set in the stereotaxic apparatus and their scalp was retracted. Bone screws were inserted in the surface of the skull for enhanced support of the implant and holes were drilled above the target structure. Fourteen animals—the ones that were only subjected to microinjections, not recordings (Fig. 5)—were implanted with cannulae. All of the other animals were implanted with cannulated microelectrode arrays. Implants were fixed to the skull and screws using a combination of permanent glass ionomer cement and acrylic dental cement. Rats were treated with intraperitoneal injections of an analgesic solution (Ketofen), subcutaneous injections of antibiotic (Baytril), and topical antibiotic powder (Neopredef).

- *Cannulae surgery*: Bilateral guide cannulae (27-gauge; Plastics One, Roanoke, VA) targeting the NAc core were chronically implanted for microinjection. The coordinates for the tips of the cannulae were, from bregma: AP, +1.2 mm; ML, ±2 mm; DV, −5.8 mm (the tips of the injectors protracted 2 mm from the tips of the cannulae, so the actual DV coordinate of the injection was −7.8 mm). In order to prevent debris from clogging the cannulae, steel obdurators and dust caps (Plastics One) were used.
- *Cannulated microelectrode array surgery*: Cannulated microelectrode arrays were assembled prior to the surgeries^63^. Each one of these arrays consists of eight tungsten microwires (A-M Systems, Sequim, WA) surrounding a 27-gauge microinjecion guide cannula. Before assembly, each microwire’s impedance was tested to ensure it was in the 90–110 MΩ range. Both the microwires and the cannula were assembled into a custom-designed plastic drive with two parts connected by a screw that allowed dorsoventral displacement of the bundle of microelectrodes and cannula with no rotation of the probes (each full turn of the screw displaced the array ~300 μm). Finally, the microelectrodes and a silver ground wire were soldered onto connectors (Omnetics, Minneapolis, MN) that were cemented behind the cannulated microarrays. The silver ground wire was wrapped around one of the most posterior screws and then inserted inside the brain about 0.7 cm deep. The coordinates for the tips of the arrays were, from bregma: AP, +1.44 mm; ML, ±1.5–1.6 mm; DV, −6.5 to −7 mm (the tips of the injectors extended 0.5 mm beyond the tips of the cannulated array once inserted).

### Drugs

The competitive NMDA receptor antagonist AP5 (Tocris, Ellisville, MO) was dissolved either in sterile 0.9% saline (pharmacology-only experiment) or in phosphate-buffered saline (PBS) 0.1 M (all other experiments), divided in aliquots, and stored in a −20 °C freezer for up to a month before being used. The chosen concentration (2 µg/1 µl) has consistently been found to disrupt appetitive conditioning when bilaterally infused into the NAc^17–19^. The solution in which the drug was mixed (either sterile 0.9% saline or PBS) was used as a control solution. Aliquots of both solutions were retrieved from the freezer ~30 min before behavioral testing.

### Microinjections

- *Yoked control experiment (Fig.* *5**)*. Prior to each session during the training and test phases, all animals received bilateral intracranial microinjections. During the training phase, animals in the experimental group received daily injections of AP5 (1 µg/0.5 µl/side), whereas animals in the control or vehicle group received daily injections of 0.9% saline (0.5 µl/side). On test day, all animals were given saline injections. Drugs were delivered using microinjectors (33 ga, Plastics One) that extended 2 mm below the base of the guide cannulae, targeting the center of the NAc core. Polyethylene tubing filled with mineral oil connected the microinjectors to two 1 µl syringes (Hamilton, Reno, NV) mounted in a microinjection pump (KD Scientific, Holliston, MA). After both microinjectors were inserted into the guide cannulae, the pump was turned on, fluid was infused for 2 min (0.25 µl/side/min), and microinjectors were left in place for 1.5 min after the end of the injection to allow diffusion of the solution. Animals were then placed immediately in the operant chamber and the session started.
- *Electrophysiology experiments (all other experiments)*. Prior to electrophysiological recordings, animals received either no injection, bilateral AP5 (1.1 µg/0.55 µl/side), bilateral PBS (0.55 µl/side), or unilateral AP5 with PBS delivered into the contralateral hemisphere (doses and volumes were the same in the unilateral and bilateral injection experiments). The rate of infusion in animals with cannulated arrays was reduced to facilitate simultaneous injections and recordings without loss of signal^63^. In these animals, the flow rate was 0.046 µl/min (0.55 µl of either drug or vehicle were infused over 12 min into each hemisphere) and they took place inside the operant chamber. The setup for these infusions consisted of two 33 ga microinjectors that were affixed to two polyethylene tubes filled with mineral oil. The tubing was connected to a two-channel fluid swivel in the center of the roof of the chamber that allowed the animal to move freely without the tubes getting tangled. The top part of the swivel was connected to two other pieces of polyethylene tubing that ended at the tips of two 5 µl Hamilton syringes placed in a microinjection pump that stood atop the chamber. On days on which animals received injections while electrophysiological signals were being recorded, microinjectors were pre-loaded with the appropriate injectable solution before the session. The point where the water-based solution met the mineral oil was marked to enable post-hoc verification of the injection. Access to the reward receptacle was blocked with a metallic sheet before the animal was put in the chamber. This was done to prevent extinction of receptacle entry responses, which could delay learning, particularly early in training. The animal was gently handled and restrained, injectors were inserted into its cannulae, the recording cable was plugged into the connectors at the back of the rat’s headpiece, and the polyethylene tubing was taped onto the recording cable in a way that applied downward pressure on the injectors and locked them in place inside the cannulae. After the microinjection pump stopped, the mark in the fluid lines was checked to ensure that the injection had been properly delivered, the reward receptacle was reopened, the door of the operant chamber was closed, and the session started.

### Behavioral indices

Several variables were used to quantify the strength of cued approach behavior:

- *S+ entry probability*: Number of S+ trials in which the animal made a receptacle entry while the cue was on divided by the total number of S+ trials during the session.
- *S− entry probability*: Number of S− trials in which the animal made a receptacle entry while the cue was on divided by the total number of S− trials during the session.
- *ITI entry probability*: Number of S+ trials in which the animal made a receptacle entry during the 10 s window that preceded the S+ divided by the total number of S+ trials during the session.
- *S+ latency*: Latency to enter the reward compartment upon S+ onset.
- *S− latency*: Latency to enter the reward compartment upon S− presentation (for both S+ and S− latency measures, a 10 s maximum latency was assigned if no entry was made during the 10 s duration of the cue).
- *ITI pseudolatency*: Latency from the point 10 s prior to cue onset to the first receptacle entry prior to cue onset. If no entry was made during this 10 s ITI window for a particular trial, a value of 10 s was assigned to the ITI pseudolatency for that trial (Supplementary Fig. 1). In 9–15% of trials (depending on the experiment), the rat’s head was already inside the receptacle at the onset of this ITI window. Those trials were assigned the average ITI pseudolatency of the set of ±5 trials surrounding that trial.
- *Performance index*: Latency to respond to the cue compared to the latency that would be expected from the animal’s overall response frequency (Supplementary Fig. 1). It is calculated by subtracting the cued latency from the ITI pseudolatency on each trial (S+ trials and S− trials were treated separately to calculate S+ and S− performance index, respectively). It ranges from −10 to 10, with positive values indicating that the latency to respond to the cue was shorter than that predicted by the rate of receptacle entry during the preceding ITI. Negative numbers indicate the opposite. Values around 0 indicate a lack of influence of the cue on approach behavior.

### CP analysis

It has long been recognized that group averages of performance during training fail to capture critical dynamics of individual learning curves^64–66^. In many basic learning paradigms, group averages suggest that conditioned responses emerge gradually and follow a negatively accelerated curve. However, some authors suggest that the transition from a phase of no progress to a phase of mastery is abrupt rather than gradual^20^. An alternative for quantifying the learning curve involves identifying the trial after which there is a consistent expression of cued behavior. This trial is called the CP. In order to identify the CP, we used a variation of the method used in Gallistel et al.^20^.

Gallistel et al.^20^ suggest that the first appearance of conditioned responding can be identified by inspecting the cumulative record of each animal’s responses as a function of the trials experienced up to that point. These charts are a powerful tool in the identification of behavioral trends^67^ because random changes in behavior from one trial to the next are minimized while steady changes in performance are emphasized by changes in the slope of the line. These records sometimes undergo small changes in slope, and Gallistel et al.^20^ propose a method for quantifying the significance of these putative CPs. The method is a recursive algorithm that is successively run over each data point in the cumulative record of an animal. It has four steps (Supplementary Fig. 2). First, it measures the degree of deviation of a particular data point from the cumulative record by drawing a straight line from that data point to either the very first trial or the previous CP (whichever is closest to the trial to which the algorithm is currently being applied). Second, it finds the point that maximally deviates vertically from the straight line, and this trial becomes the putative or test CP. The algorithm then calculates the strength of the evidence that the distribution of trials after the test CP is different from the distribution of trials before the test CP (i.e., the log of the odds against the hypothesis that the test CP is not a true CP). In the third step, if the strength of the evidence is larger than a user-set logit value—we used the most sensitive value proposed by Gallistel et al. ^20^, logit = 1.3, which corresponds to *p* < 0.05—the algorithm truncates the data and treats that true CP as the new origin (it becomes a candidate CP). Finally, the algorithm repeats the process using the new origin. Using this algorithm, each rat’s individual record was broken down in a series of candidate CPs that identified changes in slope that are maintained across trials.

Gallistel et al. ^20^ used the first upward change in the slope of cumulative behavioral responses as a function of trials to identify the trial in which the conditioned behavior debuts. We used cumulative records of the performance index (see Behavioral indices) for the CP calculation. Unlike the cumulative conditioned response used by Gallistel et al. ^20^, the performance index can exhibit both positive and negative values. If one of the candidate CPs identified by the algorithm was followed by a negative slope, that would suggest that, for a series of trials, the animal was consistently responding more vigorously in the absence of the cue than in its presence. Even though it is not uncommon to observe fluctuations in conditioned behavior after it is acquired, it is unlikely that an animal that has truly learned the meaning of the cue would suddenly cease responding to the cue for a series of trials while maintaining a high overall rate of responding during the ITI. For that reason, we added an additional criterion to the algorithm: identification of a definitive CP (the first trial after which there is evidence of robust conditioned responding) required that the slopes of all the subsequent inter-candidate-change point segments were positive (Supplementary Fig. 2). This is the CP used in our analyses, and is referred to as the CP throughout this work.

### Acquisition of neural data

On recording days, a headstage containing unity-gain operational amplifiers was plugged into connectors that were cemented to the animal’s skull. A recording cable extended from the headstage to a multichannel commutator above the chamber, allowing the animal free movement within the operant chamber. Signals were amplified (2000–20,000×) and band-pass filtered at 250 Hz and 8.8 kHz before being sent to 40 kHz multi-unit acquisition processors. Once animals were tethered to the recording cable, each of the 16 channels was monitored using SortClient (Plexon Inc., Dallas TX) and the threshold and gain were adjusted to optimize the signal.

Electrophysiological recordings taken during behavioral sessions were subsequently processed using Offline Sorter (Plexon). Putative neurons were manually defined by identifying clusters of spikes in a 3D feature space. In that space, different combinations of each spike’s features—that is, waveform projection onto its principal components, difference between the maximum and minimum amplitude of the waveform, and the waveform height at a particular point in time— were represented on the *X*, *Y*, and *Z* axes. In order to be identified as such, an individual neuron had to show a clear and consistent waveform (>75 µV) and <0.1% of the interspike intervals could be <3000 µs. Cross-correlograms were also used to make sure that there was no overlap among different units identified within one single channel.

Given the nature of our inquiry, monitoring activity of the same neuron across days would be ideal. However, the exact location of the tips of the electrode arrays may slightly shift across days, and it would be virtually impossible to track specific neurons from one session to the next and distinguish them from new neurons that may appear on the record for the first time. The resulting data set would be a statistically unmanageable combination of repeated and non-repeated measures. Consequently, the commonplace practice of ventrally advancing electrode arrays in between sessions was adopted, thus ensuring that a new population of neurons would be recorded the next day. Arrays were lowered by turning the microdrive screw about half a turn (~150 µm). One drawback of this approach is that potential changes in neural activity observed during training could be accounted for by the changing location of the probes along the dorsoventral axis rather than by the increasing degree of exposure to the task throughout training. In order to address that possible confound, electrode arrays were kept in place during training in a group of eight rats as a control (Supplementary Fig. 6). Occasionally, no neurons were recorded on a particular day (either because the electrode arrays failed to capture any signals above the specified voltage threshold or, more rarely, because of technical difficulties with the recording system during session). If that happened, rats were run anyway to keep the training schedule consistent across subjects.

Neurons were classified as significantly cue-excited if their firing rate exceeded the upper limit of the 99.9% confidence interval of a Poisson distribution comprised of the 5 s pre-cue baseline for at least three consecutive 50 ms bins (up to 500 ms after cue onset). Neurons were classified as significantly cue-inhibited if their firing rate fell below the lower limit of the same 99.9% confidence interval for at least two consecutive 50 ms bins within the post-cue 500 ms window. For units to be classified as significantly excited (or inhibited) upon S+, S−, or ITI receptacle entry, their firing rate in the 1500 ms window after receptacle entry had to exceed the upper limit (or fall below the lower limit, in the case of inhibitions) of the 99.9% confidence interval of a Poisson distribution calculated based on the 5 s pre-cue baseline for at least six consecutive 50 ms bins. Offline Sorter files were saved as NeuroExplorer (Plexon) files and later imported as spike timestamps into R.

### Statistical analysis

Analyses of imported NeuroExplorer and Med-PC files were performed using custom routines in the R software environment. A summary of all the statistical tests shown in the main and Supplementary Figures can be seen in Supplementary Table 1. Whenever multiple comparisons were conducted on the same set of observations, *p* values were adjusted using the Holm–Sidak correction. Throughout the study, *p* values smaller than 0.05 resulted in the rejection of the null hypothesis. Labels indicate whether the data shown in the figure come from all neurons or only those that were classified as significantly excited or inhibited by the event of interest.

Unless otherwise indicated, firing rate data was converted to *Z*-scores using the 2 s before cue onset as the baseline window. Comparisons of cue-evoked firing rate across conditions within the same neuronal population were conducted using Wilcoxon’s signed-rank tests. Wilcoxon’s rank-sum tests were used for comparisons across different neuronal populations. Proportions of significantly cue-excited or cue-inhibited (and entry-excited or entry-inhibited) neurons were compared using Fisher’s exact test for count data. The relationship between the magnitude of S+-evoked phasic signals and cued approach behavior was also evaluated. To do so, the post-S+ firing rates of all the units recorded on a given session were averaged and plotted as a function of the average performance of that animal on that session according to three different behavioral indices: S+ latency, S+ response ratio, and S+ performance index. Simple linear regression models were used to quantify the relationship between these variables. Observations whose Cook’s distance deviated more than three times from the average Cook’s distance were classified as outliers and excluded from the analysis (none of the results were substantially different when outliers were included, both versions of the analysis are always shown in Supplementary Table 1). A similar approach was used to quantify the relationship between firing rate after cue onset and after receptacle entry.

When animals learned the task successfully, behavioral and neural data from each rat was aligned to the trial in which the CP took place and binned with respect to that trial. One disadvantage of aligning neural data to the trial in which the behavioral CP took place before averaging it is that two consecutive trial bins may contain data from the same neuron—which would not be the case if the data were simply averaged by session in order, because different populations of neurons were recorded every day. This makes it difficult to compare firing rate between two consecutive bins, because some of the data points in those two bins would have been recorded from independent samples (i.e., two different neurons on two different days), while others may have been recorded from the same sample (i.e., same neuron on the same day, two different sets of trials). To prevent violating assumptions of dependence/independence of observations required by the statistical tests that were employed, only non-consecutive bins were compared (e.g., it would be impossible for a neuron to contribute data to two non-consecutive 40-trial bins when each neuron was only recorded for 40 trials).

To test whether injections affected baseline firing, pre- and post-injection baseline firing rates were plotted against each other and the 99% confidence interval was constructed around the slope of the resulting regression line. We considered that baseline firing rate was unaffected by the injections if the slope of the regression line did not significantly differ from the unity line (its confidence interval included the value 1). Firing rate during the 5 s window before cue onset was used to define baseline activity in this analysis.

Paired *t* tests were used to compare performance before vs. after the behavioral CP. Mixed two-factor analysis of variances (ANOVAs) with injection (AP5 vs. vehicle) as a between-subject factor and bin (each 30 min bin of the session) as a within-subject factor were used to test the effects of AP5 on performance index throughout the test session in the moderate training. In the extended training group, injection (AP5 vs. vehicle) was treated as a within-subject factor. Per bin post-hoc comparisons were conducted with two-sample one-tailed *t* tests. Unpaired and paired *t* tests were used for between- or within-subject comparisons, respectively. Welch’s *t* tests were used when the assumption of unequal variances was violated. A two-factor ANOVA was also used to compare performance during the extinction test session (Fig. 8). The Greenhouse–Geisser correction was applied whenever the assumption of sphericity was violated.

### Histology

At the end of behavioral training and testing, animals were deeply anesthetized with Euthasol (39 mg/kg pentobarbital) and intracardially perfused with saline and a 4% paraformaldehyde solution. Their brains were removed after decapitation and then stored in 4% paraformaldehyde solution until further processing. After rinsing them with PBS 0.1 M, all brains were sliced into 50 µm sections using a vibratome. Sections were later mounted on slides and stained with cresyl violet to facilitate verification of injection and recording locations^68^ (Supplementary Fig. 12).

### Reporting summary

Further information on research design is available in the Nature Research Reporting Summary linked to this article.

## Supplementary information

Supplementary Information

Reporting Summary

## Data Availability

The datasets that support the findings of the current study are available from the corresponding author upon request.

## Code availability

The custom code used in this study is publicly available in the following repository: https://github.com/mvegavillar/Accumbens-Rew-learning
