# Supplementary material for: NMDA receptor-dependent plasticity in the nucleus accumbens connects reward-predictive cues to approach responses
Source: Nat Commun. 2019 Sep 27;10:4429. doi: 10.1038/s41467-019-12387-z (PMC6764993; doi:10.1038/s41467-019-12387-z)
Supplement: Supplementary file 1 — Supplementary Information [file 41467_2019_12387_MOESM1_ESM.pdf]

NMDA receptor-dependent plasticity in the nucleus accumbens connects reward-predictive cues to approach responses

Vega-Villar et al.

SUPPLEMENTARY FIGURES

Supplementary Figure 1

a

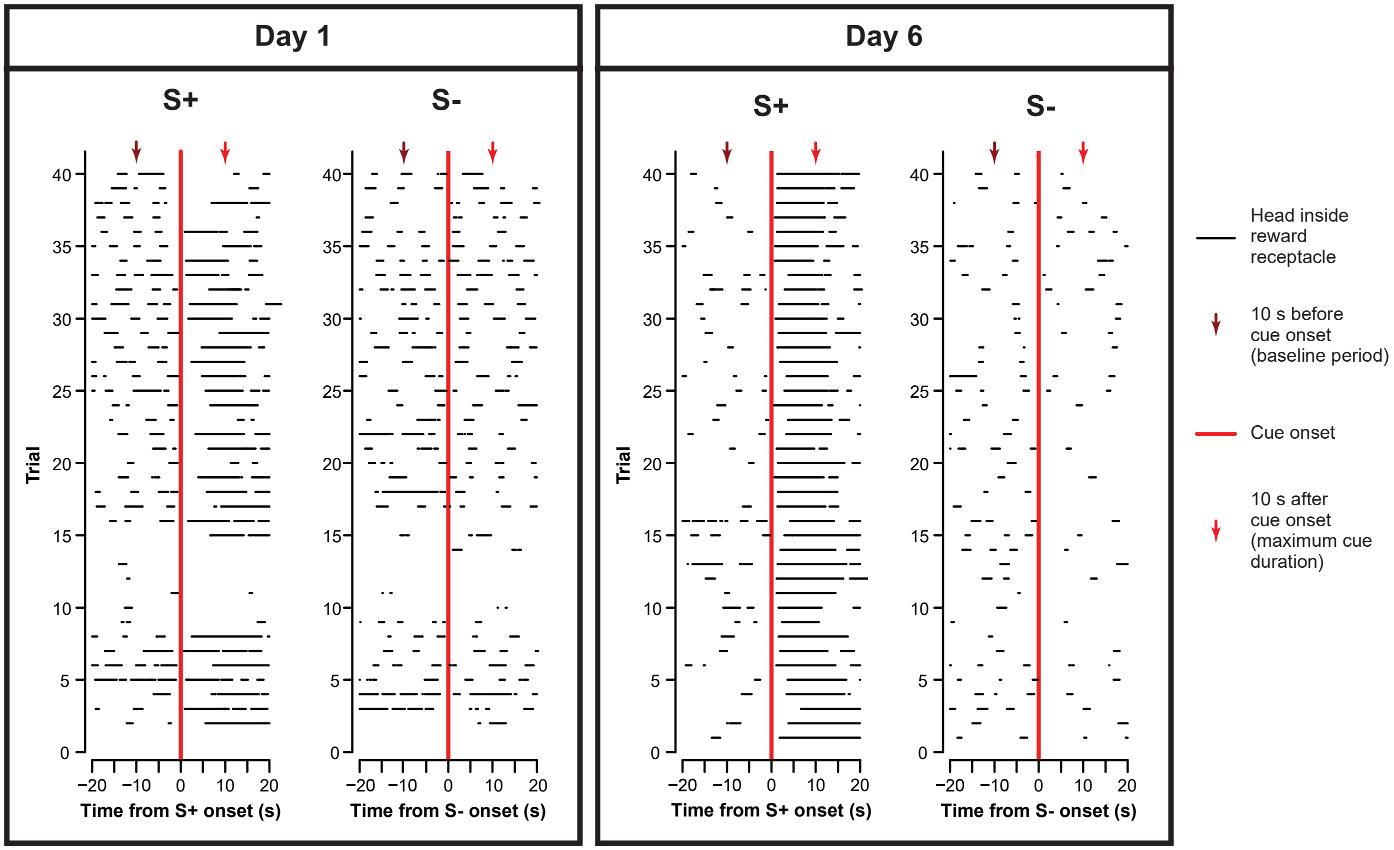

b

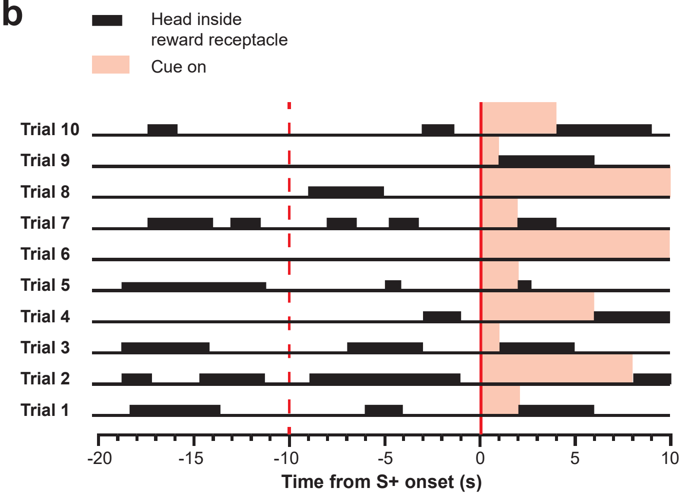

Trial number	ITI pseudolatency	Cued latency	Performance index *(ITI pseudo. - Cued lat.)*
10	7 s	4 s	7 s - 4 s = **3 s**
9	10 s	1 s	10 s - 1 s = **9 s**
8	1 s	10 s	1 s - 10 s = **-9 s**
7	2 s	2 s	2 s - 2 s = **0 s**
6	10 s	10 s	10 s - 10 s = **0 s**
5	5 s	2 s	5 s - 2 s = **1 s**
4	7 s	6 s	7 s - 6 s = **1 s**
3	3 s	1 s	3 s - 1 s = **2 s**
2	1 s	8 s	1 s - 8 s = **-7 s**
1	4 s	2 s	4 s - 2 s = **2 s**

Min = -10 s; Max = 10 s

Supplementary Figure 1. **Performance index calculation.**

(a) Representative behavioral raster plots of one animal on the first (left, Day 1) and the last (right, Day 6) day of training. Within each panel, performance is divided into S+ and S- trials. Each trial is shown in a different row, and trials are sorted earliest to latest from bottom to top. Black horizontal lines within each trial represent periods when the rat's head was inside the reward receptacle. Data is aligned to the time of cue onset (vertical red line). Arrows mark the 10 s interval before and after cue onset. The raster plots show that, early in training, an overall high frequency of entry into the reward receptacle may preclude the interpretation of entry during the S+ as specifically cue-driven behavior. Note that fluctuations in S+ responding are accompanied by fluctuations in responding during the intertrial interval. This emphasizes the need to consider the rate of indiscriminate responding (i.e., during the ITI) when quantifying cued responding.

(b) Calculating the performance index. The left panel represents hypothetical performance on ten trials aligned to the time of S+ onset (vertical red line). Pink rectangles span the duration of the S+. Black rectangles depict entries into the receptacle. A dashed red line indicates the beginning of a window beginning 10 s prior to cue onset. For each trial, two latency values were calculated: the interval from the point 10 s prior to the cue to the first receptacle entry occurring prior to the cue (*ITI pseudolatency*), and the period during which the cue was on (*cued latency*, corresponding to the interval between cue onset and receptacle entry). If no entry was made during one of these periods, a value of 10 was assigned. To calculate the performance index of the animal on a given trial, its cued latency on that trial was subtracted from its ITI pseudolatency on the same trial. The performance index ranges from -10 to 10, with negative values indicating that the animal entered into the receptacle faster in the absence of the cue than in its presence, and positive values indicating the opposite. Values around zero suggest that the cue has no influence on receptacle entry behavior. The table on the right shows ITI pseudolatency, cued latency and performance index corresponding to the trials shown in the left panel.

Supplementary Figure 2

a

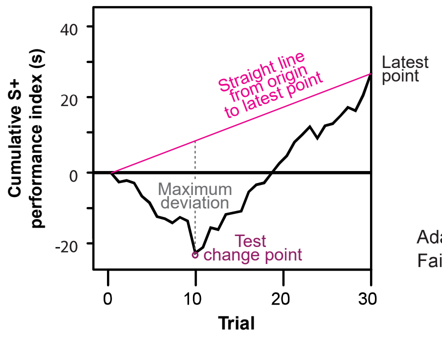

Adapted from Gallistel, Fairhurst and Balsam (2004)[1]

b

Candidate change point trial	Cumulative performance index (s)	Segment length (# trials)	Slope (Avg. S+ perf. index, s)	Positive slope
0	0	16	0.689	TRUE
16	11.023	13	-1.708	FALSE
29	-11.185	7	0.918	TRUE
36	-4.759	7	-4.575	FALSE
43	-36.787	14	-0.883	FALSE
57	-49.146	9	3.359	TRUE **Change point**
66	-18.906	29	0.756	TRUE
95	3.005	7	1.974	TRUE
102	16.824	13	4.045	TRUE
115	69.415	18	6.371	TRUE
133	184.091	14	4.135	TRUE
147	241.985	8	0.927	TRUE
155	249.396	23	4.200	TRUE
178	346.002	6	8.019	TRUE
184	394.116	17	4.921	TRUE
201	477.783	8	1.871	TRUE
209	492.752	24	4.071	TRUE
233	590.467	8	7.005	TRUE
241	646.510	14	2.312	TRUE
255	678.884	14	3.053	TRUE
269	721.624	11	6.864	TRUE
280	797.135	0	*	*

c

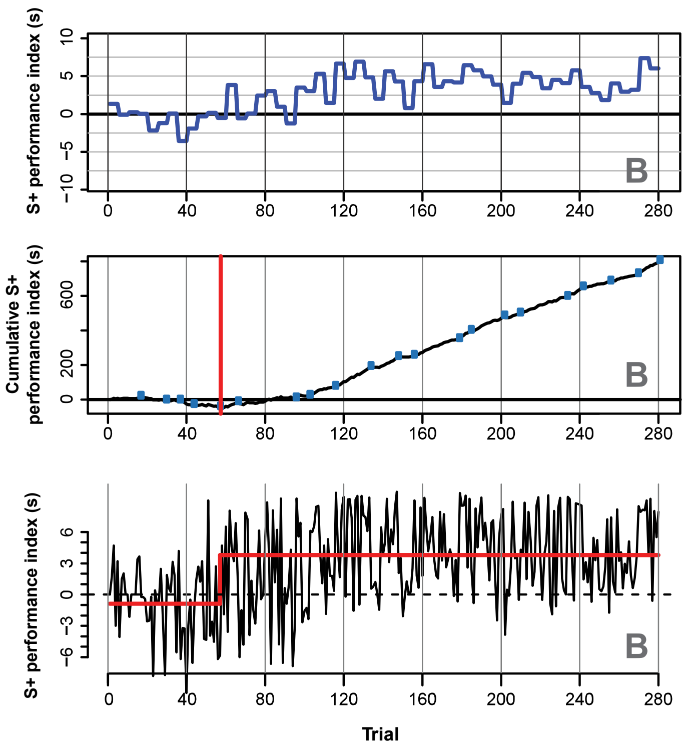

Supplementary Figure 2. **Identifying the behavioral change point.**

(a) Hypothetical application of the change point algorithm to the cumulative record as of trial 30 (adapted from Gallistel, Fairhurst and Balsam, 2004[1]). First, a straight line is drawn from trial 30 to the origin. Second, the trial that maximally deviates from that line is identified as a potential candidate change point (test change point). Third, performance values before and after the test point are compared. If the null hypothesis of no change can be rejected at a user-specified significance value – we chose $p < 0.05$ (logit = 1.3) – that test change point is considered a candidate change point. The algorithm then truncates the data at that point and treats that candidate change point as the new origin. Finally, the algorithm starts the process all over again, running successively over each trial in the cumulative record.

(b) The result of this iterative algorithm is typically a list of candidate change point trials. Gallistel et al.[1] take the first candidate change point in the cumulative record as the definitive change point – the first trial after which cued behavior can be consistently detected. However, they applied the algorithm on behavioral variables that can only adopt null or positive values, which yield cumulative records in which the change of the slope can only detect an improvement in behavior or lack of thereof (i.e., the slope can only be positive or 0). In contrast, our performance index can also capture instances in which the animal's likelihood or speed of cued responding is less than what would be expected from its baseline behavior. As a result, at the beginning of training, it is not unusual to find brief increases in the slope of the line followed by decreases. For that reason, for a candidate change point to be identified as definitive in our paradigm, the subsequent segments between candidate change points in the cumulative record had to have a positive slope, or the candidate change point was rejected. The slope of these segments could fluctuate – as is common for conditioned behavior even after it is acquired – but it could not be negative. Therefore, we determine the definitive change point as the first candidate change point for which all subsequent slopes are positive, and we report this trial as the change point (CP) in the main text. This trial corresponds to that on which consistent cued behavior first appears.

(c) Sample performance of one subject ("B") throughout training in three graphs. Gray lines indicate the transition between sessions. *Top*: average S+ performance index in five-trial bins (blue). *Middle*: cumulative S+ performance index record. Blue dots mark all of the candidate change points identified by the algorithm. The vertical red line marks the change point. *Bottom*. Trial by trial S+ performance (black) and average performance before and after the change point (red).

Supplementary Figure 3

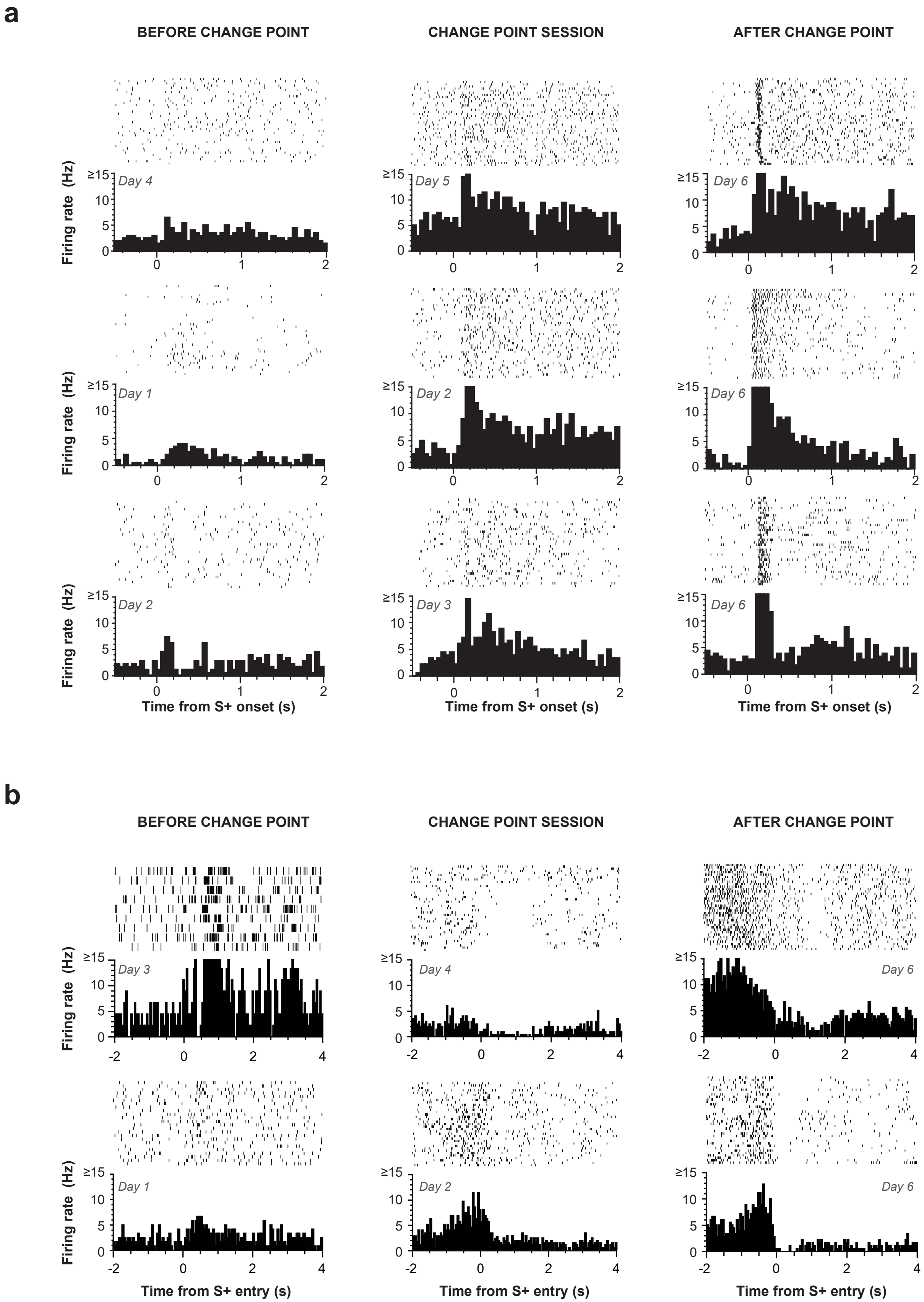

Supplementary Figure 3. **Representative individual neurons at different points of training.**

(a) Sample perievent time raster plots (*top*) and histograms (*bottom*) aligned to the time of S+ onset. Each row of graphs shows three representative neurons of the same animal, one recorded on the day before change point (*left*), another one recorded on the change point session (*middle*) and the last one recorded on the sixth day of training (*right*). Dots in the raster plots represent action potentials fired by the recorded neuron and trials are sorted from earliest to latest from top to bottom. Histograms were converted to firing rate using 50 ms bins. The y-axis of histograms is capped at 15 Hz to facilitate comparison across neurons. “Day” numbers refer to the training day.

(b) Same as “(a)” but with neuronal data aligned to the time of receptacle entry during the S+.

Supplementary Figure 4

Post-cue (100-400 ms) firing by session and breakdown of session 1

a **b**

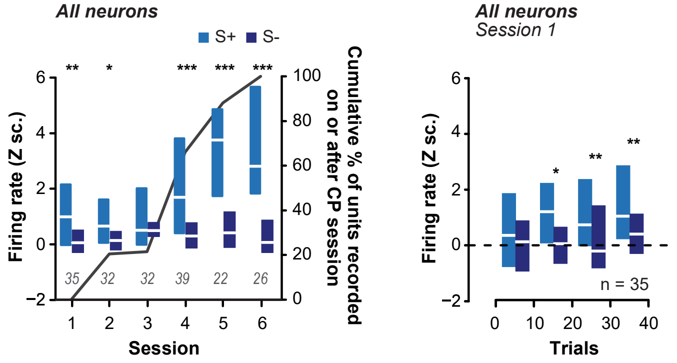

Post-cue (100-400 ms) firing by sensory modality of the cue

c

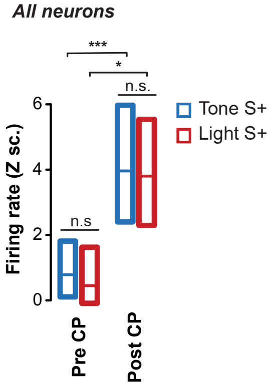

Duration of cue-evoked excitations

d

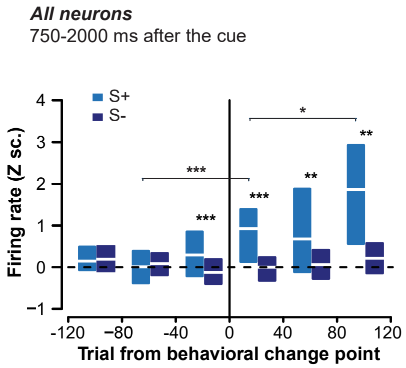

e

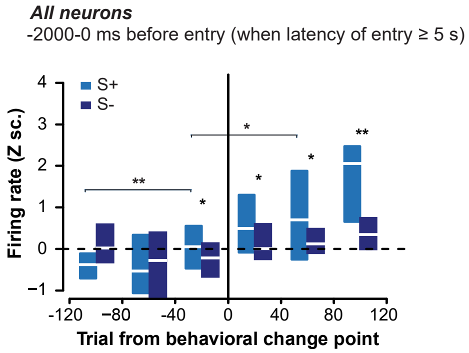

Post-cue (100-400 ms) firing by cue/response combination

f

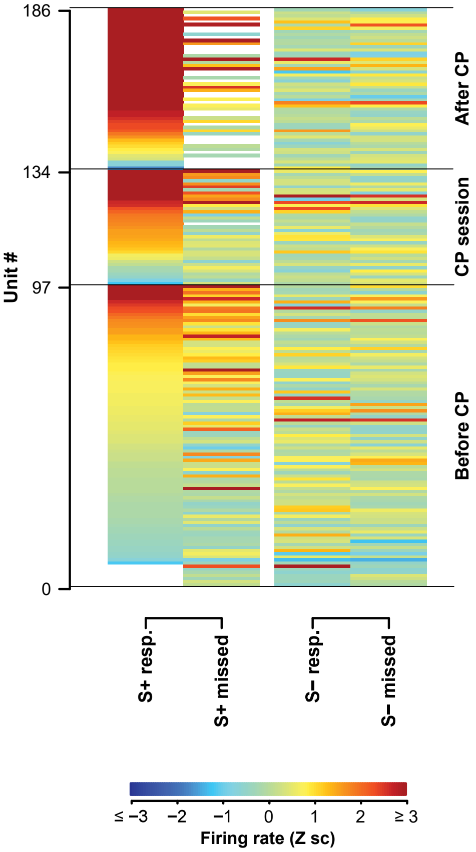

Supplementary Figure 4. **Additional graphs showing NAc core firing activity during training.**

(a) Population firing rate (median and interquartile range) in the 100-400 ms window after S+ (light blue) or S- (dark blue) onset by session. Numbers indicate sample size. The gray line indicates the cumulative percentage of units recorded from animals that exhibited a behavioral change point on or before that session. Post-cue firing was higher in S+ than S- trials in most sessions (*$p < 0.05$; **$p < 0.01$; ***$p < 0.001$, Wilcoxon).

(b) Same as "(a)" but only for the first session in 10-trial bins. Firing rate was higher after S+ than S- onset after only 10 trials (*$p < 0.05$; **$p < 0.01$; ***$p < 0.001$, Wilcoxon).

(c) Population firing rate (median and interquartile range) in the 100-400 ms window after S+ presentation when the S+ was a tone (blue) or a light (red) in the sessions before (left, "Pre CP") or after change point (right, "Post CP"). There was no main effect of the sensory modality of the cue (Tone vs. Light; $F_{1,145} = 0.006$, $p = 0.9403$).

(d) Same as Fig. 2c but for the 750-2000 ms post-cue window. Starting just before behavioral change point, firing rate after S+ onset was higher than after S- onset in this window (**$p < 0.01$; ***$p < 0.001$, Wilcoxon).

(e) To test whether the firing rate of NAc neurons was elevated prior to receptacle entry in S+ trials even when the latency to enter was long, we calculated the firing rate during the pre-entry 2 s window in trials during which it took animals 5 s or more to make a receptacle entry. Starting before behavioral change point, pre-entry NAc firing rate was higher in S+ than S- trials even when the latency to enter the reward receptacle was over 5 s (*$p < 0.05$; **$p < 0.01$, Wilcoxon).

(f) Each line depicts the average firing rate of each recorded neuron in the post-cue 100-400 ms window after S+ and S- cues that subjects responded to (*resp.*) or missed. Units are divided into three blocks depending on whether the session in which they were recorded was before the behavioral change point (Before CP), the session during which the change point took place (CP session) or after the change point (After CP). Within each block, neurons are sorted from top to bottom in descending order according to the magnitude of their activity in the 100-400 ms post-S+ window. The legend on the right shows the correspondence between colors and firing rate values.

Supplementary Figure 5

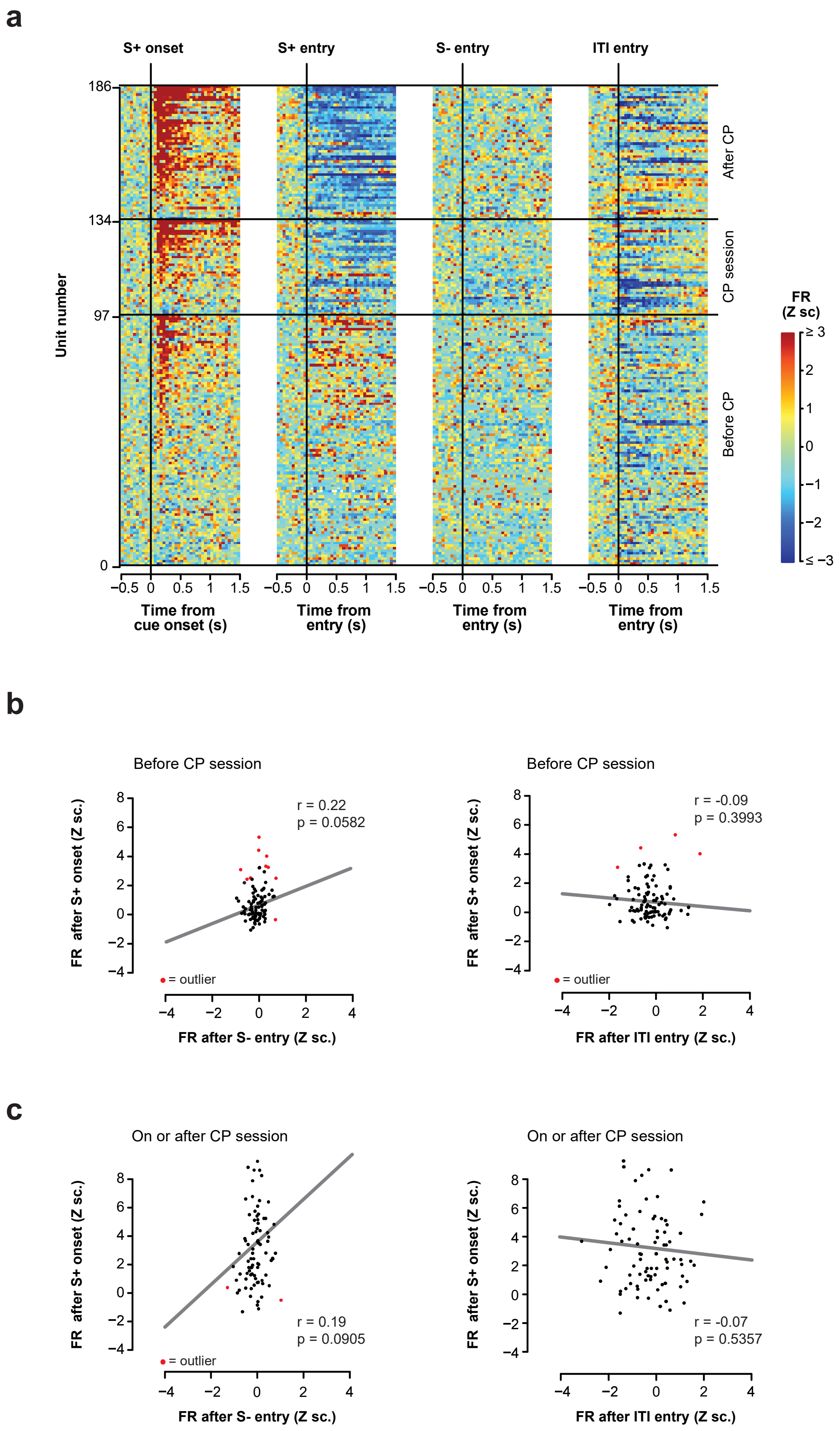

Supplementary Figure 5. Activity in the NAc around the time of receptacle entry during training.

(a) From left to right, four heat maps represent average neuronal activity around the time of S+ onset, S+ entry, S- entry and ITI entry. Across heat maps, each line represents the same neuron. Units are divided into three blocks depending on whether the session during which they were recorded took place before the behavioral change point (Before CP), on the session during which the change point took place (CP session) or after the change point (After CP). Within each block, neurons are sorted from top to bottom in descending order according to the magnitude of their activity in the 100-400 ms post-S+ window. The legend on the right shows the correspondence between colors and firing rate values (in Z scores).

(b-c) Black dots represent each neuron's firing rate in the 100-400 ms window after S+ onset plotted against the same neuron's firing rate in the 0-1500 ms window after S- (b) or ITI (c) entry before (top) and after (bottom) behavioral change point. The regression line is shown in gray and the outliers are depicted in red (a few outliers fall outside the graph axes). Outliers are excluded from the analyses that yielded the results shown in these graphs. Including those outliers did not substantially change the results (Supplementary Table 1). Firing rate after S- or ITI entry was not significantly correlated with S+-evoked firing rate before or after change point ($p > 0.05$).

Supplementary Figure 6

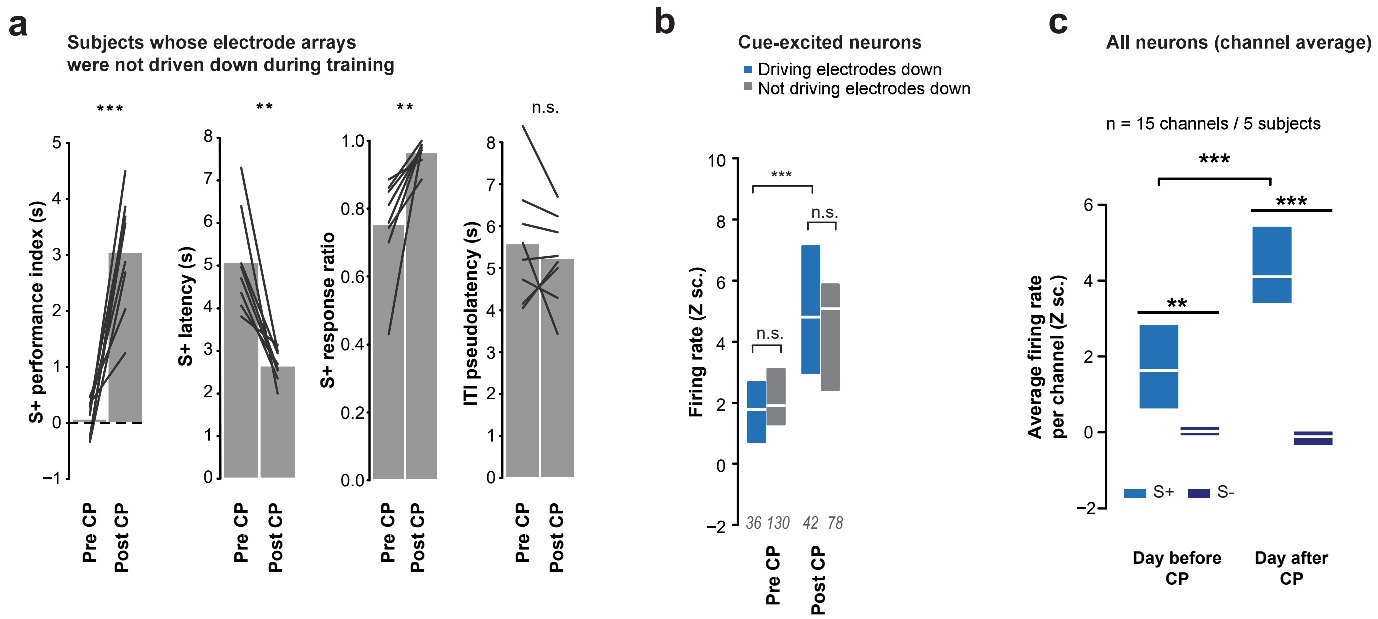

Supplementary Figure 6. NAc cue-evoked excitations emerged during training regardless of whether the electrodes were driven down in between sessions or not.

(a) For animals whose arrays were not driven down after each session, comparison of average S+ performance index (***t = -6.84, $p < 0.001$), entry probability (**t = -3.9, p = 0.0059, latency (**t = 5.24, p = 0.0018) and ITI pseudolatency (t = -1.72, p = 0.0059) before change point (Pre CP) vs. after change point (Post CP).

(b) When electrode arrays are not driven down in between sessions, the resulting data set includes recordings of some neurons that are the same across days, and others that are not. This means that data collected across days contains a mixture of repeated and non-repeated measures. This precludes the comparison between sessions using statistical inference tests, since these tests require that observations across conditions are comprised of either repeated measures samples (within-subjects comparisons) or different samples (across-subjects comparisons). Driving the electrodes down in between sessions to sample a new population of neurons each day avoids this confound, but it also introduces a potential anatomical confound when comparing neuronal activity across sessions. In order to assess whether advancement of the probes had an effect on the learning-related increase in S+-evoked firing, we compared post-S+ firing in the group of subjects whose arrays were maintained in the same location during training with those subjects whose arrays were advanced in between sessions (Fig. 2), both before and after the change point. The graph shows firing rate (median and interquartile range) in the 100-400 ms post-S+ window before change point (Pre CP) and after change point (Post CP) in cue-excited neurons of rats whose arrays were driven down (blue) or not (gray) after each session. S+ evoked activity before or after the change point is similar across groups ($p > 0.05$, Wilcoxon).

(c) Average activity per channel (in channels that captured firing rate from two or more units) on the day before (left) and the day after (right) behavioral change point during the 100-400 ms window after S+ (light blue) or S- (dark blue). Within-channel comparisons showed that activity evoked by the S+ was higher than activity evoked by the S- in both sessions. They also revealed that S+-evoked activity was higher on the day after behavioral change point compared to the day before behavioral change point (**$p < 0.01$; ***$p < 0.001$, Wilcoxon). These results suggest that the emergence of cue-evoked excitations observed in Fig. 2 are not accounted for by the dorsoventral location of the recording electrodes.

Supplementary Figure 7

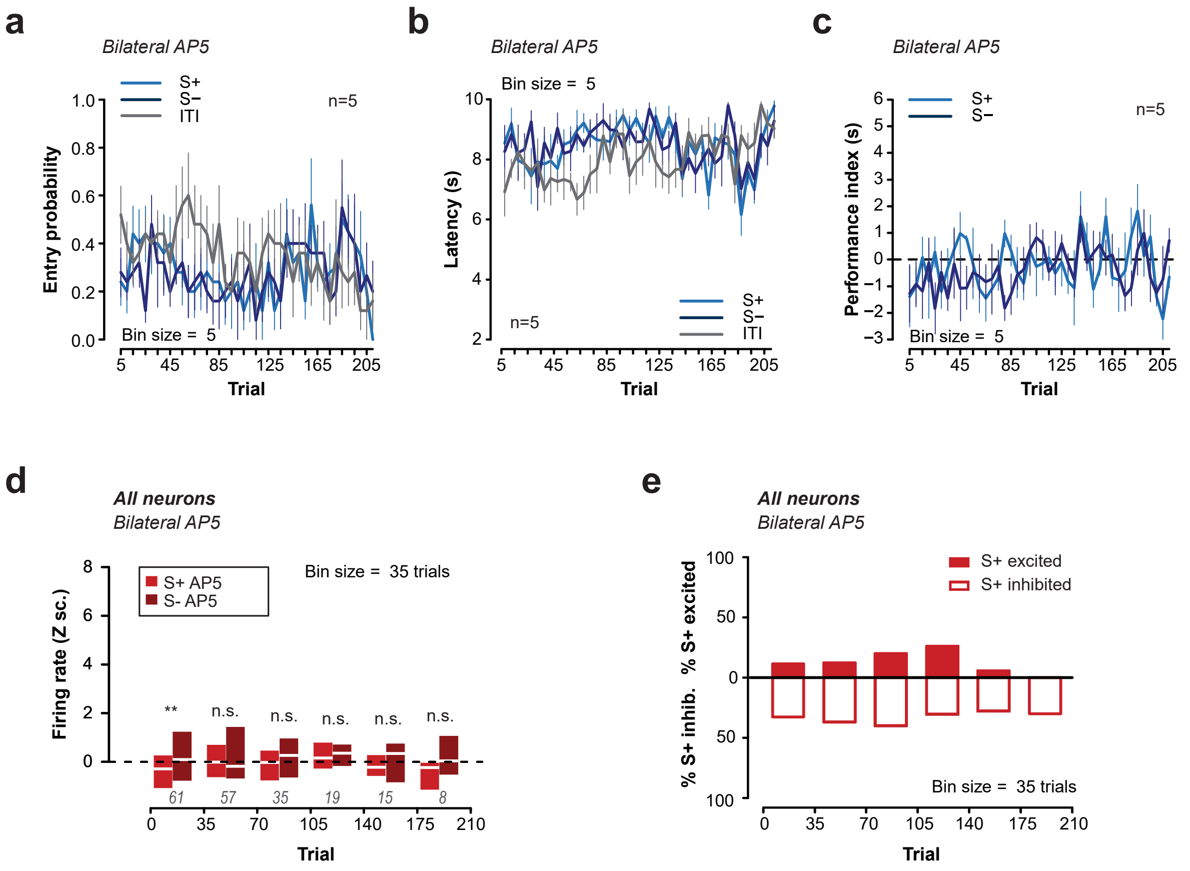

Supplementary Figure 7. Bilateral blockade of NMDARs during training disrupts the emergence of cue-evoked excitations in NAc as well as the acquisition of cued approach behavior.

(a) Mean±SEM entry probability during the S+ (light blue), S- (dark blue) or pre-S+ ITI window (gray) in animals that received daily bilateral AP5 injections prior to training.

(b-c) Same as '(a)' but for latency and ITI pseudolatency (b), and performance index (c).

(d) Firing rate (median and interquartile range) in the 100-400 ms window after presentation of S+ (light red) or S- (dark red) in 35-trial bins (each bin corresponds to a session) in animals that received daily bilateral AP5 injections. During the first session, activity elicited by the S- was higher than activity elicited by the S+ (**$p < 0.001$, Wilcoxon). Post-S+ firing was comparable to post-S- firing in subsequent sessions ($p > 0.05$, Wilcoxon). Numbers indicate sample size.

(e) Proportion of significantly excited (solid bars) or inhibited (white bars) NAc units upon presentation of the S+ in subjects that received daily bilateral AP5 injections. The proportion of neurons significantly excited or inhibited by the cue was independent of the amount of training animals had received (excitations: $p = 0.2718$; inhibitions: $p = 0.9478$, Fisher).

Supplementary Figure 8

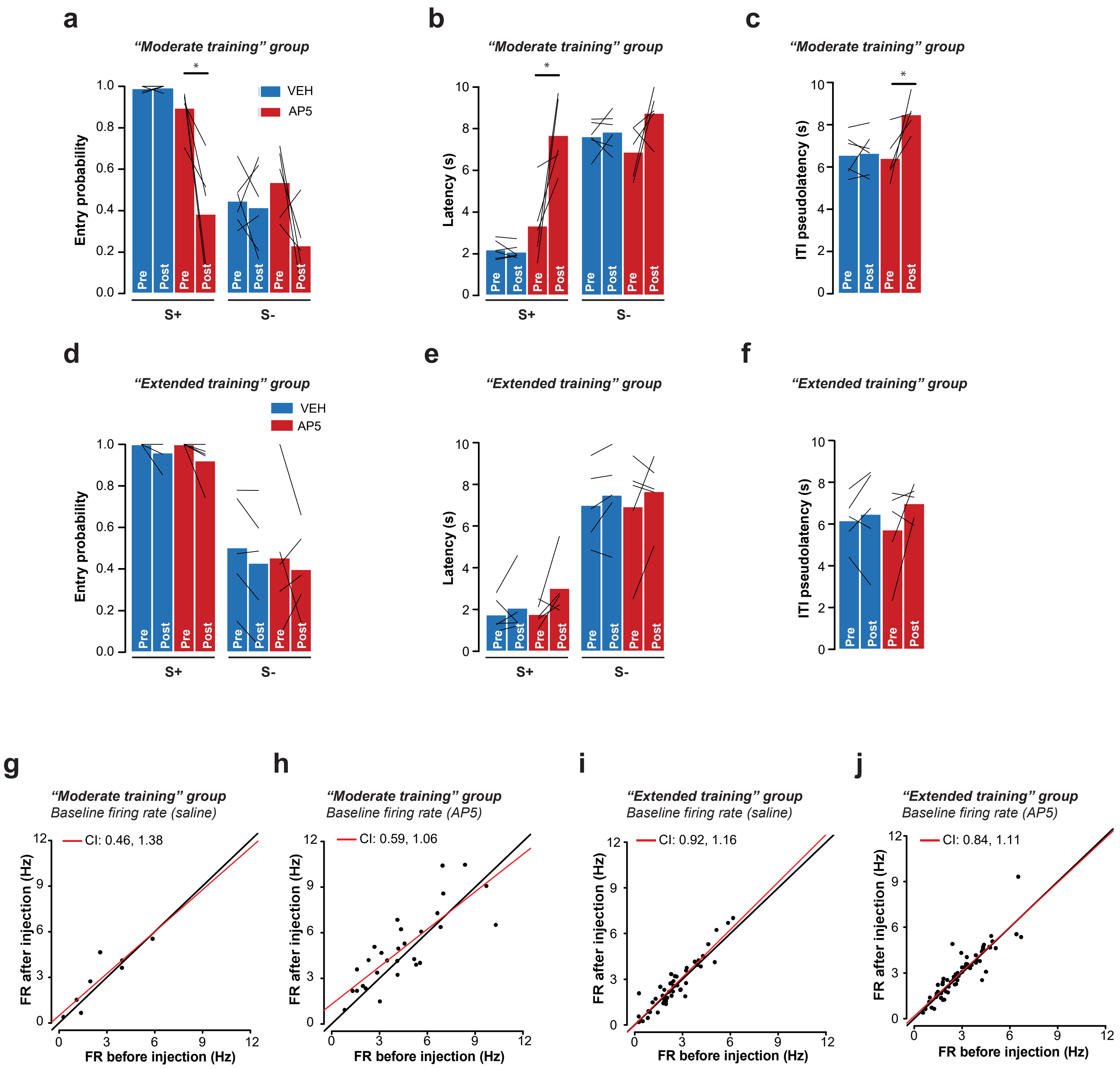

Supplementary Figure 8. Additional graphs showing how behavior and NAc core activity were affected by bilateral AP5 microinjections after moderate or extended training.

(a) Probability of entry during the S+ (left) or S- (right) before (Pre) or after (Post) infusion of vehicle (blue, n = 6) or AP5 (red, n = 5) in moderately trained animals. In S+ trials, entry probability was significantly diminished by microinjection of AP5 (*t = -3.504, p = 0.0248) but not vehicle (t = -0.445, p = 0.6624).

(b-c) Same as "(a)" but for cued latency (b) or ITI pseudolatency (c). In S+ trials, microinjections of AP5 increased the latency to make an entry during both the S+ (*t=-3.085, p = 0.0367) and the ITI period (*t = -2.916, p = 0.0434), whereas vehicle injections did not have that effect (S+: t =0.709, p = 0.7450; ITI: t = -0.229, p = 0.3881).

(d-f) Same as "(a-c)" but for animals that received extended (n =5) instead of moderate training. A two-factor ANOVA using drug and time as within-subject factors revealed no main or interactive effects in S+ or S- entry probability/latency and ITI pseudolatency (all effects: $p > 0.05$).

(g-h) Baseline firing rate before injection plotted against baseline firing rate after saline (g) or AP5 (h) injection. In both cases, the 99% confidence interval (CI) around the slope of the regression line (vehicle: 0.46-1.38; AP5: 0.59-1.06) did not significantly differ from the unity line (i.e. the confidence interval contained the value "1"), suggesting that baseline firing rate was not affected by either injection.

(i-j) Same as "g-h" but for animals that received extended training prior to the saline (CI: 0.92-1.16) or AP5 (CI: 0.84, 1.11) injection. The baseline firing rate in these animals was also unaffected by the injections.

Supplementary Figure 9

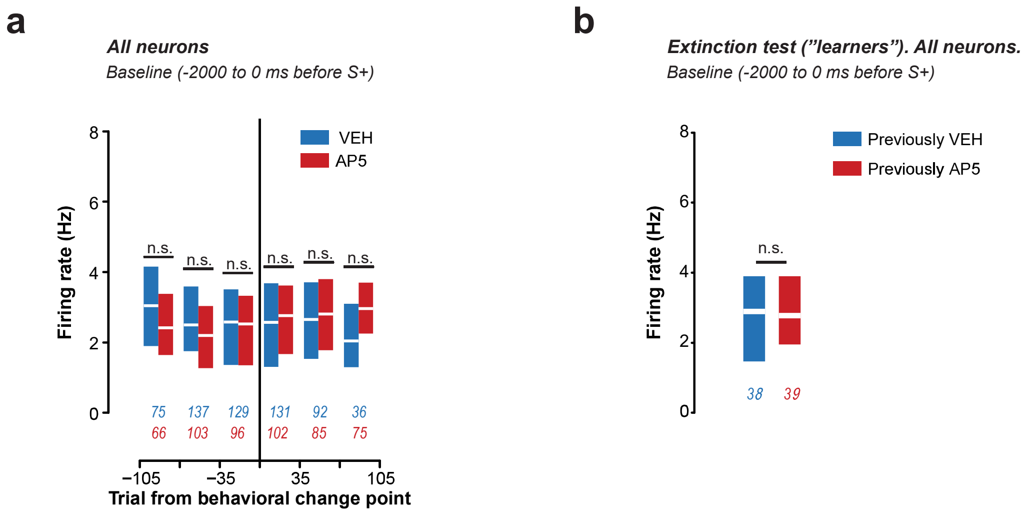

Supplementary Figure 9. Baseline firing rate in NAc core was not affected by unilateral infusions of AP5.

(a) Raw firing rate (median and interquartile range) in the 2 s window before S+ onset in the saline (blue) or AP5-treated (red) side in 35-trial bins around the trial in which the behavioral change point took place. Numbers represent the number of neurons recorded on each bin on the vehicle (blue) or the AP5-treated hemisphere (red). There was no difference in baseline firing rate across hemispheres in any of the bins ($p > 0.05$, Wilcoxon; Holm-Sidak adjusted).

(b) During the extinction test, learners' firing rate (median and interquartile range) in the 2 s window before S+ onset in the hemisphere that had been treated with saline (blue) or AP5-treated (red) during training. There was no difference in baseline firing rate across hemispheres during this session ($p > 0.05$, Wilcoxon).

Supplementary Figure 10

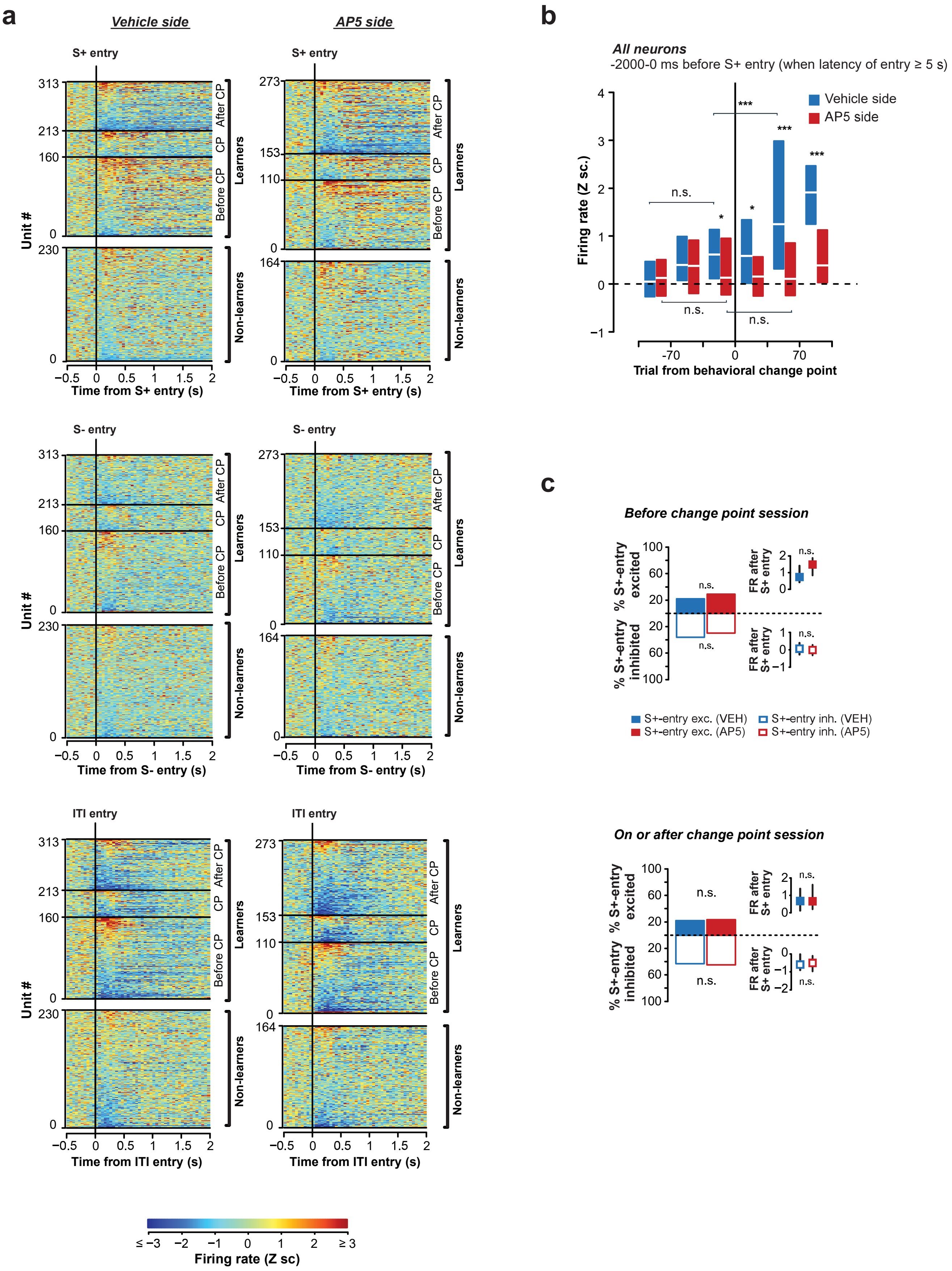

Supplementary Figure 10. NAc activity around the time of S+, S- or ITI entry in animals treated with unilateral AP5 microinjections.

(a) Heat maps representing firing rate in 50 ms bins around the time of S+ entry (top), S- entry (middle) or ITI entry (bottom) in the vehicle (left) or AP5-treated side (right) of subjects that received unilateral AP5 microinjections during training. Each line on each heat map represents a neuron. Neurons are divided into two blocks depending on whether the animal learned the task during training (learner) or not (non-learner). In the learners block, neurons are further divided into three blocks: units recorded before the change point (Before CP), during the session in which the CP took place (CP) or after the CP (After CP). Within each one of these blocks, units are sorted from top to bottom in descending order based on their average firing rate in the 0-500 ms window after the event the data is aligned to (i.e., S+ entry, S- entry or ITI entry respectively). The magnitude of the firing rate on each bin is color-coded according to the legend in the right.

(b) Firing rate during the pre-entry 2 s window in the vehicle (blue) or AP5-treated side (red) in S+ trials during which it took animals 5 s or more to make a receptacle entry. Starting before behavioral change point, pre-S+-entry firing rate was higher in the vehicle than in the AP5-treated side even when the latency to enter the reward receptacle was long (*$p < 0.05$; ***$p < 0.001$, Wilcoxon).

(c) The proportion of excited (solid) or inhibited (empty) units upon S+ entry before (left) or after (right) CP across hemispheres (vehicle: blue; AP5: red) was comparable ($p > 0.05$, Fisher). The magnitude of the post-S+-entry response of these units (insets: median and interquartile range) was also similar ($p > 0.05$, Wilcoxon).

Supplementary Figure 11

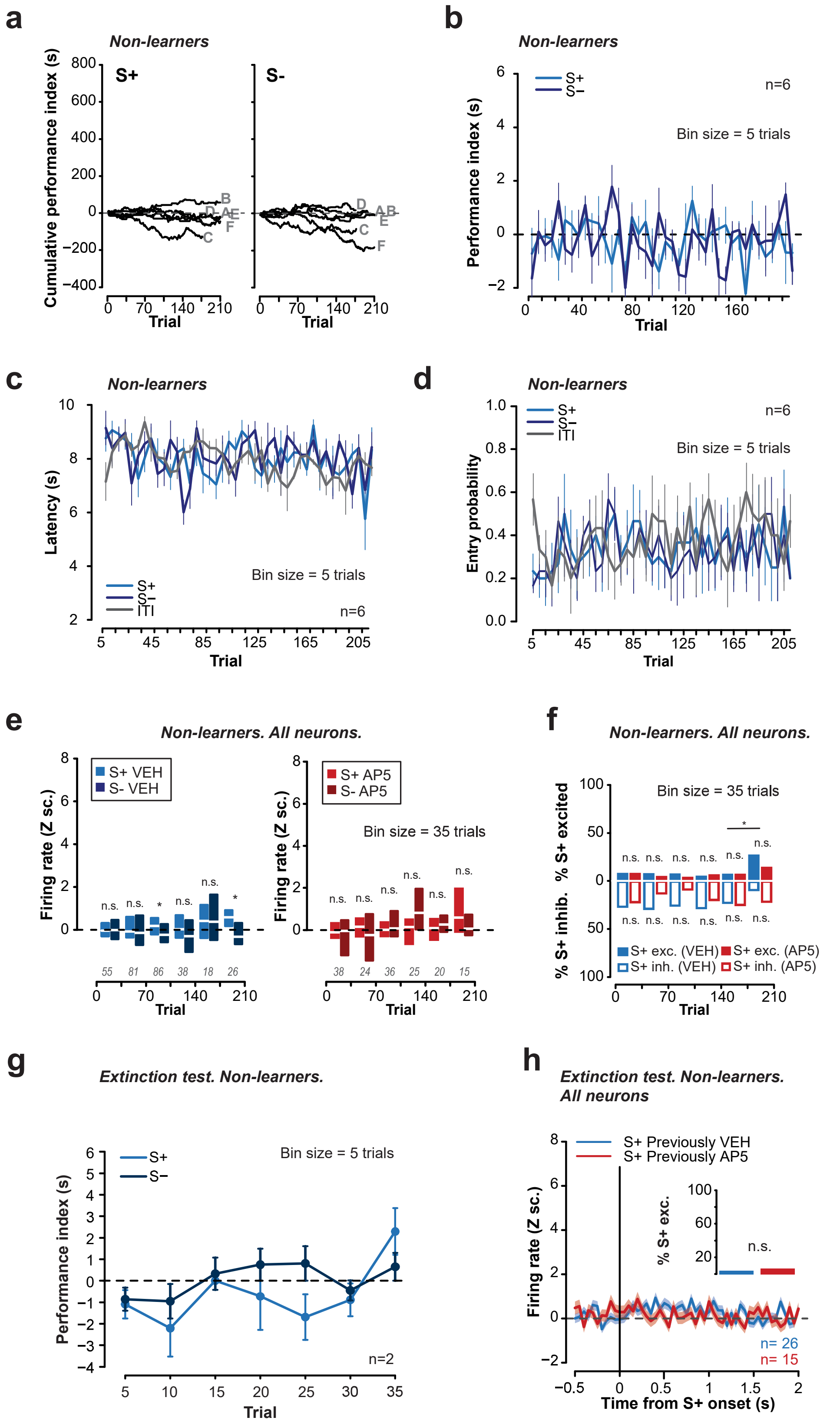

Supplementary Figure 11. Cue-evoked excitations did not emerge in the NAc core neurons of animals that failed to learn the task under daily unilateral AP5 injections ("non-learners").

(a) Individual cumulative performance index records on S+ (left) and S- (right) trials in animals that received unilateral AP5 injections and did not learn the task. Each line represents a different animal. A positive change point was not identified in their S+ performance.

(b-d) Mean±SEM performance index (b), latency (c) and entry probability (d) of non-learners in 5-trial bins throughout training. S+ trials are represented in light blue, S- trials in dark blue and, in gray, the 10 s ITI window that preceded the S+.

(e) For animals that failed to learn the task, population firing rate in NAc neurons in the vehicle (left) or AP5 (right) side in S+ trials (light blue/red) and S- trials (dark blue/red) in the 100-400 ms window after the cue. S+-evoked excitations did not emerge throughout training in any of the sides ($p > 0.05$, Wilcoxon; Holm-Sidak adjusted).

(f) The proportion of significantly S+-excited (top) or inhibited (bottom) units in the vehicle (blue) and AP5-treated (red) side of non-learners. Throughout training, the percentage of neurons whose activity was significantly modulated by the cue did not differ across hemispheres ($p > 0.05$, Fisher; Holm-Sidak adjusted). Only in the last session, there was a significant increase in the percentage of cue-excited units (*$p = 0.0465$, Fisher; Holm-Sidak adjusted).

(g) Performance index in S+ (light blue) and S- (dark blue) trials during the drug-free extinction test in the two non-learners that were given this test.

(h) Firing rate around the time of S+ onset in 50 ms bins in the vehicle (blue) and AP5 (red) sides during the drug-free extinction test in animals that failed to learn the task during training. The inset represents the percentage of units that were excited by the S+ during the drug-free extinction test in the hemispheres that, during training, received either vehicle (blue; n = 26) or AP5 (red; n =15) injections. There were no differences in the percentage of cue-excited units across hemispheres in these animals ($p = 1$, Fisher).

Supplementary Figure 12

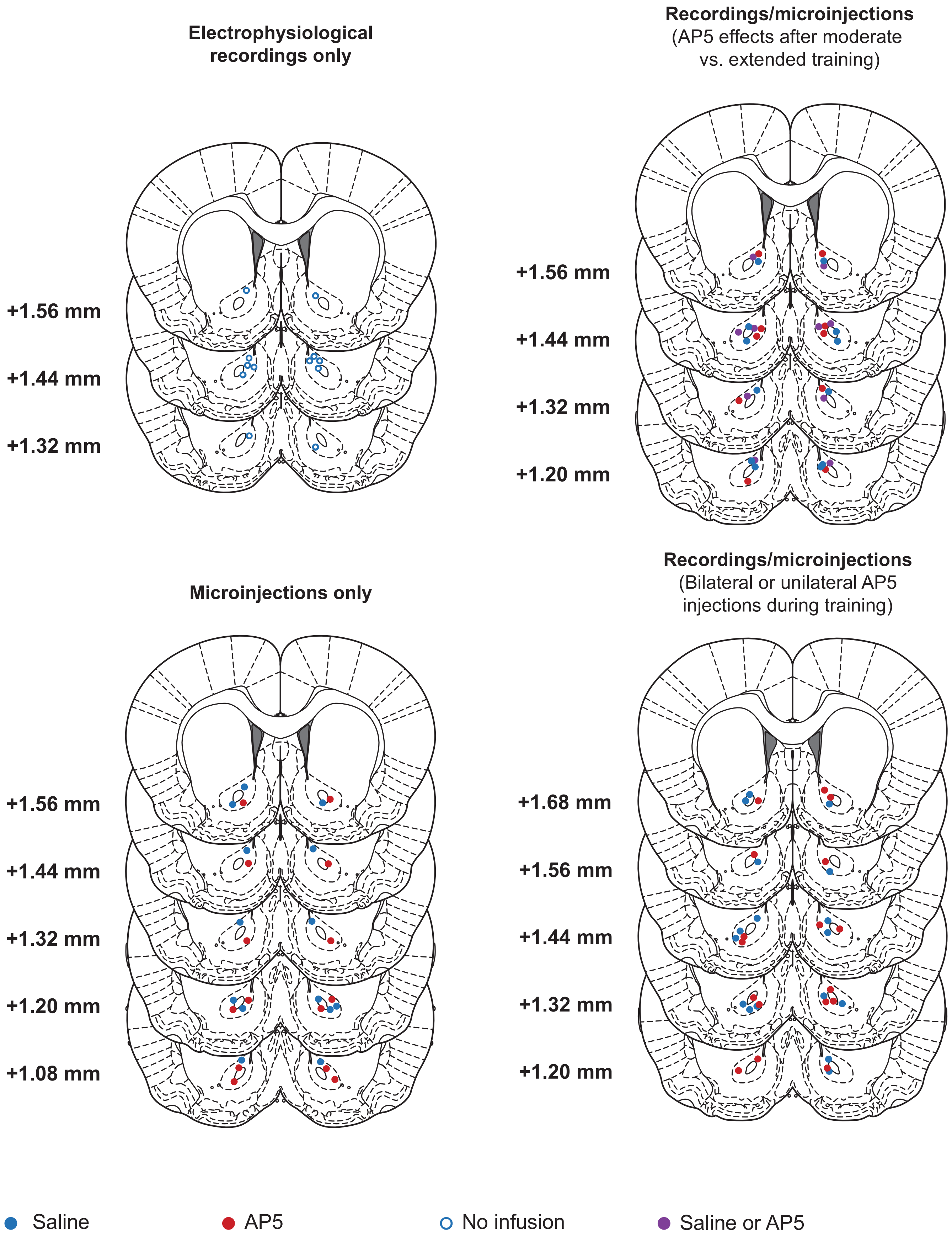

Supplementary Figure 12. Anatomical location of injection and recording sites. For each experiment, diagrams of coronal sections of rat brain at different anteroposterior coordinates[2]. In animals that received no infusions, empty blue circles mark the tips of the electrode arrays. Solid dots mark the sites where the injectors delivered saline (blue), AP5 (red) or either one depending on the session (purple).

Supplementary Table 1

A. Statistical tests in main figures.

Figure	Independent variable(s)	Dependent variable	Test	Result	Sample size
**Figure 1f**	Before vs. after change point	S+ perf. index	Paired t-test (Holm-Sidak corrected)	$t_{(5)}$ = -11.968, p = 0.0003	n = 6
**Figure 1f**	Before vs. after change point	S+ entry probability	Paired t-test (Holm-Sidak corrected)	$t_{(5)}$ = -6.069, p = 0.0035	n = 6
**Figure 1f**	Before vs. after change point	S+ latency	Paired t-test (Holm-Sidak corrected)	$t_{(5)}$ = 6.849, p = 0.0030	n = 6
**Figure 1f**	Before vs. after change point	ITI pseudolatency	Paired t-test (Holm-Sidak corrected)	$t_{(5)}$ = 0.1855, p = 0.8601	n = 6
**Figure 2c**	S+ vs. S- (-120 to -81 trials from CP)	Firing 100-400 ms after cue (Z sc.)	Wilcoxon signed-rank test (Holm-Sidak corrected)	p = 0.0053	n = 45
**Figure 2c**	S+ vs. S- (-80 to -41 trials from CP)	Firing 100-400 ms after cue (Z sc.)	Wilcoxon signed-rank test (Holm-Sidak corrected)	p = 0.4649	n = 55
**Figure 2c**	S+ vs. S- (-40 to -1 trials from CP)	Firing 100-400 ms after cue (Z sc.)	Wilcoxon signed-rank test (Holm-Sidak corrected)	p = 0.0075	n = 68
**Figure 2c**	S+ vs. S- (0 to 39 trials from CP)	Firing 100-400 ms after cue (Z sc.)	Wilcoxon signed-rank test (Holm-Sidak corrected)	p < 0.00001	n = 63
**Figure 2c**	S+ vs. S- (40 to 79 trials from CP)	Firing 100-400 ms after cue (Z sc.)	Wilcoxon signed-rank test (Holm-Sidak corrected)	p < 0.00001	n = 37
**Figure 2c**	S+ vs. S- (80 to 119 trials from CP)	Firing 100-400 ms after cue (Z sc.)	Wilcoxon signed-rank test (Holm-Sidak corrected)	p = 0.0004	n = 17
**Figure 2c**	-120 to -81 vs.-40 to -1 trials from CP	Firing 100-400 ms after S+ (Z sc.)	Wilcoxon rank sum test (Holm-Sidak corrected)	p = 0.2957	n = 45 / n = 68
**Figure 2c**	-40 to -1 vs. 40 to 79 trials from CP	Firing 100-400 ms after S+ (Z sc.)	Wilcoxon rank sum test (Holm-Sidak corrected)	p = 0.0218	n = 68 / n = 37
**Figure 2d**	-120 to -81 vs.-40 to -1 trials from CP	Proportion of cue-excited neurons	Fisher's exact test for count data (Holm-Sidak corrected)	p = 0.0321	n = 45 / n = 68
**Figure 2d**	-40 to -1 vs. 40 to 79 trials from CP	Proportion of cue-excited neurons	Fisher's exact test for count data (Holm-Sidak corrected)	p = 0.0229	n = 68 / n = 37
**Figure 2d**	-120 to -81 vs.-40 to -1 trials from CP	Proportion of cue-inhibited neurons	Fisher's exact test for count data (Holm-Sidak corrected)	p = 0.3424	n = 45 / n = 68
**Figure 2d**	-40 to -1 vs. 40 to 79 trials from CP	Proportion of cue-inhibited neurons	Fisher's exact test for count data (Holm-Sidak corrected)	p = 0.0153	n = 68 / n = 37
**Figure 2e**	-120 to -81 vs.-40 to -1 trials from CP	Cue-excited units Firing 100-400 ms after S+ (Z sc.)	Wilcoxon rank sum test (Holm-Sidak corrected)	p = 0.0473	n = 17 / n =39
**Figure 2e**	-40 to -1 vs. 40 to 79 trials from CP	Cue-excited units	Wilcoxon rank sum test	p = 0.0473	n = 39 / n = 30

		Firing 100-400 ms after S+ (Z sc.)	(Holm-Sidak corrected)		
**Figure 2f**	Average S+ latency on each session	Average firing 100-400 ms after S+ (Z sc.) of all cue-excited units on each session	Simple linear regression	Without outliers: r = -0.8, $p < 0.0001$; $R^2 = 0.65$; β=-0.85, $p < 0.0001$<br>With outliers: r = -0.8, $p < 0.0001$; $R^2 = 0.63$; β=-0.83, $p < 0.0001$	n = 24
**Figure 2f**	Average S+ entry probability on each session	Average firing 100-400 ms after S+ (Z sc.) of all cue-excited units on each session	Simple linear regression	Without outliers: r = 0.71, $p < 0.001$; $R^2 = 0.50$; β=-5.4, $p < 0.001$<br>With outliers: r = 0.72, $p < 0.0001$; $R^2 = 0.52$; β=6.02, $p < 0.001$	n = 24
**Figure 2f**	Average S+ performance index on each session	Average firing 100-400 ms after S+ (Z sc.) of all cue-excited units on each session	Simple linear regression	Without outliers: r = 0.78, $p < 0.001$; $R^2 = 0.61$; β=-1.01, $p < 0.001$<br>With outliers: r = 0.77, $p < 0.001$; $R^2 = 0.60$; β=0.96, $p < 0.001$	n = 24
**Figure 3b**	*Before change point*<br>Firing 100-400 ms after S+ onset (Z sc.)	*Before change point*<br>Firing 0-1500 ms after S+ entry (Z sc.)	Simple linear regression	Without outliers: r = 0.5, $p < 0.0001$; $R^2 = 0.25$; β=0.62, $p < 0.0001$<br>With outliers: r = 0.46, $p < 0.0001$; $R^2 = 0.21$; β=0.64, $p < 0.0001$	n = 97
**Figure 3b**	*On or after change point session*<br>Firing 100-400 ms after S+ onset (Z sc.)	*On or after change point session*<br>Firing 0-1500 ms after S+ entry (Z sc.)	Simple linear regression	Without outliers: r = -0.38, p = 0.0003; $R^2 = 0.15$; β=-1.29, p = 0.0003<br>With outliers: r = -0.38, p = 0.0002; $R^2 = 0.14$; β=-1.73, p = 0.0002	n = 89
**Figure 4c**	AP5 vs. VEH group<br>30 min bins<br>Moderate training	S+ performance index	Mixed two-factor ANOVA<br>*Between-subject: drug*<br>*Within-subject: time bin*	Drug: $F_{(1, 9)} = 12.119$, p = 0.0069<br>Time: $F_{(1, 9)} = 1.5105$, p = 0.2502<br>Drug x time: $F_{(1, 9)} = 5.111$, p = 0.0500	n = 11
**Figure 4c**	AP5 vs. VEH group<br>1 to 30 min (baseline)<br>Moderate training	S+ performance index	Welch's t-test (Holm-Sidak corrected)	$t_{(22.7)} = 0.304$, p = 0.3819	n = 5 / n = 6
**Figure 4c**	AP5 vs. VEH group<br>31 to 60 min<br>Moderate training	S+ performance index	Welch's t-test (Holm-Sidak corrected)	$t_{(25.93)} = 4.292$, p = 0.0004	n = 5/ n = 6
**Figure 4c**	AP5 vs. VEH group<br>61 to 90 min<br>Moderate training	S+ performance index	Welch's t-test (Holm-Sidak corrected)	$t_{(25.39)} = 4.021$, p = 0.0007	n = 5/ n = 6
**Figure 4c**	AP5 vs. VEH group<br>91 to 120 min<br>Moderate training	S+ performance index	Welch's t-test (Holm-Sidak corrected)	$t_{(27.76)} = 3.553$, p = 0.0013	n = 5/ n = 6
**Figure 4d**	VEH group (S+): before vs. after infusion<br>Moderate training	Firing 100-400 ms after S+ (Z sc.)	Wilcoxon signed-rank test (Holm-Sidak corrected)	p = 0.6406	n = 8
**Figure 4d**	AP5 group (S+): before vs. after infusion<br>Moderate training	Firing 100-400 ms after S+ (Z sc.)	Wilcoxon signed-rank test (Holm-Sidak corrected)	$p < 0.00001$	n = 30
**Figure 4e**	AP5 vs. VEH infusion<br>30 min bins<br>Extended training	S+ performance index	Two-factor rep. measures ANOVA.<br>*Within-subject:*<br>- *Drug*<br>- *Time bin*	Drug: $F_{(1, 4)} = 2.251$, p = 0.6729<br>Time: $F_{(1, 4)} = 0.207$, p = 0.2079<br>Drug x time: $F_{(1, 4)} = 0.211$, p = 0.6701	n = 5
**Figure 4e**	AP5 vs. VEH infusion<br>1 to 30 min (baseline)	S+ performance index	Unpaired t-test (Holm-Sidak corrected)	$t_{(14)} = 0.188$, p = 1	n = 5

	Extended training				
**Figure 4e**	AP5 vs. VEH infusion 31 to 60 min Extended training	S+ performance index	Unpaired t-test (Holm-Sidak corrected)	$t_{(14)}$ = 1.744, p = 0.206	n = 5
**Figure 4e**	AP5 vs. VEH infusion 61 to 90 min Extended training	S+ performance index	Unpaired t-test (Holm-Sidak corrected)	$t_{(14)}$ = -0.314, p = 1	n = 5
**Figure 4e**	AP5 vs. VEH infusion 91 to 120 min Extended training	S+ performance index	Unpaired t-test (Holm-Sidak corrected)	$t_{(14)}$ = 0.139, p = 1	n = 5
**Figure 4f**	VEH infusion (S+): before vs. after infusion Extended training	Firing 100-400 ms after S+ (Z sc.)	Wilcoxon signed-rank test (Holm-Sidak corrected)	p = 1	n =47
**Figure 4f**	AP5 infusion (S+): before vs. after infusion Extended training	Firing 100-400 ms after S+ (Z sc.)	Wilcoxon signed-rank test (Holm-Sidak corrected)	p = 0.2041	n = 59
**Figure 4e**	AP5 vs. VEH	S+ entry probability	Mixed two-factor ANOVA: *Between-subject: drug* *Within-subject: bin*	Drug: $F_{(1, 12)}$=33.26, p < 0.001; Bin: $F_{(1, 12)}$=38.4, p < 0.001; Drug x bin: $F_{(1, 12)}$=22.35, p<.001	n = 14
**Figure 4e**	AP5 vs. VEH during first 5 min	S+ entry probability	Welch's t-test	$t_{(11.55)}$=9.72, p < 0.001	n = 7/ n = 7
**Figure 5f**	AP5 vs. VEH	S+ performance index	Mixed two-factor ANOVA: *Between-subject: drug* *Within-subject: bin*	Drug: $F_{(1, 12)}$=24.11, p < 0.001; Bin: $F_{(1, 12)}$=1.11, p = 0.31; Drug x bin: $F_{(1, 12)}$=10.25, p=.007	n = 14
**Figure 5f**	AP5 vs. VEH during first 5 min	S+ performance index	Welch's t-test	$t_{(11.47)}$ = 5.41, p < 0.001	n = 7/ n = 7
**Figure 6c**	Before vs. after change point	S+ performance index	Paired t-test (Holm-Sidak corrected)	$t_{(10\ )}$= -10.21, p < 0.00001	n = 11
**Figure 6c**	Before vs. after change point	S+	Paired t-test (Holm-Sidak corrected)	$t_{(10)}$ = -5.061., p = 0.001	n = 11
**Figure 6c**	Before vs. after change point	S+ latency	Paired t-test (Holm-Sidak corrected)	$t_{(10)}$= 5.938, p = 0.0004	n = 11
**Figure 6c**	Before vs. after change point	ITI pseudolatency	Paired t-test (Holm-Sidak corrected)	$t_{(10)}$= 1.245, p = 0.241	n = 11
**Figure 7b**	VEH side: S+ vs. S- (-105 to -71 trials from CP)	Firing 100-400 ms after cue (Z sc.)	Wilcoxon signed-rank test (Holm-Sidak corrected)	p = 0.3360	n = 75
**Figure 7b**	VEH side: S+ vs. S- (-70 to -36 trials from CP)	Firing 100-400 ms after cue (Z sc.)	Wilcoxon signed-rank test (Holm-Sidak corrected)	p < 0.0001	n = 137
**Figure 7b**	VEH side: S+ vs. S- (-35 to -1 trials from CP)	Firing 100-400 ms after cue (Z sc.)	Wilcoxon signed-rank test (Holm-Sidak corrected)	p < 0.0001	n = 129
**Figure 7b**	VEH side: S+ vs. S- (0 to 34 trials from CP)	Firing 100-400 ms after cue (Z sc.)	Wilcoxon signed-rank test (Holm-Sidak corrected)	p < 0.0001	n = 131
**Figure 7b**	VEH side: S+ vs. S- (35 to 69 trials from CP)	Firing 100-400 ms after cue (Z sc.)	Wilcoxon signed-rank test (Holm-Sidak corrected)	p < 0.0001	n = 92
**Figure 7b**	VEH side: S+ vs. S-	Firing 100-400 ms after cue (Z sc.)	Wilcoxon signed-rank test	p < 0.0001	n = 36

	(70 to 104 trials from CP)		(Holm-Sidak corrected)		
**Figure 7b**	VEH side: bin 1 vs. 3 (-105 to -71 vs. -35 to -1 trials from CP)	Firing 100-400 ms after cue (Z sc.)	Wilcoxon rank sum test (Holm-Sidak corrected)	p=0.0019	n = 75/ n = 129
**Figure 7b**	VEH side: bin 3 vs. 5 (-35 to -1 vs. 35 to 69 trials from CP)	Firing 100-400 ms after cue (Z sc.)	Wilcoxon rank sum test (Holm-Sidak corrected)	p=0.0013	n = 129/ n = 92
**Figure 7b**	AP5 side: S+ vs. S- (-105 to -71 trials from CP)	Firing 100-400 ms after cue (Z sc.)	Wilcoxon signed-rank test (Holm-Sidak corrected)	p = 0.0004	n = 66
**Figure 7b**	AP5 side: S+ vs. S- (-70 to -36 trials from CP)	Firing 100-400 ms after cue (Z sc.)	Wilcoxon signed-rank test (Holm-Sidak corrected)	p < 0.0001	n = 103
**Figure 7b**	AP5 side: S+ vs. S- (-35 to -1 trials from CP)	Firing 100-400 ms after cue (Z sc.)	Wilcoxon signed-rank test (Holm-Sidak corrected)	p < 0.0001	n = 96
**Figure 7b**	AP5 side: S+ vs. S- (0 to 34 trials from CP)	Firing 100-400 ms after cue (Z sc.)	Wilcoxon signed-rank test (Holm-Sidak corrected)	p < 0.0001	n = 102
**Figure 7b**	AP5 side: S+ vs. S- (35 to 69 trials from CP)	Firing 100-400 ms after cue (Z sc.)	Wilcoxon signed-rank test (Holm-Sidak corrected)	p < 0.0001	n = 85
**Figure 7b**	AP5 side: S+ vs. S- (70 to 104 trials from CP)	Firing 100-400 ms after cue (Z sc.)	Wilcoxon signed-rank test (Holm-Sidak corrected)	p < 0.0001	n = 75
**Figure 7b**	AP5 side: bin 1 vs. 3 (-105 to -71 vs. -35 to -1 trials from CP)	Firing 100-400 ms after cue (Z sc.)	Wilcoxon rank sum test (Holm-Sidak corrected)	p=0.0071	n = 66/ n = 96
**Figure 7b**	AP5 side: bin 3 vs. 5 (-35 to -1 vs. 35 to 69 trials from CP)	Firing 100-400 ms after cue (Z sc.)	Wilcoxon rank sum test (Holm-Sidak corrected)	p=0.7631	n = 96/ n = 75
**Figure 7c**	S+: VEH vs. AP5 side (-105 to -71 trials from CP)	Firing 100-400 ms after cue (Z sc.)	Wilcoxon rank sum test (Holm-Sidak corrected)	p = 0.0839	n = 75/ n = 66
**Figure 7c**	S+: VEH vs. AP5 side (-70 to -36 trials from CP)	Firing 100-400 ms after cue (Z sc.)	Wilcoxon rank sum test (Holm-Sidak corrected)	p = 0.5941	n = 137/ n = 103
**Figure 7c**	S+: VEH vs. AP5 side (-35 to -1 trials from CP)	Firing 100-400 ms after cue (Z sc.)	Wilcoxon rank sum test (Holm-Sidak corrected)	p = 0.5941	n = 129/ n = 96
**Figure 7c**	S+: VEH vs. AP5 side (0 to 34 trials from CP)	Firing 100-400 ms after cue (Z sc.)	Wilcoxon rank sum test (Holm-Sidak corrected)	p = 0.0181	n = 131/ n = 102
**Figure 7c**	S+: VEH vs. AP5 side (35 to 69 trials from CP)	Firing 100-400 ms after cue (Z sc.)	Wilcoxon rank sum test (Holm-Sidak corrected)	p = 0.0008	n = 92/ n = 85
**Figure 7c**	S+: VEH vs. AP5 side (70 to 104 trials from CP)	Firing 100-400 ms after cue (Z sc.)	Wilcoxon rank sum test (Holm-Sidak corrected)	p = 0.0364	n = 36/ n = 75
**Figure 7c**	S+: VEH vs. AP5 side	Firing 750-2000 ms after cue (Z sc.)	Wilcoxon rank sum test	p = 0.9541	n = 75/ n = 66

	(-105 to -71 trials from CP)		(Holm-Sidak corrected)		
**Figure 7c**	S+: VEH vs. AP5 side (-70 to -36 trials from CP)	Firing 750-2000 ms after cue (Z sc.)	Wilcoxon rank sum test (Holm-Sidak corrected)	p = 0.9541	n = 137/ n = 103
**Figure 7c**	S+: VEH vs. AP5 side (-35 to -1 trials from CP)	Firing 750-2000 ms after cue (Z sc.)	Wilcoxon rank sum test (Holm-Sidak corrected)	p = 0.1461	n = 129/ n = 96
**Figure 7c**	S+: VEH vs. AP5 side (0 to 34 trials from CP)	Firing 750-2000 ms after cue (Z sc.)	Wilcoxon rank sum test (Holm-Sidak corrected)	p < 0.0001	n = 131/ n = 102
**Figure 7c**	S+: VEH vs. AP5 side (35 to 69 trials from CP)	Firing 750-2000 ms after cue (Z sc.)	Wilcoxon rank sum test (Holm-Sidak corrected)	p = 0.0002	n = 92/ n = 85
**Figure 7c**	S+: VEH vs. AP5 side (70 to 104 trials from CP)	Firing 750-2000 ms after cue (Z sc.)	Wilcoxon rank sum test (Holm-Sidak corrected)	p < 0.0001	n = 36/ n = 75
**Figure 7d**	VEH side: trial bins 1 vs. 3 (-105 to -71 vs. -35 to -1 trials from CP)	Proportion of cue-excited neurons	Fisher's exact test for count data (Holm-Sidak corrected)	p = 0.0014	n = 75/ n = 129
**Figure 7d**	VEH side: trial bins 3 vs. 5 (-35 to -1 vs. 35 to 69 trials from CP)	Proportion of cue-excited neurons	Fisher's exact test for count data (Holm-Sidak corrected)	p = 0.0004	n = 129/ n = 92
**Figure 7d**	VEH side: trial bins 1 vs. 3 (-105 to -71 vs. -35 to -1 trials from CP)	Proportion of cue-inhibited neurons	Fisher's exact test for count data (Holm-Sidak corrected)	p = 0.0006	n = 75/ n = 129
**Figure 7d**	VEH side: trial bins 3 vs. 5 (-35 to -1 vs. 35 to 69 trials from CP)	Proportion of cue-inhibited neurons	Fisher's exact test for count data (Holm-Sidak corrected)	p = 0.3471	n = 129/ n = 92
**Figure 7d**	AP5 side: trial bins 1 vs. 3 (-105 to -71 vs. -35 to -1 trials from CP)	Proportion of cue-excited neurons	Fisher's exact test for count data (Holm-Sidak corrected)	p = 0.2403	n = 66/ n = 96
**Figure 7d**	AP5 side: trial bins 3 vs. 5 (-35 to -1 vs. 35 to 69 trials from CP)	Proportion of cue-excited neurons	Fisher's exact test for count data (Holm-Sidak corrected)	p = 0.2403	n = 96/ n = 75
**Figure 7d**	AP5 side: trial bins 1 vs. 3 (-105 to -71 vs. -35 to -1 trials from CP)	Proportion of cue-inhibited neurons	Fisher's exact test for count data (Holm-Sidak corrected)	p = 0.0689	n = 66/ n = 96
**Figure 7d**	AP5 side: trial bins 3 vs. 5 (-35 to -1 vs. 35 to 69 trials from CP)	Proportion of cue-inhibited neurons	Fisher's exact test for count data (Holm-Sidak corrected)	p = 0.5356	n = 96/ n = 75
**Figure 7d**	VEH vs. AP5 sides (-105 vs. -71 from CP)	Proportion of cue-excited neurons	Fisher's exact test for count data (Holm-Sidak corrected)	p = 0.7807	n = 75/ n = 66
**Figure 7d**	VEH vs. AP5 sides (-70 vs. -36 from CP)	Proportion of cue-excited neurons	Fisher's exact test for count data (Holm-Sidak corrected)	p = 0.1395	n = 137/ n = 103
**Figure 7d**	VEH vs. AP5 sides (-35 vs. -1 from CP)	Proportion of cue-excited neurons	Fisher's exact test for count data (Holm-Sidak corrected)	p = 0.0363	n = 129/ n = 96
**Figure 7d**	VEH vs. AP5 sides (0 vs. 34 from CP)	Proportion of cue-excited neurons	Fisher's exact test for count data	p = 0.0009	n = 131/ n = 102

			(Holm-Sidak corrected)		
**Figure 7d**	VEH vs. AP5 sides (35 vs. 69 from CP)	Proportion of cue-excited neurons	Fisher's exact test for count data (Holm-Sidak corrected)	p = 0.0002	n = 92/ n = 85
**Figure 7d**	VEH vs. AP5 sides (70 vs. 105 from CP)	Proportion of cue-excited neurons	Fisher's exact test for count data (Holm-Sidak corrected)	p =0.0460	n = 36/ n = 75
**Figure 7d**	VEH vs. AP5 sides (-105 vs. -71 from CP)	Proportion of cue-inhibited neurons	Fisher's exact test for count data (Holm-Sidak corrected)	p = 0.7076	n = 75/ n = 66
**Figure 7d**	VEH vs. AP5 sides (-70 vs. -36 from CP)	Proportion of cue-inhibited neurons	Fisher's exact test for count data (Holm-Sidak corrected)	p = 0.7076	n = 137/ n = 103
**Figure 7d**	VEH vs. AP5 sides (-35 vs. -1 from CP)	Proportion of cue-inhibited neurons	Fisher's exact test for count data (Holm-Sidak corrected)	p = 0.7076	n = 129/ n = 96
**Figure 7d**	VEH vs. AP5 sides (0 vs. 34 from CP)	Proportion of cue-inhibited neurons	Fisher's exact test for count data (Holm-Sidak corrected)	p = 0.6126	n = 131/ n = 102
**Figure 7d**	VEH vs. AP5 sides (35 vs. 69 from CP)	Proportion of cue-inhibited neurons	Fisher's exact test for count data (Holm-Sidak corrected)	p = 0.6126	n = 92/ n = 85
**Figure 7d**	VEH vs. AP5 sides (70 vs. 105 from CP)	Proportion of cue-inhibited neurons	Fisher's exact test for count data (Holm-Sidak corrected)	p = 0.7076	n = 36/ n = 75
**Figure 7f**	S+: VEH vs. AP5 side (-105 to -71 trials from CP)	Firing 100-400 ms after cue (Z sc.) Cue-excited units	Wilcoxon rank sum test (Holm-Sidak corrected)	p = 0.4073	n = 19/ n = 14
**Figure 7f**	S+: VEH vs. AP5 side (-70 to -36 trials from CP)	Firing 100-400 ms after cue (Z sc.) Cue-excited units	Wilcoxon rank sum test (Holm-Sidak corrected)	p = 0.4073	n = 45/ n = 24
**Figure 7f**	S+: VEH vs. AP5 side (-35 to -1 trials from CP)	Firing 100-400 ms after cue (Z sc.) Cue-excited units	Wilcoxon rank sum test (Holm-Sidak corrected)	p = 0.4835	n = 63/ n = 31
**Figure 7f**	S+: VEH vs. AP5 side (0 to 34 trials from CP)	Firing 100-400 ms after cue (Z sc.) Cue-excited units	Wilcoxon rank sum test (Holm-Sidak corrected)	p = 0.4835	n = 87/ n = 43
**Figure 7f**	S+: VEH vs. AP5 side (35 to 69 trials from CP)	Firing 100-400 ms after cue (Z sc.) Cue-excited units	Wilcoxon rank sum test (Holm-Sidak corrected)	p = 0.4835	n = 56/ n = 37
**Figure 7f**	S+: VEH vs. AP5 side (70 to 104 trials from CP)	Firing 100-400 ms after cue (Z sc.) Cue-excited units	Wilcoxon rank sum test (Holm-Sidak corrected)	p = 0.2173	n = 23/ n = 36
**Figure 7f**	S+: VEH vs. AP5 side (-105 to -71 trials from CP)	Firing 750-2000 ms after cue (Z sc.) Cue-excited units	Wilcoxon rank sum test (Holm-Sidak corrected)	p = 0.5289	n = 19/ n = 14
**Figure 7f**	S+: VEH vs. AP5 side (-70 to -36 trials from CP)	Firing 750-2000 ms after cue (Z sc.) Cue-excited units	Wilcoxon rank sum test (Holm-Sidak corrected)	p = 0.4427	n = 45/ n = 24
**Figure 7f**	S+: VEH vs. AP5 side	Firing 750-2000 ms after cue (Z sc.) Cue-excited units	Wilcoxon rank sum test	p = 0.5289	n = 63/ n = 31

	(-35 to -1 trials from CP)		(Holm-Sidak corrected)		
**Figure 7f**	S+: VEH vs. AP5 side<br>(0 to 34 trials from CP)	Firing 750-2000 ms after cue (Z sc.)<br>Cue-excited units	Wilcoxon rank sum test<br>(Holm-Sidak corrected)	p = 0.0162	n = 87/<br>n = 43
**Figure 7f**	S+: VEH vs. AP5 side<br>(35 to 69 trials from CP)	Firing 750-2000 ms after cue (Z sc.)<br>Cue-excited units	Wilcoxon rank sum test<br>(Holm-Sidak corrected)	p = 0.0049	n = 56/<br>n = 37
**Figure 7f**	S+: VEH vs. AP5 side<br>(70 to 104 trials from CP)	Firing 750-2000 ms after cue (Z sc.)<br>Cue-excited units	Wilcoxon rank sum test<br>(Holm-Sidak corrected)	p = 0.0002	n = 23/<br>n = 36
**Figure 8a**	S+ vs. S-<br>5 trial bin<br>"Learners"	Performance index	Two-factor rep. measures ANOVA.<br>*Within-subject:*<br>- *Cue*<br>- *Bin.*	Cue: $F_{(1, 5)}$ = 119.926, p = .0001<br>Bin: $F_{(1.06, 5.3)}$= 2.38, p = 0.1170<br>Cue x bin: $F_{(0.86, 3.35)}$=10.527, p=0.0023	n = 5
**Figure 8b**	VEH vs. AP5 side "Learners"	Proportion of cue-excited neurons	Fisher's exact test for count data	p = 0.3073	n = 38/<br>n = 39
**Figure 8c**	VEH vs. AP5 side "Learners"	Firing 100-400 ms after S+ (Z sc.)	Wilcoxon rank sum test<br>(Holm-Sidak corrected)	p < 0.0001	n = 38/<br>n = 39
**Figure 8c**	VEH vs. AP5 side "Learners"	Firing 750-2000 ms after S+ (Z sc.)	Wilcoxon rank sum test<br>(Holm-Sidak corrected)	p < 0.0001	n = 38/<br>n = 39
**Figure 8c**	VEH vs. AP5 side "Learners"	Firing 100-400 ms after S+ (Z sc.)<br>Cue-excited units	Wilcoxon rank sum test<br>(Holm-Sidak corrected)	p < 0.0001	n = 38/<br>n = 39
**Figure 8c**	VEH vs. AP5 side "Learners"	Firing 750-2000 ms after S+ (Z sc.)<br>Cue-excited units	Wilcoxon rank sum test<br>(Holm-Sidak corrected)	p < 0.0001	n = 38/<br>n = 39

B. Statistical tests in supplementary figures

Figure	Independent variable(s)	Dependent variable	Test	Result	Sample size
**S4a**	S+ vs. S-<br>Session (1 to 6)	Firing 100-400 ms after cue (Z sc.)	Mixed two-factor ANOVA<br>*Between-subject: session*<br>*Within-subject: kind of cue*	Session: $F_{(5, 358)}$ = 4.87, p = 0.0003<br>Cue: $F_{(1, 358)}$ = 70.642, p <0.00001<br>Session x Cue: $F_{(5, 358)}$ = 3.889, p = 0.0019	n = 186
**S4a**	S+ vs. S-<br>Session 1	Firing 100-400 ms after cue (Z sc.)	Wilcoxon signed-rank test (Holm-Sidak corrected)	p = 0.0013	n = 35
**S4a**	S+ vs. S-<br>Session 2	Firing 100-400 ms after cue (Z sc.)	Wilcoxon signed-rank test (Holm-Sidak corrected)	p = 0.0146	n = 32
**S4a**	S+ vs. S-<br>Session 3	Firing 100-400 ms after cue (Z sc.)	Wilcoxon signed-rank test (Holm-Sidak corrected)	p = 0.4025	n = 32
**S4a**	S+ vs. S-<br>Session 4	Firing 100-400 ms after cue (Z sc.)	Wilcoxon signed-rank test (Holm-Sidak corrected)	p = 0.0002	n = 39
**S4a**	S+ vs. S-<br>Session 5	Firing 100-400 ms after cue (Z sc.)	Wilcoxon signed-rank test (Holm-Sidak corrected)	p < 0.0001	n = 22

**S4a**	S+ vs. S-<br>Session 6	Firing 100-400 ms after cue (Z sc.)	Wilcoxon signed-rank test (Holm-Sidak corrected)	$p < 0.0001$	n = 26
**S4b**	S+ vs S-<br>Session 1. Trials 1-10.	Firing 100-400 ms after cue (Z sc.)	Wilcoxon signed-rank test (Holm-Sidak corrected)	$p = 0.3320$	n = 35
**S4b**	S+ vs S-<br>Session 1. Trials 11-20.	Firing 100-400 ms after cue (Z sc.)	Wilcoxon signed-rank test (Holm-Sidak corrected)	$p = 0.0400$	n = 35
**S4b**	S+ vs S-<br>Session 1. Trials 21-30.	Firing 100-400 ms after cue (Z sc.)	Wilcoxon signed-rank test (Holm-Sidak corrected)	$p = 0.0076$	n = 35
**S4b**	S+ vs S-<br>Session 1. Trials 31-40.	Firing 100-400 ms after cue (Z sc.)	Wilcoxon signed-rank test (Holm-Sidak corrected)	$p = 0.0081$	n = 35
**S4c**	S+ sensory modality (light vs. tone)<br>Before vs. After CP	Firing 100-400 ms after S+ (Z sc.)	Two-factor ANOVA<br>*Between-subject:*<br>- *S+ modality*<br>- *Pre/post CP*	S+ modality: $F_{(1, 145)} = 0.006$,<br>$p = 0.9403$<br>Pre/post CP: $F_{(1, 145)} = 29.355$,<br>$p < 0.0001$<br>Interaction: $F_{(1, 145)} = 0.0778$,<br>$p = 0.7805$	n = 149
**S4c**	Light S+: pre vs. post CP	Firing 100-400 ms after S+ (Z sc.)	Wilcoxon rank sum test<br>(Holm-Sidak corrected)	$p < 0.0001$	n = 70/ n= 6
**S4 c**	Tone S+: pre vs. post CP	Firing 100-400 ms after S+ (Z sc.)	Wilcoxon rank sum test<br>(Holm-Sidak corrected)	$p = 0.0247$	n = 27/ n = 46
**S4c**	Pre CP: tone vs. light S+	Firing 100-400 ms after S+ (Z sc.)	Wilcoxon rank sum test<br>(Holm-Sidak corrected)	$p = 0.2718$	n = 27/ n =70
**S4c**	Post CP: tone vs. light S+	Firing 100-400 ms after S+ (Z sc.)	Wilcoxon rank sum test<br>(Holm-Sidak corrected)	$p = 0.5278$	n = 46/ n = 6
**S4d**	S+ vs. S-<br>(-120 to -81 trials from CP)	Firing 750-2000 ms after cue (Z sc.)	Wilcoxon signed-rank test (Holm-Sidak corrected)	$p = 0.2815$	n = 45
**S4d**	S+ vs. S-<br>(-80 to -41 trials from CP)	Firing 750-2000 ms after cue (Z sc.)	Wilcoxon signed-rank test (Holm-Sidak corrected)	$p = 0.2815$	n = 55
**S4d**	S+ vs. S-<br>(-40 to -1 trials from CP)	Firing 750-2000 ms after cue (Z sc.)	Wilcoxon signed-rank test (Holm-Sidak corrected)	$p < 0.0001$	n = 68
**S4d**	S+ vs. S-<br>(0 to 39 trials from CP)	Firing 750-2000 ms after cue (Z sc.)	Wilcoxon signed-rank test (Holm-Sidak corrected)	$p < 0.0001$	n = 63
**S4d**	S+ vs. S-<br>(40 to 79 trials from CP)	Firing 750-2000 ms after cue (Z sc.)	Wilcoxon signed-rank test (Holm-Sidak corrected)	$p = 0.0013$	n = 37
**S4d**	S+ vs. S-<br>(80 to 119 trials from CP)	Firing 750-2000 ms after cue (Z sc.)	Wilcoxon signed-rank test (Holm-Sidak corrected)	$p = 0.0034$	n = 17
**S4d**	Trial bins 1 vs. 3<br>(-120 to -81 vs.-40 to 0 trials from CP)	Firing 750-2000 ms after S+ (Z sc.)	Wilcoxon rank sum test<br>(Holm-Sidak corrected)	$p = 0.1151$	n = 45 /<br>n = 68
**S4d**	Trial bins 3 vs. 5<br>(-40 to -1 vs. 40 to 79 trials from CP)	Firing 750-2000 ms after S+ (Z sc.)	Wilcoxon rank sum test<br>(Holm-Sidak corrected)	$p = 0.086$	n = 68 /<br>n = 37
**S4d**	Trial bins 2 vs. 4<br>(-80 to -41 vs. 0 to 39 trials from CP)	Firing 750-2000 ms after S+ (Z sc.)	Wilcoxon rank sum test	$p < 0.0001$	n = 55 /<br>n = 63

			(Holm-Sidak corrected)		
**S4d**	Trial bins 4 vs. 6 (0 to 39 vs. 80 to 119 trials from CP)	Firing 750-2000 ms after S+ (Z sc.)	Wilcoxon rank sum test (Holm-Sidak corrected)	$p = 0.0481$	n = 63 / n = 17
**S4e**	S+ vs. S- (-120 to -81 trials from CP)	Firing -2000-0 ms (Z sc.) before entry (when latency > 5 s)	Wilcoxon rank sum test (Holm-Sidak corrected)	$p = 0.9989$	n = 42/ n = 31
**S4e**	S+ vs. S- (-80 to -41 trials from CP)	Firing -2000-0 ms (Z sc.) before entry (when latency > 5 s)	Wilcoxon rank sum test (Holm-Sidak corrected)	$p = 0.6142$	n = 38/ n = 51
**S4e**	S+ vs. S- (-40 to -1 trials from CP)	Firing -2000-0 ms (Z sc.) before entry (when latency > 5 s)	Wilcoxon rank sum test (Holm-Sidak corrected)	$p = 0.0285$	n = 68/ n = 47
**S4e**	S+ vs. S- (0 to 39 trials from CP)	Firing -2000-0 ms (Z sc.) before entry (when latency > 5 s)	Wilcoxon rank sum test (Holm-Sidak corrected)	$p = 0.0177$	n = 63/ n = 63
**S4e**	S+ vs. S- (40 to 79 trials from CP)	Firing -2000-0 ms (Z sc.) before entry (when latency > 5 s)	Wilcoxon rank sum test (Holm-Sidak corrected)	$p = 0.029$	n = 12/ n = 22
**S4e**	S+ vs. S- (80 to 119 trials from CP)	Firing -2000-0 ms (Z sc.) before entry (when latency > 5 s)	Wilcoxon rank sum test (Holm-Sidak corrected)	$p = 0.0062$	n = 11/ n = 17
**S4e**	Trial bins 1 vs. 3 (-120 to -81 vs.-40 to 0 trials from CP)	Firing -2000-0 ms (Z sc.) before entry (when latency > 5 s)	Wilcoxon rank sum test (Holm-Sidak corrected)	$p = 0.0017$	n = 42/ n = 68
**S4e**	Trial bins 3 vs. 5 (-40 to -1 vs. 40 to 79 trials from CP)	Firing -2000-0 ms (Z sc.) before entry (when latency > 5 s)	Wilcoxon rank sum test (Holm-Sidak corrected)	$p = 0.0206$	n = 68/ n = 12
**S5b**	*Before change point session.* Average firing 100-400 ms after S+ onset (Z sc.).	*Before change point session.* Average firing 0-1500 ms after S- entry (Z sc.).	Simple linear regression	Without outliers: $r = 0.22$, $p = 0.0582$; $R^2 = 0.05$; $\beta=0.64$, $p = 0.0542$ With outliers: $r = 0.18$, $p = 0.0843$; $R^2 = 0.03$; $\beta=0.64$, $p = 0.0843$	n = 97
**S5b**	*Before change point session.* Average firing 100-400 ms after S+ onset (Z sc.).	*Before change point session.* Average firing 0-1500 ms after ITI entry (Z sc.).	Simple linear regression	Without outliers: $r = -0.09$, $p = 0.3993$; $R^2 = 0.01$; $\beta=-0.15$, $p = 0.3993$ With outliers: $r = 0.02$, $p = 0.8291$; $R^2 = 0$; $\beta=0.04$, $p = 0.8291$	n = 97
**S5c**	*On or after change point session.* Average firing 100-400 ms after S+ onset (Z sc.).	*On or after change point session.* Average firing 0-1500 ms after S- entry (Z sc.).	Simple linear regression	Without outliers: $r = 0.19$, $p = 0.0905$; $R^2 = 0.03$; $\beta=1.5$, $p = 0.0905$ With outliers: $r = 0.06$, $p = 0.0843$; $R^2 = 0$; $\beta=0.65$, $p = 0.5991$	n = 89
**S5c**	*On or after change point session.* Average firing 100-400 ms after S+ onset (Z sc.).	*On or after change point session.* Average firing 0-1500 ms after ITI entry (Z sc.).	Simple linear regression	Without outliers: $r = -0.07$, $p = 0.5357$; $R^2 = 0$; $\beta=-0.2$, $p = 0.5357$ With outliers: $r = -0.13$, $p = 0.2315$; $R^2 = 0.02$; $\beta=-0.61$, $p = 0.2315$	n = 89
**S6a**	Before vs. after change point	S+ performance index	Paired t-test (Holm-Sidak corrected)	$t_{(7)} = -6.84$, $p < 0.001$	n = 8
**S6a**	Before vs. after change point	S+ entry probability	Paired t-test	$t_{(7)} = -3.9$, $p = 0.0059$	n = 8

			(Holm-Sidak corrected)		
**S6a**	Before vs. after change point	S+ latency	Paired t-test (Holm-Sidak corrected)	$t_{(7)} = 5.24$, $p = 0.0018$	n = 8
**S6a**	Before vs. after change point	ITI pseudolatency	Paired t-test (Holm-Sidak corrected)	$t_{(7)} = -1.72$, $p = 0.0641$	n = 8
**S6b**	Pre CP: driving arrays down vs. not driving arrays down	Firing 100-400 ms after S+ (Z sc.)<br>Cue-excited units	Wilcoxon rank sum test (Holm-Sidak corrected)	$p = 0.1319$	n = 36/<br>n = 130
**S6b**	Post CP: driving arrays down vs. not driving arrays down	Firing 100-400 ms after S+ (Z sc.)<br>Cue-excited units	Wilcoxon rank sum test (Holm-Sidak corrected)	$p = 0.1326$	n = 42/<br>n = 78
**S6b**	Driving arrays down: pre CP vs. post CP	Firing 100-400 ms after S+ (Z sc.)<br>Cue-excited units	Wilcoxon rank sum test (Holm-Sidak corrected)	$p < 0.0001$	n = 36/<br>n = 78
**S6c**	S+ vs. S- firing. Channel average, day before CP. Not driving arrays down.	Firing 100-400 ms after cue (Z sc.)	Wilcoxon signed-rank test (Holm-Sidak corrected)	$p = 0.0041$	n = 15 channels (5 subjects)
**S6c**	S+ vs. S- firing. Channel average, day after CP. Not driving arrays down.	Firing 100-400 ms after cue (Z sc.)	Wilcoxon signed-rank test (Holm-Sidak corrected)	$p = 0.0006$	n = 15 channels (5 subjects)
**S6c**	S+ firing. Channel average. Day before vs. after CP.<br>Not driving arrays down.	Firing 100-400 ms after cue (Z sc.)	Wilcoxon signed-rank test (Holm-Sidak corrected)	$p < 0.0001$	n = 15 channels (5 subjects)
**S7d**	S+ vs. S-<br>(Trial 1 to 35)	Firing 100-400 ms after cue (Z sc.)	Wilcoxon signed-rank test (Holm-Sidak corrected)	$p = 0.0027$	n = 61
**S7d**	S+ vs. S-<br>(Trial 36 to 70)	Firing 100-400 ms after cue (Z sc.)	Wilcoxon signed-rank test (Holm-Sidak corrected)	$p = 0.958$	n = 57
**S7d**	S+ vs. S-<br>(Trial 71 to 105)	Firing 100-400 ms after cue (Z sc.)	Wilcoxon signed-rank test (Holm-Sidak corrected)	$p = 0.7367$	n = 35
**S7d**	S+ vs. S-<br>(Trial 106 to 140)	Firing 100-400 ms after cue (Z sc.)	Wilcoxon signed-rank test (Holm-Sidak corrected)	$p = 0.7471$	n =19
**S7d**	S+ vs. S-<br>(Trial 141 to 175)	Firing 100-400 ms after cue (Z sc.)	Wilcoxon signed-rank test (Holm-Sidak corrected)	$p = 0.9580$	n = 15
**S7d**	S+ vs. S-<br>(Trial 176 to 210)	Firing 100-400 ms after cue (Z sc.)	Wilcoxon signed-rank test (Holm-Sidak corrected)	$p = 0.9580$	n = 8
**S7e**	% excited vs. % non-excited by bin (≡ session)	Percentage of cue-excited neurons x bin	Fisher’s exact test for count data	$p = 0.2718$	n = 195
**S7e**	% inhibited vs. % non-inhibited by bin (≡ session)	Percentage of cue-inhibited neurons x bin	Fisher’s exact test for count data	$p = 0.9478$	n = 195
**S8a**	AP5 vs. VEH group<br>Pre vs. post infusion<br>Moderate training	S+ entry probability	Mixed two-factor ANOVA:<br>*Between-subject: drug*<br>*Within-subject: pre/post inf.*	Drug: $F_{(1,9)} = 50.991$, $p < 0.0001$<br>Pre/post infusion: $F_{(1, 9)} = 14.724$, $p = 0.004$<br>Interaction: $F_{(1, 9)} = 15.224$, $p = 0.0036$	n = 6/ n = 5

**S8a**	VEH group: pre vs. post infusion. Moderate training.	S+ entry probability	Paired t-test (Holm-Sidak corrected)	$t_{(5)}$=-0.445, p = 0.6624	n = 6/ n = 5
**S8a**	AP5 group: pre vs. post infusion. Moderate training.	S+ entry probability	Paired t-test (Holm-Sidak corrected)	$t_{(4)}$=-3.504, p = 0.0248	n = 6/ n = 5
**S8a**	AP5 vs. VEH group<br>Pre vs. post infusion<br>Moderate training	S- entry probability	Mixed two-factor ANOVA:<br>*Between-subject: drug*<br>*Within-subject: pre/post inf.*	Drug: $F_{(1,9)}$ = 0.517, p = 0.4905<br>Pre/post infusion: $F_{(1, 9)}$ = 4.279, p = 0.068<br>Interaction: $F_{(1, 9)}$ = 2.836,<br>p = 0.1264	n = 6/ n = 5
**S8b**	AP5 vs. VEH group<br>Pre vs. post infusion<br>Moderate training	S+ latency	Mixed two-factor ANOVA:<br>*Between-subject: drug*<br>*Within-subject: pre/post inf.*	Drug: $F_{(1,9)}$ = 88.274, p < 0.0001<br>Pre/post infusion: : $F_{(1,9)}$ = 11.002, p = 0.009<br>Interaction: $F_{(1, 9)}$ = 12.038,<br>p = 0.007	n = 6/ n = 5
**S8b**	VEH group: pre vs. post infusion. Moderate training.	S+ latency	Paired t-test (Holm-Sidak corrected)	$t_{(5)}$=0.709, p = 0.7450	n = 6/ n = 5
**S8b**	AP5 group: pre vs. post infusion. Moderate training.	S+ latency	Paired t-test (Holm-Sidak corrected)	$t_{(4)}$=-3.085, p = 0.0367	n = 6/ n = 5
**S8b**	AP5 vs. VEH group<br>Pre vs. post infusion<br>Moderate training	S- latency	Mixed two-factor ANOVA:<br>*Between-subject: drug*<br>*Within-subject: pre/post inf.*	Drug: $F_{(1,9)}$ = 0.054, p = 0.8217<br>Pre/post infusion: : $F_{(1,9)}$ = 4.408, p = 0.0651<br>Interaction: $F_{(1, 9)}$ = 2.7, p = 0.14	n = 6/ n = 5
**S8c**	AP5 vs. VEH group<br>Pre vs. post infusion<br>Moderate training	ITI pseudolatency	Mixed two-factor ANOVA:<br>*Between-subject: drug*<br>*Within-subject: pre/post inf.*	Drug: $F_{(1,9)}$ = 6.241, p = 0.0339<br>Pre/post infusion: : $F_{(1,9)}$ = 5.825, p = 0.0390<br>Interaction: $F_{(1, 9)}$ = 9.284, p = 0.0139	n = 6/ n = 5
**S8c**	VEH group: pre vs. post infusion. Moderate training.	ITI pseudolatency	Paired t-test (Holm-Sidak corrected)	$t_{(5)}$=-0.299, p = 0.3881	n = 6/ n = 5
**S8c**	AP5 group: pre vs. post infusion. Moderate training.	ITI pseudolatency	Paired t-test (Holm-Sidak corrected)	$t_{(4)}$=-2.916, p = 0.0434	n = 6/ n = 5
**S8d**	AP5 vs. VEH infusion.<br>Pre vs. post infusion<br>Extended training	S+ entry probability	Two-factor rep. measures ANOVA.<br>*Within-subject:*<br>- *Drug*<br>- *Pre/post inf.*	Drug: $F_{(1,4)}$ = 0.4490, p = 0.5395<br>Pre/post infusion: $F_{(1, 4)}$ = 5.943, p = 0.0713<br>Interaction: $F_{(1, 4)}$ = 0.4490,<br>p = 0.5395	n = 5
**S8d**	AP5 vs. VEH infusion.<br>Pre vs. post infusion<br>Extended training	S- entry probability	Two-factor rep. measures ANOVA.<br>*Within-subject:*<br>- *Drug*<br>- *Pre/post inf.*	Drug: $F_{(1, 4)}$ = 0.062, p = 0.8161<br>Pre/post infusion: $F_{(1,4)}$ = 0.859, p = 0.4063<br>Interaction: $F_{(1, 4)}$ = 0.033,<br>p = 0.8640	n = 5
**S8e**	AP5 vs. VEH infusion.<br>Pre vs. post infusion<br>Extended training	S+ latency	Two-factor rep. measures ANOVA.<br>*Within-subject:*<br>- *Drug*<br>- *Pre/post inf.*	Drug: : $F_{(1,4)}$ = 0.448, p = 0.5401<br>Pre/post infusion: $F_{(1,4)}$ = 4.153, p = 0.1112<br>Interaction: $F_{(1, 4)}$ = 1.325,<br>p = 0.3138	n = 5
**S8e**	AP5 vs. VEH infusion.<br>Pre vs. post infusion<br>Extended training	S- latency	Two-factor rep. measures ANOVA.<br>*Within-subject:*	Drug: : $F_{(1,4)}$ = 0.002, p = 0.9698	n = 5

			- *Drug*<br>- *Pre/post inf.*	Pre/post infusion: $F_{(1,4)}$ = 1.743, p = 0.2572<br>Interaction: $F_{(1, 4)}$ = 0.116,<br>p = 0.7499	
**S8f**	AP5 vs. VEH infusion.<br>Pre vs. post infusion<br>Extended training	ITI pseudolatency	Two-factor rep. measures ANOVA.<br>*Within-subject:*<br>- *Drug*<br>- *Pre/post inf.*	Drug: : $F_{(1,4)}$ = 0.002, p = 0.9694<br>Pre/post infusion: $F_{(1,4)}$ = 1.324, p = 0.3140<br>Interaction: $F_{(1, 4)}$ = 2.783,<br>p = 0.1706	n = 5
**S8a**	VEH vs. AP5 sides<br>(-105 vs. -71 from CP)	Baseline firing rate (-2000-0 ms pre S+)	Wilcoxon rank sum test (Holm-Sidak corrected)	p = 1	n = 75/<br>n = 66
**S9a**	VEH vs. AP5 sides<br>(-70 vs. -36 from CP)	Baseline firing rate (-2000-0 ms pre S+)	Wilcoxon rank sum test (Holm-Sidak corrected)	p = 0.5617	n = 137/<br>n = 103
**S9a**	VEH vs. AP5 sides<br>(-35 vs. -1 from CP)	Baseline firing rate (-2000-0 ms pre S+)	Wilcoxon rank sum test (Holm-Sidak corrected)	p = 0.5617	n = 129/<br>n = 96
**S9a**	VEH vs. AP5 sides<br>(0 vs. 34 from CP)	Baseline firing rate (-2000-0 ms pre S+)	Wilcoxon rank sum test (Holm-Sidak corrected)	p = 0.5617	n = 131/<br>n = 102
**S9a**	VEH vs. AP5 sides<br>(35 vs. 69 from CP)	Baseline firing rate (-2000-0 ms pre S+)	Wilcoxon rank sum test (Holm-Sidak corrected)	p = 1	n = 92/<br>n = 85
**S9a**	VEH vs. AP5 sides<br>(70 vs. 105 from CP)	Baseline firing rate (-2000-0 ms pre S+)	Wilcoxon rank sum test (Holm-Sidak corrected)	p = 1	n = 36/<br>n = 75
**S9b**	Previously VEH vs. AP5 sides (Extinction test, “learners”)	Baseline firing rate (-2000-0 ms pre S+)	Wilcoxon rank sum test	p = 0.5185	n = 38/<br>n= 39
**S10b**	VEH vs. AP5<br>(-105 to -71 trials from CP)	Firing -2000-0 ms (Z sc.) before S+ entry (when latency > 5 s)	Wilcoxon rank sum test<br>(Holm-Sidak corrected)	p = 0.681	n = 21/<br>n = 15
**S10b**	VEH vs. AP5<br>(-70 to -36 trials from CP)	Firing -2000-0 ms (Z sc.) before S+ entry (when latency > 5 s)	Wilcoxon rank sum test<br>(Holm-Sidak corrected)	p = 0.2246	n = 22/<br>n = 20
**S10b**	VEH vs. AP5<br>(-35 to -1 trials from CP)	Firing -2000-0 ms (Z sc.) before S+ entry (when latency > 5 s)	Wilcoxon rank sum test<br>(Holm-Sidak corrected)	p = 0.0329	n = 25/<br>n = 15
**S10b**	VEH vs. AP5<br>(0 to 34 trials from CP)	Firing -2000-0 ms (Z sc.) before S+ entry (when latency > 5 s)	Wilcoxon rank sum test<br>(Holm-Sidak corrected)	p = 0.0404	n = 28/<br>n = 11
**S10b**	VEH vs. AP5<br>(35 to 69 trials from CP)	Firing -2000-0 ms (Z sc.) before S+ entry (when latency > 5 s)	Wilcoxon rank sum test<br>(Holm-Sidak corrected)	p = 0.0009	n = 15/<br>n = 10
**S10b**	VEH vs. AP5<br>(70 to 105 trials from CP)	Firing -2000-0 ms (Z sc.) before S+ entry (when latency > 5 s)	Wilcoxon rank sum test<br>(Holm-Sidak corrected)	p = 0.0001	n = 4/<br>n = 9
**S10b**	VEH side. Trial bins 1 vs. 3 (-105 to -71 vs.-35 to -1 trials from CP)	Firing -2000-0 ms (Z sc.) before S+ entry (when latency > 5 s)	Wilcoxon rank sum test<br>(Holm-Sidak corrected)	p = 0.1732	n = 21/<br>n = 25
**S10b**	VEH side. Trial bins 3 vs. 5 (-35 to -1 vs. 35 to 69 trials from CP)	Firing -2000-0 ms (Z sc.) before S+ entry (when latency > 5 s)	Wilcoxon rank sum test<br>(Holm-Sidak corrected)	p = 0.0088	n = 25/<br>n = 15
**S10b**	AP5 side. Trial bins 1 vs. 3 (-105 to -71 vs.-35 to -1 trials from CP)	Firing -2000-0 ms (Z sc.) before S+ entry (when latency > 5 s)	Wilcoxon rank sum test<br>(Holm-Sidak corrected)	p = 0.7568	n = 15/<br>n = 15

**S10b**	AP5 side. Trial bins 3 vs. 5 (-35 to -1 vs. 35 to 69 trials from CP)	Firing -2000-0 ms (Z sc.) before S+ entry (when latency > 5 s)	Wilcoxon rank sum test (Holm-Sidak corrected)	p = 0.7694	n = 15/ n = 10
**S10c**	*Before change point session.* VEH vs. AP5	Proportion of S+-entry-excited units	Fisher's exact test for count data (Holm-Sidak corrected)	p = 0.2762	n = 160/ n = 110
**S10c**	*Before change point session.* VEH vs. AP5	Proportion of S+-entry-inhibited units	Fisher's exact test for count data (Holm-Sidak corrected)	p = 0.1455	n = 160/ n = 110
**S10c**	*Before change point session. Entry-excited units.* VEH vs. AP5	Firing rate (0-1500 ms after S+ entry)	Wilcoxon rank sum test	p = 0.9864	n = 33/ n = 29
**S10c**	*Before change point session. Entry-inhibited units.* VEH vs. AP5	Firing rate (0-1500 ms after S+ entry)	Wilcoxon rank sum test	p = 0.4064	n = 62/ n = 37
**S10c**	*After change point session.* VEH vs. AP5	Proportion of S+-entry-excited units	Fisher's exact test for count data (Holm-Sidak corrected)	p = 0.7939	n = 160/ n = 110
**S10c**	*After change point session.* VEH vs. AP5	Proportion of S+-entry-inhibited units	Fisher's exact test for count data (Holm-Sidak corrected)	p = 0.8250	n = 160/ n = 110
**S10c**	*After change point session. Entry-excited units.* VEH vs. AP5	Firing rate (0-1500 ms after S+ entry)	Wilcoxon rank sum test	p = 0.6102	n = 35/ n = 40
**S10c**	*After change point session. Entry-inhibited units.* VEH vs. AP5	Firing rate (0-1500 ms after S+ entry)	Wilcoxon rank sum test	p = 0.5586	n = 63/ n = 69
**S11e**	VEH side: S+ vs. S- (0-35 trial bin)	Firing 100-400 ms after S+ (Z sc.)	Wilcoxon signed-rank test (Holm-Sidak corrected)	p = 0.9788	n = 55
**S11e**	VEH side: S+ vs. S- (36-70 trial bin)	Firing 100-400 ms after S+ (Z sc.)	Wilcoxon signed-rank test (Holm-Sidak corrected)	p = 1	n = 81
**S11e**	VEH side: S+ vs. S- (71-105 trial bin)	Firing 100-400 ms after S+ (Z sc.)	Wilcoxon signed-rank test (Holm-Sidak corrected)	p = 0.0122	n = 86
**S11e**	VEH side: S+ vs. S- (106-140 trial bin)	Firing 100-400 ms after S+ (Z sc.)	Wilcoxon signed-rank test (Holm-Sidak corrected)	p = 1	n = 38
**S11e**	VEH side: S+ vs. S- (141-175 trial bin)	Firing 100-400 ms after S+ (Z sc.)	Wilcoxon signed-rank test (Holm-Sidak corrected)	p = 1	n = 18
**S11e**	VEH side: S+ vs. S- (176-210 trial bin)	Firing 100-400 ms after S+ (Z sc.)	Wilcoxon signed-rank test (Holm-Sidak corrected)	p = 0.0276	n = 26
**S11e**	AP5 side: S+ vs. S- (1-35 trial bin)	Firing 100-400 ms after S+ (Z sc.)	Wilcoxon signed-rank test (Holm-Sidak corrected)	p = 0.6820	n = 38
**S11e**	AP5 side: S+ vs. S- (36-70 trial bin)	Firing 100-400 ms after S+ (Z sc.)	Wilcoxon signed-rank test (Holm-Sidak corrected)	p = 0.6776	n = 24
**S11e**	AP5 side: S+ vs. S- (71-105 trial bin)	Firing 100-400 ms after S+ (Z sc.)	Wilcoxon signed-rank test (Holm-Sidak corrected)	p = 0.6776	n = 36

**S11e**	AP5 side: S+ vs. S- (106-140 trial bin)	Firing 100-400 ms after S+ (Z sc.)	Wilcoxon signed-rank test (Holm-Sidak corrected)	p = 0.6776	n = 25
**S11e**	AP5 side: S+ vs. S- (141-175 trial bin)	Firing 100-400 ms after S+ (Z sc.)	Wilcoxon signed-rank test (Holm-Sidak corrected)	p = 0.5404	n = 20
**S11e**	AP5 side: S+ vs. S- (176-210 trial bin)	Firing 100-400 ms after S+ (Z sc.)	Wilcoxon signed-rank test (Holm-Sidak corrected)	p = 0.1238	n = 15
**S11f**	VEH side: 35 trial bins (sessions)	Proportion of cue-excited neurons	Fisher's exact test for count data	p = 0.0301	n = 304
**S11f**	VEH side: 1-35 vs. 36-70 trial bin.	Proportion of cue-excited neurons	Fisher's exact test for count data (Holm-Sidak corrected)	p = 1	n = 55/ n = 81
**S11f**	VEH side: 36-70 vs. 71-105 trial bin.	Proportion of cue-excited neurons	Fisher's exact test for count data (Holm-Sidak corrected)	p = 1	n = 81/ n = 86
**S11f**	VEH side: 71-105 vs. 106-140 trial bin.	Proportion of cue-excited neurons	Fisher's exact test for count data (Holm-Sidak corrected)	p = 0.7165	n = 86/ n = 38
**S11f**	VEH side: 106-140 vs. 141-175 trial bin.	Proportion of cue-excited neurons	Fisher's exact test for count data (Holm-Sidak corrected)	p = 1	n = 38/ n =18
**S11f**	VEH side: 141-175 vs. 176-210 trial bin	Proportion of cue-excited neurons	Fisher's exact test for count data (Holm-Sidak corrected)	p = 0.0465	n = 18/ n =26
**S11f**	VEH side: 35 trial bins (sessions)	Proportion of cue-inhibited neurons	Fisher's exact test for count data	p = 0.2596	n = 304
**S11f**	AP5 side: 35 trial bins (sessions)	Proportion of cue-excited neurons	Fisher's exact test for count data	p = 0.5149	n = 158
**S11f**	AP5 side: 35 trial bins (sessions)	Proportion of cue-inhibited neurons	Fisher's exact test for count data	p = 0.164	n = 158
**S11f**	VEH vs. AP5 side: 1-35 trial bin.	Proportion of cue-excited neurons	Fisher's exact test for count data (Holm-Sidak corrected)	p = 1	n = 55/ n = 38
**S11f**	VEH vs. AP5 side: 36-70 trial bin.	Proportion of cue-excited neurons	Fisher's exact test for count data (Holm-Sidak corrected)	p = 1	n = 81/ n = 24
**S11f**	VEH vs. AP5 side: 71-105 trial bin.	Proportion of cue-excited neurons	Fisher's exact test for count data (Holm-Sidak corrected)	p = 1	n = 86/ n = 36
**S11f**	VEH vs. AP5 side: 106-140 trial bin.	Proportion of cue-excited neurons	Fisher's exact test for count data (Holm-Sidak corrected)	p = 1	n = 38/ n = 25
**S11f**	VEH vs. AP5 side: 141-175 trial bin.	Proportion of cue-excited neurons	Fisher's exact test for count data (Holm-Sidak corrected)	p = 1	n = 18/ n = 20
**S11f**	VEH vs. AP5 side: 176-210 trial bin.	Proportion of cue-excited neurons	Fisher's exact test for count data (Holm-Sidak corrected)	p = 1	n = 26/ n = 15
**S11f**	VEH vs. AP5 side: 1-35 trial bin.	Proportion of cue-inhibited neurons	Fisher's exact test for count data (Holm-Sidak corrected)	p = 1	n = 55/ n = 38
**S11f**	VEH vs. AP5 side: 36-70 trial bin.	Proportion of cue-inhibited neurons	Fisher's exact test for count data (Holm-Sidak corrected)	p = 0.1671	n = 81/ n = 24
**S11f**	VEH vs. AP5 side: 71-105 trial bin.	Proportion of cue-inhibited neurons	Fisher's exact test for count data (Holm-Sidak corrected)	p = 0.0579	n = 86/ n = 36
**S11f**	VEH vs. AP5 side: 106-140 trial bin.	Proportion of cue-inhibited neurons	Fisher's exact test for count data (Holm-Sidak corrected)	p = 1	n = 38/ n = 25
**S11f**	VEH vs. AP5 side: 141-175 trial bin.	Proportion of cue-inhibited neurons	Fisher's exact test for count data (Holm-Sidak corrected)	p = 1	n = 18/ n = 20

**S11f**	VEH vs. AP5 side: 176-210 trial bin.	Proportion of cue-inhibited neurons	Fisher's exact test for count data (Holm-Sidak corrected)	p = 0.7728	n = 26/ n = 15
**S11h**	VEH vs. AP5 side	Proportion of cue-excited neurons	Fisher's exact test for count data	p = 1	n = 26/ n = 15

Supplementary References

1. Gallistel, C. R., Fairhurst, S. & Balsam, P. The learning curve: Implications of a quantitative analysis. *PNAS* **101**, 13124–13131 (2004).

2. Paxinos, G. & Watson, C. The Rat Brain in Stereotaxic Coordinates. (Academic Press, 2007).
