# Supplementary material for: NMDA receptor-dependent plasticity in the nucleus accumbens connects reward-predictive cues to approach responses
Source: Nat Commun. 2019 Sep 27;10:4429. doi: 10.1038/s41467-019-12387-z (PMC6764993; doi:10.1038/s41467-019-12387-z)
Supplement: Supplementary file 2 — Reporting Summary [file 41467_2019_12387_MOESM2_ESM.pdf]

nature research

Corresponding author(s): Saleem M. Nicola

Last updated by author(s): Sep 4, 2019

Reporting Summary

Nature Research wishes to improve the reproducibility of the work that we publish. This form provides structure for consistency and transparency in reporting. For further information on Nature Research policies, see Authors & Referees and the Editorial Policy Checklist.

Statistics

For all statistical analyses, confirm that the following items are present in the figure legend, table legend, main text, or Methods section.

n/a	Confirmed	
☐	☒	The exact sample size (*n*) for each experimental group/condition, given as a discrete number and unit of measurement
☐	☒	A statement on whether measurements were taken from distinct samples or whether the same sample was measured repeatedly
☐	☒	The statistical test(s) used AND whether they are one- or two-sided<br>*Only common tests should be described solely by name; describe more complex techniques in the Methods section.*
☒	☐	A description of all covariates tested
☐	☒	A description of any assumptions or corrections, such as tests of normality and adjustment for multiple comparisons
☐	☒	A full description of the statistical parameters including central tendency (e.g. means) or other basic estimates (e.g. regression coefficient) AND variation (e.g. standard deviation) or associated estimates of uncertainty (e.g. confidence intervals)
☐	☒	For null hypothesis testing, the test statistic (e.g. *F*, *t*, *r*) with confidence intervals, effect sizes, degrees of freedom and *P* value noted<br>*Give P values as exact values whenever suitable.*
☒	☐	For Bayesian analysis, information on the choice of priors and Markov chain Monte Carlo settings
☒	☐	For hierarchical and complex designs, identification of the appropriate level for tests and full reporting of outcomes
☒	☐	Estimates of effect sizes (e.g. Cohen's *d*, Pearson's *r*), indicating how they were calculated

Our web collection on statistics for biologists contains articles on many of the points above.

Software and code

Policy information about availability of computer code

Data collection	Data collection was conducted using existing software: Med-PC IV (Med Associates), SortClient and Offline Sorter (Plexon).
Data analysis	Data was prepared for analysis using NeuroExplorer (Plexon) and analyzed using custom routines in R. Figures were assembled using Adobe Illustrator CC 2017. The custom code used in this study is publicly available in the following repository: https://github.com/mvegavillar/Accumbens-Rew-learning.

For manuscripts utilizing custom algorithms or software that are central to the research but not yet described in published literature, software must be made available to editors/reviewers. We strongly encourage code deposition in a community repository (e.g. GitHub). See the Nature Research guidelines for submitting code & software for further information.

Data

Policy information about availability of data

All manuscripts must include a data availability statement. This statement should provide the following information, where applicable:

- Accession codes, unique identifiers, or web links for publicly available datasets
- A list of figures that have associated raw data
- A description of any restrictions on data availability

The datasets that support the findings of the current study are available from the corresponding author upon request.

Field-specific reporting

Please select the one below that is the best fit for your research. If you are not sure, read the appropriate sections before making your selection.

☒ Life sciences ☐ Behavioural & social sciences ☐ Ecological, evolutionary & environmental sciences

For a reference copy of the document with all sections, see nature.com/documents/nr-reporting-summary-flat.pdf

Life sciences study design

All studies must disclose on these points even when the disclosure is negative.

Sample size	Sample size was not predetermined using statistical power analyses. Instead, sample sizes were decided based on those used by similar studies to detect similar effects.
Data exclusions	Animals that did not acquire the target conditioned behavior within the training period ("non-learners") were excluded from analyses that examined learning-related behavioral and neuronal firing changes. Data collected from those subjects is shown in Supplementary Fig. 11.
Replication	To the extent that different cohorts of animals within each experiment yielded comparable results, we believe the results of this study are replicable.
Randomization	Subjects were randomly allocated to the experimental groups. In the experiments in which drugs were infused unilaterally into the brain, the assignment of brain hemispheres to the experimental (drug-infused) or control (saline-infused) groups was random.
Blinding	Investigators were not blinded during data collection because it would be practically unfeasible (i.e., drug and saline are easily distinguishable visually). Investigators were blinded during data analysis (i.e., the same code was applied to all files and the output of the code —graphs and analyses— were labeled automatically).

Reporting for specific materials, systems and methods

We require information from authors about some types of materials, experimental systems and methods used in many studies. Here, indicate whether each material, system or method listed is relevant to your study. If you are not sure if a list item applies to your research, read the appropriate section before selecting a response.

Materials & experimental systems

n/a	Involved in the study	
☒	☐	Antibodies
☒	☐	Eukaryotic cell lines
☒	☐	Palaeontology
☐	☒	Animals and other organisms
☒	☐	Human research participants
☒	☐	Clinical data

Methods

n/a	Involved in the study	
☒	☐	ChIP-seq
☒	☐	Flow cytometry
☒	☐	MRI-based neuroimaging

Animals and other organisms

Policy information about studies involving animals; ARRIVE guidelines recommended for reporting animal research

Laboratory animals	Male Long-Evans rats, 3-4 months old (~ 400 g)
Wild animals	The study did not involve wild animals.
Field-collected samples	The study did not involve field-collected samples.
Ethics oversight	Institutional Animal Care and Use Committee at Albert Einstein College of Medicine (The Bronx, New York).

Note that full information on the approval of the study protocol must also be provided in the manuscript.
